# Medicine quality assessment in Nepal using semi randomised sampling and evaluation of a small scale dissolution test and portable Raman spectrometers

**DOI:** 10.1038/s41598-025-16340-7

**Published:** 2025-08-21

**Authors:** Robin Schreiber, Md. Ahsanul Haque, Mohammad Sofiqur Rahman, Bhupendra Kumar Poudel, Balmukunda Regmi, Kazuko Kimura, Naoko Yoshida

**Affiliations:** 1 Clinical Pharmacy and Healthcare Sciences, Division of Pharmaceutical Sciences, Graduate School of Medical Sciences, Kanazawa University, Kakuma-machi, Kanazawa, Ishikawa 920-1192 Japan; 2 Graduate School of Medical Sciences, Medi-Quality Security Institute, Kanazawa University, Kakuma-machi, Kanazawa, Ishikawa 920-1192 Japan; 3 Stark Neuroscience Research Institute, Indiana University, Indianapolis, IN 46202 USA; 4 Department of Clinical Pharmacy, School of Pharmacy, University of California, 521 Parnassus Ave, San Francisco, CA 94143 USA; 5 Western Regional Hospital, Pokhara Academy of Health Sciences, Pokhara, Nepal; 6 Department of Pharmacy, Maharajgunj Medical Campus, Institute of Medicine, Tribhuvan University, Kathmandu, Nepal; 7 Society for Medicines Security Research, 4F Venture Business Laboratory, Kanazawa University Kakuma-machi, Kanazawa, Ishikawa 920-1192 Japan; 8 AI Hospital/Macro Signal Dynamics Research and Development Center, Institute of Medical, Pharmaceutical and Health Sciences, Kanazawa University, Kakuma-machi, Kanazawa, Ishikawa 920-1192 Japan

**Keywords:** Quality, Substandard and falsified medicines, Nepal, Screening technology, Dissolution test, Portable Raman scattering analysis, Drug regulation, Health policy, Public health

## Abstract

Substandard and falsified medicines threaten global health and require reliable data and screening technologies to combat their spread. This study examined the quality of 241 samples containing azithromycin, cefixime, esomeprazole and losartan collected from licenced private vendors in the Saptari (121 samples; convenience sampling) and Kathmandu (120 samples; randomised sampling) districts of Nepal. Nearly 10% (24 samples; 95% CI 6.5–14.5) of samples failed pharmacopoeial quality analysis and were classified as ‘substandard’ or ‘probably substandard’. No falsified medicines were identified. Small-scale dissolution acceptance criteria were applied to all 20 three-unit combinations of 213 samples tested in the first stage of the United States Pharmacopoeia dissolution test. Approximately 1% of these results were false positives when compared with the final United States Pharmacopoeia dissolution test results, suggesting the test’s usefulness in encouraging dissolution testing in resource-limited contexts. In the narrow sense of presence/absence, two portable Raman spectrometers reliably detected azithromycin, cefixime and losartan in most samples based on effective methods for detecting falsified medicines; however, none of the substandard samples were identified. The findings suggest that falsified medicines are less prevalent in Nepal and the surrounding region than suggested by regional concerns about Nepal and global concerns about low- and middle-income countries. Nevertheless, the Nepalese government should continue to ensure the quality of all distributed medicines.

**Supplementary Information:**

The online version contains supplementary material available at 10.1038/s41598-025-16340-7.

## Introduction

Nearly one-third of the global population still lacks access to essential medicines, especially in low- and middle-income countries (LMICs)^1–3^. The full realisation of the human right to health remains a major global challenge despite the United Nations member states’ adoption of target 3.8 of the Sustainable Development Goals in 2015^4–6^. The target links access to safe, effective, high-quality and affordable essential medicines for all people with the realisation of universal health coverage^4,5,7^. However, improvements in health service coverage have stagnated since the Sustainable Development Goals officially came into force^5^.

The World Health Organisation (WHO) estimates that about one in ten (10.5%) of medicines in LMICs are substandard and falsified (SF) medicines^8^. The World Health Assembly 2017 agreed on the following definition of SF medicines as mutually exclusive categories: 1. ‘Substandard (or ‘out-of-specification’) medicines are authorised medical products that fail to meet either their quality standards, specifications, or both’ and 2. ‘Falsified medicines are medical products that deliberately or fraudulently misrepresent their identity, composition, or source’^9,10^. SF medicines can lead to harm, treatment failure and even death of the consumer^10–15^. These medicines further cause a loss of trust in medicines and healthcare stakeholders and pose a significant economic burden^9,10,12–16^. The cost of SF medicines is estimated at USD 10–200 billion annually^14,16^. The available data suggest that substandard medicines may be six times more prevalent as a global average than falsified medicines in LMICs^17^. However, international media focus more on the reporting of falsified high-priced products^18^. Nevertheless, the spread of falsified medicines has been described as a pandemic and has been reported more frequently in recent years^19^.

The South Asian country of Nepal, with a population of roughly 30 million people, is landlocked in the Himalayas^20^. Despite a significant and rapid reduction in general poverty in recent years, Nepal remains one of the slowest-growing economies in Asia and is classified as a lower-middle-income country^3,21^. The country is bordered by China and India, both of which were named ‘global primary producers of falsified medicines’ by the Organisation for Economic Co-operation and Development (OECD) in 2020^22^. While Nepal maintains limited, monitored trade with China, its open, visa-free border with India allows unrestricted movement of people and goods^23^. Nepal relies on imports of medical products, raw materials and packaging materials from both neighbour countries, according to the Department of Drug Administration (DDA; March 2023).

The spread of SF medicines in a country is known to be strongly inversely linked to the strength of its healthcare system^18,24,25^. Nepal’s medicine regulatory system is currently classified at WHO maturity level 1, i.e. as having limited capacity^26,27^. Additionally, Nepal’s hot and humid climate, which is categorised as climatic zone IVb by international pharmaceutical standards^28,29^, requires suitable storage conditions to preserve the quality of medicines. Reliable epidemiological data are required to effectively combat SF medicines and their dissemination, but such data remain limited worldwide^10,14,17,30–32^. To date, no study has assessed the pharmaceutical quality of medicines in Nepal’s private sector using both pharmacopoeial tests and field-based screening tools.

In LMICs, including Nepal, quality tests such as the dissolution test are often neglected given capacity limitations. However, they play a critical role in preventing adverse effects from the use of poor-quality medicines such as treatment failure and increased antimicrobial resistance^9,10,12–16,33^. Dissolution and drug-release tests are expected to play a further important role in the future regulation of medicine quality^34^, necessitating even more worldwide testing capacities. A novel small-scale dissolution test recently developed by Rahman and Yoshida et al. reduces required testing capacity compared to the USP dissolution test^35^. To assess the dissolution performance of a sample, it applies modified compliance criteria of the USP dissolution test to three tested units: the average dissolution rate (Q) of the three units is not less than or equal to Q + 6%, and no individual Q is less than or equal to Q + 2%, while maintaining the USP test conditions^35^. However, the small-scale dissolution test has not yet been field tested, and no data are available on its comparability to the USP dissolution test.

Screening technologies to identify SF medicines on-site are urgently needed to safeguard pharmaceutical supply chains, especially in resource-limited contexts. They can also help reduce the number of samples requiring costly laboratory analysis. Portable Raman spectrometers are analytical devices that use laser-based spectroscopy to identify the molecular composition of substances based on their Raman scattering signatures. These instruments are relatively inexpensive, potentially non-destructive and have previously been shown to detect falsified medicines and unlicensed products^36–41^, making them potentially useful screening technologies. However, they have so far been unable to detect substandard medicines, limiting their broader applicability.

This semi-randomised, cross-sectional study in the Saptari (convenience sampling) and Kathmandu (randomised sampling) districts investigated the quality of medicines sold by Nepalese pharmacies and legal retail shops of the private sector (hereinafter referred to as ‘licenced vendors’). The study focused on pharmaceutical products that contained one of four selected active pharmaceutical ingredients (APIs): azithromycin (AZM), cefixime (CFIX), esomeprazole (ESM) and losartan (LST). The results were intended to identify improvements for manufacturers, the Nepalese regulatory authorities and the Nepalese government. The findings could also support the development of effective strategies to combat the problem of SF medicines and improve and ensure medicine quality in Nepal. In addition, the study assessed the applicability of a small-scale dissolution test and two portable Raman spectrometers as potential screening technologies for the effective detection of SF medicines.

## Methods

### Chemicals and materials

The AZM dihydrate standard (#PHR1088, LOT: LRAC6480, purity: 94.6% as dihydrate) and the LST potassium standard (#PHR1602, LOT: LRAC5655, purity: 99.9%) were purchased from Sigma-Aldrich Chemie GmbH (Steinheim, Germany). The omeprazole standard (#217087-09-7, LOT: J0H412, purity: 99.9%) was purchased from The United States Pharmacopoeial Convention (USP; North Bethesda, MD, USA). The CFIX trihydrate standard (#034-25261, LOT: SKE6566, purity: 99.0%), lansoprazole (#129-05863, LOT: CAE1292, purity: 99.8%), metronidazole (#139-14931, LOT: DLG2869, purity: minimum 98.0%), benzophenone (#023-01072, LOT: CTP1333, purity: minimum 98.0%), diclofenac sodium (#043-22851, LOT: LEM5673, purity: minimum 98.0%), K_2_HPO_4_, H_3_PO_4_, MeCN and MeOH were purchased from FUJIFILM Wako Pure Chemical Corporation (Osaka, Japan). All of the solvents were high-performance liquid chromatography (HPLC)-grade. NaH_2_PO_4_ × 2 H_2_O, Na_2_HPO_4_, Na_3_PO_4_ × 12 H_2_O, 5M HCl, and 5M and 10M NaOH solutions were purchased from Nacalai Tesque, Inc. (Kyoto, Japan). KH_2_PO_4_ was purchased from FUJIFILM Wako Pure Chemical Corporation (Osaka, Japan) and Nacalai Tesque, Inc. (Kyoto, Japan).

The AZM standard product Zithromac 250 mg (tablets) was purchased from Pfizer Inc. (New York, NY, USA), the CFIX standard product Cefspan 100 mg (capsules) was purchased from Choseido Pharmaceutical Co., Ltd. (Tokushima, Japan), the ESM standard product Nexium 20 mg (capsules) was provided by AstraZeneca PLC (Cambridge, UK) and the LST standard product NU-LOTAN 50 mg (tablets) was purchased from Organon & Co. (Jersey City, NJ, USA).

### Devices and instrumentation

HPLC analysis was performed at Kanazawa University in Kanazawa, Ishikawa, Japan using HPLC systems from three manufacturers. A summary of the chromatographic conditions is given in Table 1. For the analysis of AZM and LST, a system by Hitachi High-Tech Science Corporation (Tokyo, Japan) that was equipped with a Hitachi Elite LaChrom Organizer, an Elite LaChrom L-2130 pump, an Elite LaChrom L-2200 autosampler, an Elite LaChrom L-2300 column oven and an Elite LaChrom L-2455 diode array detector was used. AZM was analysed using GL Sciences Inc.’s (Tokyo, Japan) InertSustain C18 4.6 mm I.D. × 150 mm (5-µm particle) packing L1 column. The system for analysing LST was equipped with the Mightysil RP-18 GP 4.6 mm I.D. × 150 mm (5-µm particle) Cica reagent column by Kanto Chemical Co., Inc. (Tokyo, Japan). ChromAssist Data Station Version 3.0 by Hitachi High-Tech Science Corporation (Tokyo, Japan) was used as a software/digital workstation for data acquisition. For the analysis of CFIX, a system by Shimadzu Corporation (Kyoto, Japan) was equipped with a CBM-20A prominence communications bus module, an LC-40D pump (pump A), an LC-10AD pump (pump B), a SIL-10A XL auto-injector, a CTO-20AC prominence column oven and an SPD-M20A prominence photodiode array detector. CFIX was analysed using the Shim-pack CLC-ODS (M) 4.6 mm I.D. × 150 mm (5-µm particle) RP18 column by Shimadzu Corporation (Kyoto, Japan). Shimadzu Corporation’s (Kyoto, Japan) Shimadzu LabSolutions was used as a software/digital workstation for data acquisition. For the analysis of ESM, a system by Jasco, Inc. (Tokyo, Japan) equipped with an LC-Net II/ADC instrument, a PU-4180 pump, an AS-4550 autosampler, a CO-1560 column oven, a UV2075 Plus UV/VIS detector, a MD-2018Plus photodiode array detector, and a CD-2095 chiral detector was used. ESM was analysed using the Phenomenex 4.6 mm I.D. × 150 mm (5-µm particle) NX-C18 column by Phenomenex Inc. (Torrance, CA, USA). ChromNAV 2 by Jasco, Inc. (Tokyo, Japan) was used as a software/digital workstation for data acquisition.

**Table 1:** Overview of the chromatographic conditions.

API	Column	Injection volume	Mobile phase	Flow rate		Oven temperature	Detection wavelength
AZM	InertSustain C18 4.6 mm I.D. × 150 mm (5-μm particles) L1 C18 column GL Sciences Inc (Tokyo, Japan)	20 µL	35% of A: pH 7.5 buffer 65% of B: acetonitrile (v/v)	0.9 mL/ min		45 °C	210 nm
CFIX	Shim-pack CLC-ODS (M) 4.6 mm I.D. × 150 mm (5-μm particles) C18 column Shimadzu Corporation (Kyoto, Japan)	10 µL	72.5% of A: pH 6.86 buffer 27.5% of B: acetonitrile (v/v)	1.0 mL/ min		40 °C	288 nm: Cefixime 254 nm: internal standard
ESM	Phenomenex NX-C18 4.6 mm I.D. × 150 mm (5-μm particles) C18 column Phenomenex Inc (Torrance, CA, USA)	10 µL	40% of A: pH 7.3 buffer 60% of B: acetonitrile (v/v)	0.8 mL/ min		40 °C	302 nm
LST	Mightysil RP-18 GP 4.6 mm I.D. × 150 mm (5-μm particles) C18 column Kanto Chemical Co., Inc. (Tokyo, Japan)	10 µL	50% of A: pH 4.0 buffer 50% of B: acetonitrile (v/v)	0.9 mL/ min		35 °C	250 nm

Dissolution tests were performed using two Toyama NTR-VS6P dissolution testers and one Toyama NTR-6100 dissolution tester by Toyama Sangyo Co., Ltd. (Osaka, Japan) that were each equipped with six 1000 mL dissolution vessels and either six USP dissolution apparatus type 1 baskets (for CFIX) or six USP dissolution apparatus type 2 paddles (for AZM, ESM and LST) in conformance with USP 41.

Raman scattering analysis was conducted using a C13560 ultra-compact, portable Raman spectrometer (96 mm × 14.5 mm × 60 mm; 90 g) manufactured by Hamamatsu Photonics K.K. (Shizuoka, Japan) with a silicon substrate provided by the manufacturer^38^ and an Inspector500 portable Raman spectrometer manufactured by SciAps Inc. (Laramie, WY, USA) with a polystyrene standard provided by the manufacturer. C13560 operation software and NuSpec Pro software were supplied by Hamamatsu Photonics K.K. (Shizuoka, Japan) and SciAps Inc. (Laramie, WY, USA), respectively, and corresponding drivers were installed on a personal computer before the analysis. The Unscrambler X 10.5 by CAMO Software AS (Oslo, Norway) was used for data analysis and visualisation.

The calculation of HPLC results, statistical analysis, and data visualisation were performed using Excel MSO 365 (Microsoft Corp., Redmond, WA, USA).

### Sampling design

#### Active pharmaceutical ingredient selection criteria

AZM, CFIX, ESM and LST were selected because these medications are commonly sold by Nepalese licenced vendors, they can harm patients if drug quality does not meet pharmaceutical standards, and, except for ESM, they are included in the National List of Essential Medicines Nepal^42^. ESM was selected for its higher market price compared with omeprazole (which is included in the National List of Essential Medicines Nepal) and the resulting anticipated high criminal activity associated with this active ingredient.

#### Sample size selection criteria

Based on Cochran’s formula, with parameters set to π = 0.105 (the estimated population proportion of SF medicines), e = 0.05 (the desired margin of error), κ = 1.96 (corresponding to a 95% confidence level), the minimum required number of collected samples was 145; however, the target was set at 240 samples—120 samples from each district, including 60 samples of each API. Samplers were permitted to make slight adjustments if necessary.

#### District selection criteria

The Saptari district was selected given its geographical location near India, its distance to the Nepalese capital city of Kathmandu and as it has a relatively high population density^43^.

The Kathmandu district was chosen because it is Nepal’s capital city, is centrally located geographically, is easily accessible for sampling and represents the most populous and economically important district in Nepal.

#### Sampling site selection criteria

All licenced vendors including wholesalers with affiliated licenced vendors within the selected districts were eligible for sampling.

#### Selection of sampling sites

For the randomisation of sampling sites within a district, an official registration list of licenced vendors that comprised shop names, addresses and other shop-related information was requested from responsible regional offices in the Saptari and Kathmandu districts. However, no official registration list was available from the national or regional Nepalese government institutions responsible for the Saptari district. Therefore, conducting a list-randomisation of sampling sites in the Saptari district was impossible.

For the Kathmandu district, the official registration list contained more than 3,000 licenced vendors. This list was transferred to Microsoft Excel, formatted, and arranged as a top-to-bottom list. Subsequently, the Microsoft Excel ‘= RAND()’ function was used to generate random numbers in the columns adjacent to the information for each sampling site. A filter was used to sort the rows of random numbers from 1 to *n* with the sampling site information, resulting in a randomised table. This randomised table of sampling sites was used as a top-to-bottom priority list.

The priority list, which was used to select the first 360 sampling sites (priority numbers 1 to 360) as the study’s target sampling sites, was communicated to the samplers. Samplers were instructed to strictly visit the sampling sites according to the priority list from lower numbers to higher numbers starting from 1. The samplers were divided into two groups: Team C and Team D. Teams C and D were instructed to collect samples from odd- and even-numbered sampling sites, respectively. If a licenced vendor was closed or non-operational, samplers were allowed to visit a nearby licenced vendor, even if the shop was not included in the priority list. However, the next sampling site visited was required to continue following the priority list. Written instructions containing this information were distributed with the priority list to the samplers before sampling started in the Kathmandu district.

#### Sampling preparation

All the samplers were healthcare workers and pharmacy students who underwent two training sessions. Held before sampling began in the two districts, the sessions covered the terms, criteria and details of the sampling procedure. Physical copies of the training materials were distributed to all the samplers. Researchers supervised the samplers during the sampling process.

In the Saptari district, the samplers reported a low density of licenced vendors. To achieve a sufficient geographical distribution of sampling sites, samplers were split into two groups—Team A and Team B—that travelled to several towns within the Saptari district to collect samples from all licenced vendors that were visually identified along the road. During this process, no licenced vendor that was identified along the road was skipped. During sampling in the Kathmandu district, samplers who were split into Teams C and D reported that some licenced vendors on the priority list were either temporarily or permanently closed or had been renamed, and others were difficult, inefficient or impossible to access given heavy traffic volume; these factors made strict adherence to the priority list difficult. Therefore, the teams collected samples only from the sampling sites on the priority list that were accessible.

#### Sampling terms and criteria

The sampling terms included the requirements that the same API not be collected twice from the same sampling site and that each sample consists of a minimum of 30 dosage units. In cases in which multiple products containing the same API were available, the cheapest product was acquired. However, samplers were advised to avoid repeated purchases of the same product—particularly those from the same batch—although this practice was not explicitly prohibited.

In the Saptari district, samplers were allowed to collect four samples per sampling site using convenience sampling given the unavailability of an official list of licenced vendors and the low density of licenced vendors. In the Kathmandu district, the collection of two samples per sampling site following a randomised approach was required.

Sampling was conducted using a ‘mystery shopper’ method^44,45^ in which samplers were asked to enter the sampling sites in pairs, purchase the samples and collect packaging and leaflets, if available while pretending to be casual customers.

Temperature and humidity were secretly recorded inside and outside the sampling sites using a hygro-thermometer, and the number of pharmacists and workers was counted after the sample was received. Additionally, the samplers were required to query vendors about the number of units of each of the sampled medicines sold monthly at the sampling site. The collected information was documented with other sample-specific documentation in a sampling form (Annex 1) after the sampling site was left. The samples and the corresponding sampling forms were stored together in separate plastic zip lock bags and transported to an air-conditioned car to Tribhuvan University in Kathmandu, Nepal, where they were stored under appropriate storage conditions until transportation to Kanazawa University, Japan. After sample acquisition, the samples were stored under suitable conditions at Tribhuvan University in Kathmandu and Kanazawa University in Kanazawa.

#### Definition of the sample identification code

The sample identification codes (IDs) expressed as ‘X-YZ(-2)’ were assigned according to the sampling team, API and a consecutive API count. ‘X’ represented the sampling teams A, B, C or D, with Teams A and B sampling in the Saptari district and Teams C and D sampling in the Kathmandu district. ‘Y’ indicated the labelled API (1: ESM, 2: LST, 3: CFIX and 4: AZM), and the API count ‘Z’ was consecutively numbered from 1 to 15 to provide an overview for the sampling team of the number of samples that were collected per API. In addition, Sample B-115-2 was collected from the same sampling site as sample B-115 and was the 16^th^ sample collected by sampling Team B in the Saptari district.

#### Mapping the sampling sites

**Fig. 1:**
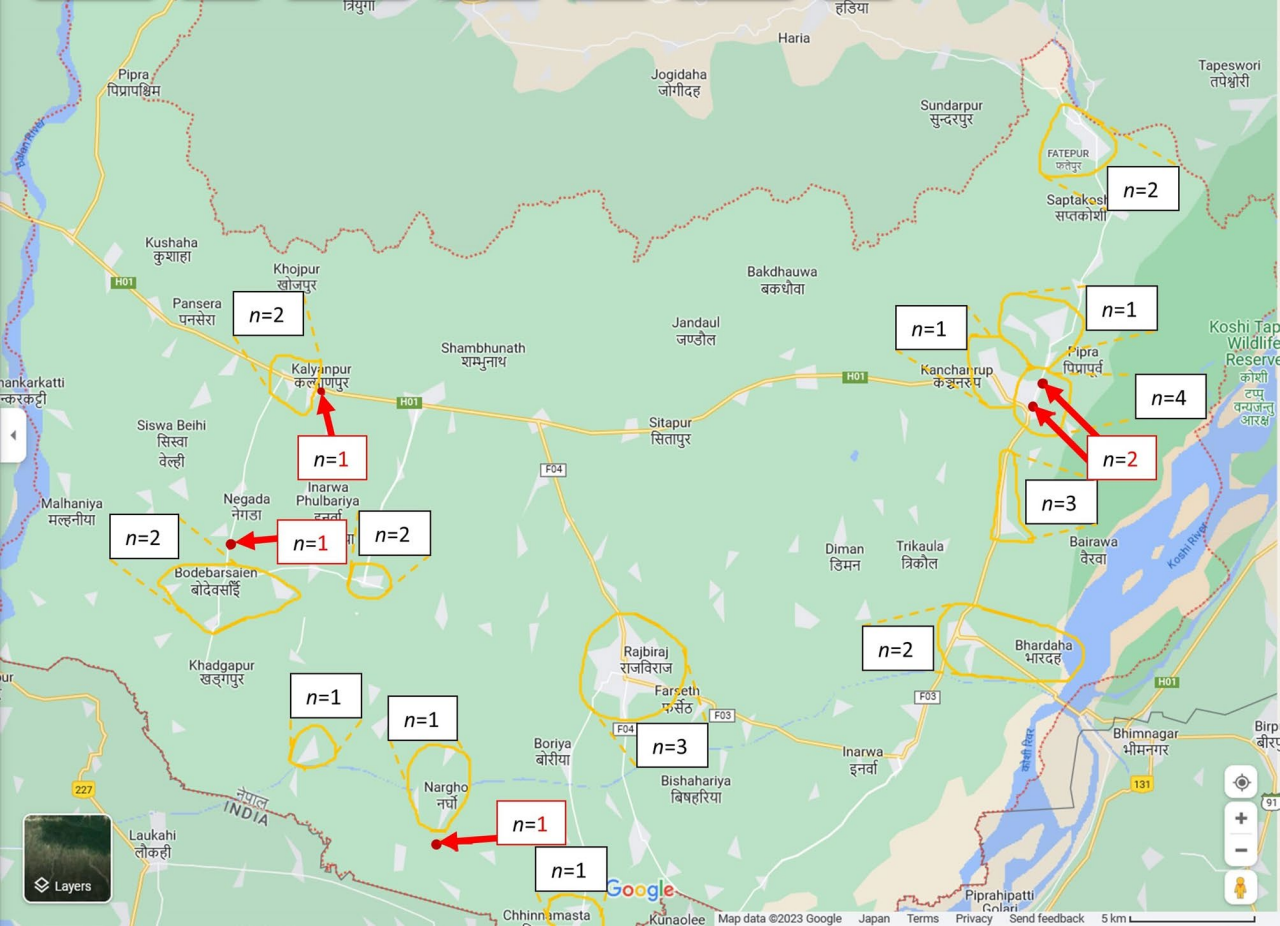
Map of the Saptari district showing the sampling sites and approximate areas in which sampling sites were visited. Shows the sampling map of the Saptari district. The red arrows indicate the five localised sampling sites and the orange circles and nearby *n* = x markings indicate sampling areas that include *n* sampling sites. These circles contain a total of 25 sampling sites as documented in the sampling form, which could not be localised. Samplers did not document the address nor the area of one sampling site, which consequently could not be mapped. The source of the map data was Google Maps ©2023 (Mountain View, CA, USA). The base map was extracted from this using Microsoft Snipping Tool version 2022.2410.21.0 (Microsoft Corp., Redmond, WA, USA). Sampling locations and areas were marked using Microsoft Paint version 11.2410.28.0 (Microsoft Corp., Redmond, WA, USA), and lines and text fields were added using Microsoft Word MSO 365 version 2411 (Microsoft Corp., Redmond, WA, USA).

**Fig. 2:**
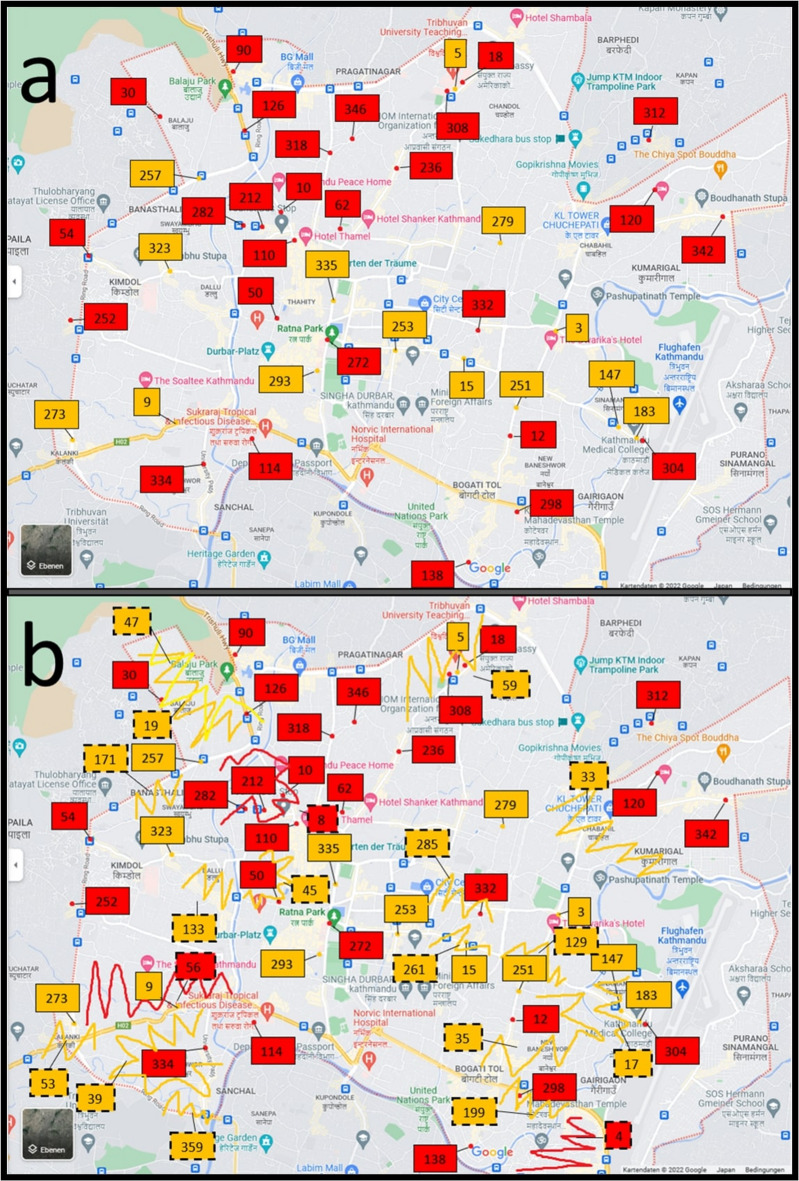
Map of the Kathmandu district showing the individual sampling sites and approximate areas in which sampling sites were visited. Shows a map of the Kathmandu district in which the locations of sample acquisition are indicated. Sampling sites that could be localised are shown as dots with connected boxes (continuous frame). Hatched areas with connected boxes (dashed frame) each represent one sampling site for which only the area could be localised. The boxes contain the corresponding priority number of the sampling site. The orange colour indicates that the sampling was done by Team C, whereas the red colour indicates sites that were visited by Team D. The source of the map data was Google Maps ©2023 (Mountain View, CA, USA). The base map was extracted from this using Microsoft Snipping Tool version 2022.2410.21.0 (Microsoft Corp., Redmond, WA, USA). Sampling locations and areas were marked using Microsoft Paint version 11.2410.28.0 (Microsoft Corp., Redmond, WA, USA), and lines and text fields were added using Microsoft Word MSO 365 version 2411 (Microsoft Corp., Redmond, WA, USA). (**a**): localised sampling sites and (**b**) localised sampling sites and hatched areas corresponding to one sampling site each, which were documented as area only.

The Google Maps (Mountain View, CA, USA) search engine was used to locate individual sampling sites which were indicated on the sampling forms. The identified sampling site locations and areas were mapped in Figs. 1 and 2 using Microsoft Snipping Tool version 2022.2410.21.0 (Microsoft Corp., Redmond, WA, USA) to extract the base map from Google Maps; Microsoft Paint version 11.2410.28.0 (Microsoft Corp., Redmond, WA, USA) to indicate sampling locations and areas; and Microsoft Word MSO 365 version 2411 (Microsoft Corp., Redmond, WA, USA) to add lines, arrows, and text fields.

If samplers documented areas or neighbourhoods which included sampling sites without specifying addresses, the sampling sites were only mapped under specific conditions: the shop identified during the Google search had to have the same shop name in Nepali as indicated on the priority list, its location had to be within the documented neighbourhoods or areas provided by the samplers and the Google Maps representation was required to show an image with the correct shop name of the sampling site for confirmation.

For sampling site locations which could not be located using this method, the documented neighbourhoods or areas were indicated as encircled areas of the Saptari district and hatched areas of the Kathmandu district.

#### Authentic product acquisition

Authentic products which corresponded to the collected sample products were purchased directly from manufacturers after sampling and were received before March 14, 2023. Next, the products were transported to Kanazawa University, Japan and stored under suitable conditions. Manufacturers were informed about the study purpose before the purchase or collection of the authentic products.

#### Visual observation test

All of the 241 samples were visually examined using the ‘tool for visual inspection of medicines’ produced by the International Council of Nurses in partnership with the USP and modified by the International Pharmaceutical Federation^46^. Detailed information from the labelling, packaging, blisters, dosage units and leaflets, if available, was carefully recorded and documented, and images were taken of each sample. Although visual defects may suggest violations of Good Manufacturing Practices, they were not included in the definition of ‘substandard’ in this study, as doing so could be misleading and inconsistent with pharmacopoeial standards, which define substandard medicines based on chemical and performance criteria.

#### Authenticity investigation

All available email and physical addresses of the labelled manufacturers were collected from the sample blisters and packages, the manufacturers’ websites, social media appearances on Facebook (Meta Platforms, Inc., Cambridge, MA, USA), LinkedIn (Sunnyvale, CA, USA) and X (formerly Twitter, X Corp., San Francisco, CA, USA) and advertisements and job offers published by the manufacturers using the Google (Mountain View, CA, USA) search engine’s text and image search feature.

Authenticity investigation forms were compiled by recording the samples’ brand or trade name, stated manufacturer and distributor, dosage form and strength, quality specifications as per the labelled pharmacopoeia, batch number and manufacturing and expiry dates. All relevant images of the sample, including the packaging, blisters and any problematic dosage units were attached to the form. Emails with the corresponding authenticity investigation form, a cover letter and the request to return completed authenticity forms were sent to all of the 36 manufacturers.

Manufacturers who did not respond to the initial email for 14 days were classified as non-responder type 1 and a reminder email was sent. A manufacturer was classified as non-responder type 2 if a completed authenticity form was not received within 6 months of sending the reminder email. Subsequently, the authenticity forms of all manufacturers classified as non-responder type 2 were printed and dispatched together with the cover letter and the request to return the completed authenticity form by Japan Post’s Express Mail Service. Manufacturers who did not answer the postal authenticity enquiry within 6 months were classified as non-responder type 3.

The dispatch dates of all authenticity enquiry emails and the posting and receipt dates of the completed authenticity forms were recorded to investigate the authenticity response time. The response time was defined as the income difference between the dispatch date and the response receipt date. The minimum, median and maximum response times were determined for email and postal transmission.

#### Legitimacy status investigation

Relevant information regarding the samples, including the manufacturer names and their licence numbers, trade/product names, batch numbers, APIs, strengths and dosage formulations, was sent to the DDA, which checked the legitimacy status of the samples using the latest available product and manufacturer authorisation lists.

### Chemical analysis

The chemical analysis was conducted in the analytical laboratory of Kanazawa University in Japan. All applicable samples underwent the dissolution test ‘ < 711 > ’, the uniformity of dosage units test, the assessment of content uniformity ‘ < 905 > ’, and the assay test as per the USP 41 instructions and acceptance criteria^47^. Assay test values were calculated as an average of 10 individual unit contents which were tested in the first stage of the uniformity of dosage units test ‘ < 905 > ’^47^. However, for samples which required second-stage testing, the assay value was calculated from the total 30 individual contents tested (first- and second stage).

A summary of the HPLC methods is provided in Table 1, a summary of the dissolution test conditions is given in Table 2. However, the detailed HPLC and chemical analysis methods are fully described in Supplementary Material 1. The individual acceptance criteria which corresponded to USP 41 criteria for each of the four APIs^47^ are provided in Supplementary Material 2. In this study, samples which failed quality testing in an intermediate stage but did not have sufficient units to finalise quality testing (hereinafter referred to as ‘interim failed’ or ‘interim failing’ samples) were judged as ‘passed’.

**Table 2:** Overview of the dissolution test conditions.

API	Apparatus type	Medium	Rotation speed	Temperature	Testing time
AZM	Apparatus 2: Paddle	pH 6.0 buffer	75 rpm	37.0 °C ± 0.5 °C	30 min
CFIX	Apparatus 2: Paddle	pH 7.2 buffer	100 rpm	37.0 °C ± 0.5 °C	45 min
ESM	Apparatus 2: Paddle	Acid stage: 0.1 M HCl Buffer stage: pH 6.8 buffer	100 rpm	37.0 °C ± 0.5 °C	Acid stage: 120 min Buffer stage: 30 min
LST	Apparatus 2: Paddle	Distilled, deaerated water	50 rpm	37.0 °C ± 0.5 °C	30 min

Calibration curve and quality control (QC) solutions were freshly prepared for each HPLC run, and all other solutions used in analysis preparation, such as buffer solutions, were freshly prepared for each analysis day. QC solutions of three concentrations were prepared for the analysis of AZM, ESM and LST. For CFIX, QC solutions were prepared only during validation.

The method validation followed general Good Laboratory Practice guidelines^48^ and the ‘The International Council for Harmonisation of Technical Requirements for Pharmaceuticals for Human Use’ Q2(R2) guideline^49^. AZM, CFIX, ESM and LST standard products approved in Japan (see Chemicals and Materials section) were analysed before sample analysis. A detailed overview of the validation results is given in Supplementary Material 4.

The peak data for API and internal standards were calculated by the respective software programmes specified in the Devices and Instrumentation section, and the data were exported to Excel MSO 365 (Microsoft Corp., Redmond, WA, USA).

Images of failing units were taken after the acid stage (only for ESM samples) and after the buffer stage of the dissolution tests.

### Price analysis

During the sampling process, the individual sample prices which were paid by samplers were documented in the sampling form. However, the samplers did not collect the individual payment receipts. Prices which were indicated in Indian rupees (INR) were converted into Nepalese rupees (NPR) using the rounded INR-to-NPR exchange rate of April 27, 2022 (INR1 = NPR1.6)^50^. Subsequently, all prices were converted into United States dollars (USD) using the NPR-to-USD exchange rate of April 27, 2022 (USD1 = NPR122.5964)^51^, the date when sampling started in the Saptari district. The unit prices of the samples were plotted according to the results of the quality analysis. The median price per unit was determined for each API of the collected samples. The median medicine price ratios (MPRs), 25th percentile MPR, 75th percentile MPR, minimum MPR and maximum MPR in the absolute median and the 25th and 75th percentile, minimum, and maximum prices per unit were divided by the international reference unit price (IRP). The IRPs were taken from the reference unit price list of the supplier median prices published by the Management Sciences for Health and the WHO in 2016^52,53^.

### Small-scale dissolution test evaluation

To evaluate the applicability of the small-scale dissolution test previously proposed by Rahman and Yoshida et al.^35^ as a screening technology, the small-scale dissolution test criteria were applied to this study. The screening technology uses various compliance criteria to evaluate dissolution test results using *n* = 3 units which are tested for dissolution (instead of the 6–24 units required for the USP dissolution test). The following compliance criteria resulted for the four APIs: the AZM and CFIX dissolution test results averaged Q ≥ 86%, with all individual Qs ≥ 82%, and the ESM and LST dissolution test results averaged Q ≥ 81%, with all individual Qs ≥ 77%.

These small-scale dissolution test compliance criteria were then applied to all.

$\left(\genfrac{}{}{0pt}{}{n}{k}\right)=\frac{n!}{k!*\left(n-k\right)!}=\left(\genfrac{}{}{0pt}{}{6}{3}\right)=20$ possible *n* = 3 combinations of the* n* = 6 individual Q results obtained in the first stage of the dissolution test using the methods described in the Chemical Analysis section.

The evaluation was expressed as the compliance of the small-scale dissolution test result of each *n* = 3 combination with the final test results of the USP 41 dissolution test. The results were compliant if test results were either both ‘pass’ or both ‘fail’, they were false negative (= falsely non-compliant) if the small-scale dissolution test result was ‘fail’ but the USP 41 dissolution test was ‘pass’, and they were false positive (= true non-compliant) if the small-scale dissolution test was ‘pass’ and the USP 41 dissolution test was ‘fail’. Importantly, the small-scale dissolution test results were not compared with the USP 41 first-stage test result but to the last (= final) tested stage result if further test stages were conducted.

The compliance of both test results was expressed as the overall percentage of samples in agreement. The rate of false-negative judgement was expressed as the overall percentage of samples which required retesting and the individual rate for each of the 20 combinations per *n* = 6 Q results which required retesting despite an expected ‘pass’ for the sample. The rate of false-positive acceptance of the samples was expressed as an overall percentage, a total percentage of all individual combinations per *n* = 6 Q results and the individual rate for each of the 20 combinations per *n* = 6 Q results of samples which were false-positively accepted in the small-scale dissolution test.

### Raman scattering analysis

Before analysis, the supplied software was installed on a personal computer and the C13560 and Inspector500 portable Raman spectrometers were set up in accordance with manufacturer instructions.

Raman scattering analysis was conducted in accordance with the methods applied by Schreiber et al.^41^. A summary of the Raman spectroscopy conditions is given in Table 3. For the C13560 device, the output was set at 15 mW (‘High’), the excitation laser wavelength was set at 785 nm, the scanning time was 1000 ms per scan and the spectral X-axis wavenumber interval was set between 98 and 1852 cm^−1^. Before taking the measurements, the dark signal was measured by inserting the silicon substrate provided by the manufacturer. Subsequent calibration of the software was performed using the Raman shift peak of the substrate near 521 cm^−1^. As per the instructions, the emitted laser wavelength was reset manually to 785 nm immediately after calibration.

**Table 3:** Overview of the Raman spectroscopy testing conditions.

Raman spectrometer	Laser wavelength	Spectral resolution	Scan time	Spectral data points per spectrum	Output
C13560 device	785 nm	10 cm^−1^	1000 ms	50 spectral data points averaged	15 mW
Inspector500	1030 nm	8–10 cm^−1^	Automatic (max. 8000 ms)	50 spectral data points averaged	300 mW

N/A = not available.

For the Inspector500, the output was set at 300 mW (‘High’), the excitation laser wavelength was set at 1030 nm, the scanning time was set to the default ‘automatic’ setup with a maximum of 8 s and the spectral X-axis wavenumber interval was between 150 and 2450 cm^−1^. Before measurements were taken, a calibration test was conducted in accordance with the instructions. If the test failed, the device was recalibrated repeatedly until it passed the test.

The measurement procedure was identical for both devices: the tablets were placed in front of the laser source. Measurements were taken by placing the spectrometer’s attachment for analysis in direct contact with each tablet to completely cover the tablet surface to ensure that the laser directly hit the tablet. During analysis, a black opaque fabric was used to cover the set-up and prevent light contamination. One measurement was obtained as an average of five spectral data points from the Raman scattering analysis. A total of 10 measurements per tablet were performed on randomly chosen spots of the sample surface. Five measurements were taken from each of the top and bottom of the tablet. Therefore, a total of 50 spectral data points were averaged, resulting in one spectrum for each sample for each device and creating a spectral library for each of the products. Capsules were opened and the granules were placed in a small plastic film bag before being analysed in the same way as for the tablets.

For the C13560, the Spectral X-axis wavenumber interval was set between 403 and 1852 cm^−1^. Raman spectra were line plotted and colourised using the Unscrambler X 10.5 (CAMO Software AS, Oslo, Norway).

### Statistical analysis

Descriptive statistical analysis including the calculation of means, standard deviations (SDs) and relative standard deviations (%RSDs) was performed using Excel MSO 365 Versions 2307–2404 and 2506 (Microsoft Corp., Redmond, WA, USA). Fisher’s exact test (any cell value of the contingency table < 5) and Pearson’s chi-squared test (all cell values of the contingency table ≥ 5) were used where appropriate; these tests were performed using SPSS version 29.0.2.0 (IBM Corporation, Armonk, NY, USA). Statistical significance was assumed at the *p* < 5% level for all tests.

## Results

### Sampling

#### Sample collection

A total of 241 samples were collected from 91 sampling sites in Nepal. Of the total, 121 samples were acquired from April 27 to May 6, 2022, in the Saptari district (31 sampling sites) and 120 samples were collected from May 14 to June 8, 2022, in the Kathmandu district (60 sampling sites). Although the samplers did not present prescriptions, none of the samples were refused to them during sampling.

The 241 samples consisted of a total of 59 products and trade names from 113 different batches produced by 36 manufacturers based in two countries. According to the sample labelling, 173 samples originated in Nepal (71.8%) and 68 in India (28.2%). A total of 138 samples (57.3%) were sampled as blisters only and had no secondary packaging.

A comprehensive, anonymised overview of samples is presented in Supplementary Material 3 (Supplementary Tables S1a–b, S2a–d, S3 and S4).

#### Sampling sites

Out of the 91 sampling sites, 88 sites (96.7%) were confirmed as licenced vendors: 87 sampling sites (95.6%) were categorised as legal pharmacies and one site (1.1%) was a legal wholesaler. The shop category of the three sampling sites was not specified by the samplers, but the eight samples acquired from these sampling sites were included in the study. Sampling sites IDs 1–31 were located in the Saptari district, whereas sampling sites IDs 32–91 were located in the Kathmandu district. Of the 31 sampling sites in the Saptari district, a total of 30 site locations (*n* = 5) or areas (*n* = 25) could be identified and were mapped (Fig. 1). In the Kathmandu district, all 60 sampling sites were identified and mapped either as locations if available or as areas; among the sites, 41 (68.3%) were documented or identified as sampling site locations and 19 (31.7%) were documented as sampling site areas (Fig. 2).

Sixty-five of the 91 sampling sites (71.4%; 95% CI 61.0–80.4) had indoor temperatures above the 2022 recommended 25 °C, with a maximum temperature reaching 30 °C in five sampling sites. Additionally, the relative humidity level of one sampling site (1.1%; 95% CI 0.0–6.0), reached 75%. No sampling site exceeded the stability testing conditions of pharmaceutical climatic zone IVb.

A comprehensive, anonymised overview of sampling sites is presented in Supplementary Material 3 (Supplementary Tables S1b and S5a–c).

### Visual observation, authenticity investigation and legitimacy status investigation

#### Visual observation

**Fig. 3:**
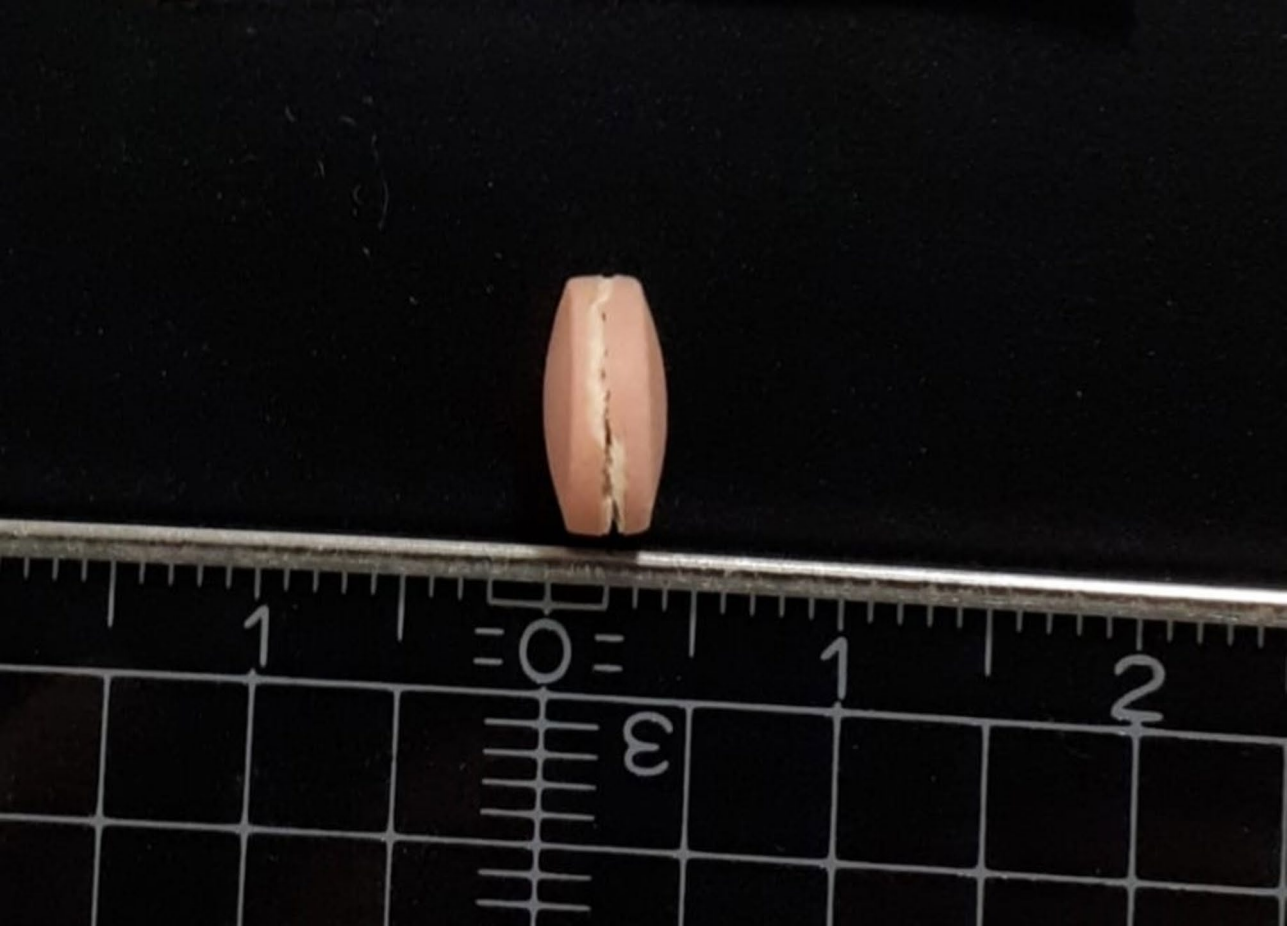
Side view of the cracked and damaged tablet of sample B-110 which was separated before analysis of the sample. Shows the image of a unit of sample B-110. The unit exhibits severe cracks and damages in the enteric coating and the tablet core. The markings on the ruler indicate the distance in cm. Intermediate markings are in mm.

Of the 241 collected samples, a total of 16 samples (6.6%; 95% CI 3.8–10.6) failed the visual observation test. Specifically, the dosage units of all of the 16 failing samples (AZM: 0, CFIX: 0, ESM: 12, and LST: 4) showed cracks (A-103, A-106, A-108, B-101, B-105, B-110, C-207 and D-211), broken additional tablet pieces within the blisters (C-211), or inhomogeneous coatings (B-115-2, C-103, C-110, C-113, D-103, D-113 and D-205). One tablet of sample B-110 (shown in Fig. 3) had severe cracks and was separated before chemical analysis; the tablet was used for further investigations, which are not discussed in this study. The separation of one unit of sample B-110 likely biased the SF medicine occurrence estimation of this study. Overall, only three samples (1.2%; C-106, D-314 and D-404) contained a leaflet, which was available only in English.

#### Authenticity investigation

A total of 16 of the 36 manufacturers (44.4%; 95% CI 27.9–61.9) replied with completed authenticity forms. Twelve manufacturers (75%) were based in Nepal and four (25%) in India (Supplementary Tables 1a–b), but the difference was not significant: Fisher’s exact test; 2-Tail (1, *n* = 36) = 0.9, *p* = 0.343; *p* > 0.05. Emailed forms led to six completed authenticity forms, and posted forms led to an additional ten replies, an increase from 16.7% to 44.4%. Thirty manufacturers (83.3%) were classified as both non-responder type 1 and non-responder type 2 before the authenticity investigation forms were sent by post, and 20 manufacturers (55.6%) were classified as non-responder type 3 after forms were sent by post. In the authenticity investigation, the 16 manufacturers who responded declared 118 samples (49.0%) as genuine and no sample was suspected by the manufacturers of being falsified in their responses, whereas no response was received for the remaining 123 samples (51.0%).

#### Authenticity investigation response time analysis

The median response time for six replies from manufacturers following emailing using the sending date as the dispatch date was 7.5 days (mean = 7.2 days, SD = 4.0 days), with minimum and maximum response times of two and 14 days, respectively.

For forms sent by post, the median response time for 10 replies from manufacturers was 23.5 days (arithmetic mean = 25.6 days, SD = 10.2 days), with minimum and maximum response times of 14 and 47 days, respectively, including an unknown travel time for each sent package. Determining the exact arrival date for forms sent by post and mail delivery times was not possible; therefore, all response times for postal delivery included individual mail delivery times.

#### Legitimacy status investigation

The DDA stated that the indicated information regarding manufacturer name and licence number, product name, batch number, API, strength, and dosage formulation of all 241 collected samples were legitimate and that the medications were authorised for distribution in Nepal.

### Chemical analysis

#### Method validation

The validation parameters for each API (AZM, CFIX, ESM and LST) and the test results for the four standard products are summarised in Supplementary Material 4 (Supplementary Tables S6a–d and S7).

#### Summary of the chemical analysis results

A total of 24 of the 241 samples (9.96%; 95% CI 6.5–14.5) failed one or more of the pharmacopoeial quality tests (AZM: 1, CFIX: 3, ESM: 7 and LST: 13). One AZM sample (0.41%) interim failed quality testing without sufficient sample material to continue. The API identity was confirmed in all 241 samples, and no additional APIs were detected with the analytical methods used. However, 28 samples (CFIX: 22 and ESM: 6) could not undergo dissolution testing because USP 41 evaluation criteria were not defined for their formulations (uncoated dispersible tablets and film-coated tablets). Therefore, 213 samples (88.4%; AZM: 60, CFIX: 38, ESM: 55 and LST: 60) were tested for dissolution.

Table 4 provides an overview of the quality analysis test results. The individual chemical analysis results of the pharmacopoeial tests are shown and described in detail in Supplementary Material 5 (Supplementary Tables S8-S10, S11a–d, S12a–b, S13a–b, S14a–b and S15a–b).

**Table 4:** Overview of the quality analysis results.

API	Samples tested	Pass	Fail	
			Interim	Final
Azithromycin	60	58	1	1
Cefixime	60	57	0	3
Esomeprazole	61	54	0	7
Losartan	60	47	0	13
Total	241	216	1	24

Uniformity of Dosage Units Test:

Three of the 241 tested samples (1.2%; 95% CI 0.3–3.6) failed the uniformity of dosage units test, and another three samples (1.2%) interim failed the first stage of this test. However, insufficient units were left to continue the test.

Assay Test:

Overall, six of the 241 samples (2.5%; 95% CI 0.9–5.3) failed the assay test; however, none of the samples had an average API content below 80%, a value which can be considered a ‘moderate deviation’.

**Fig. 4:**
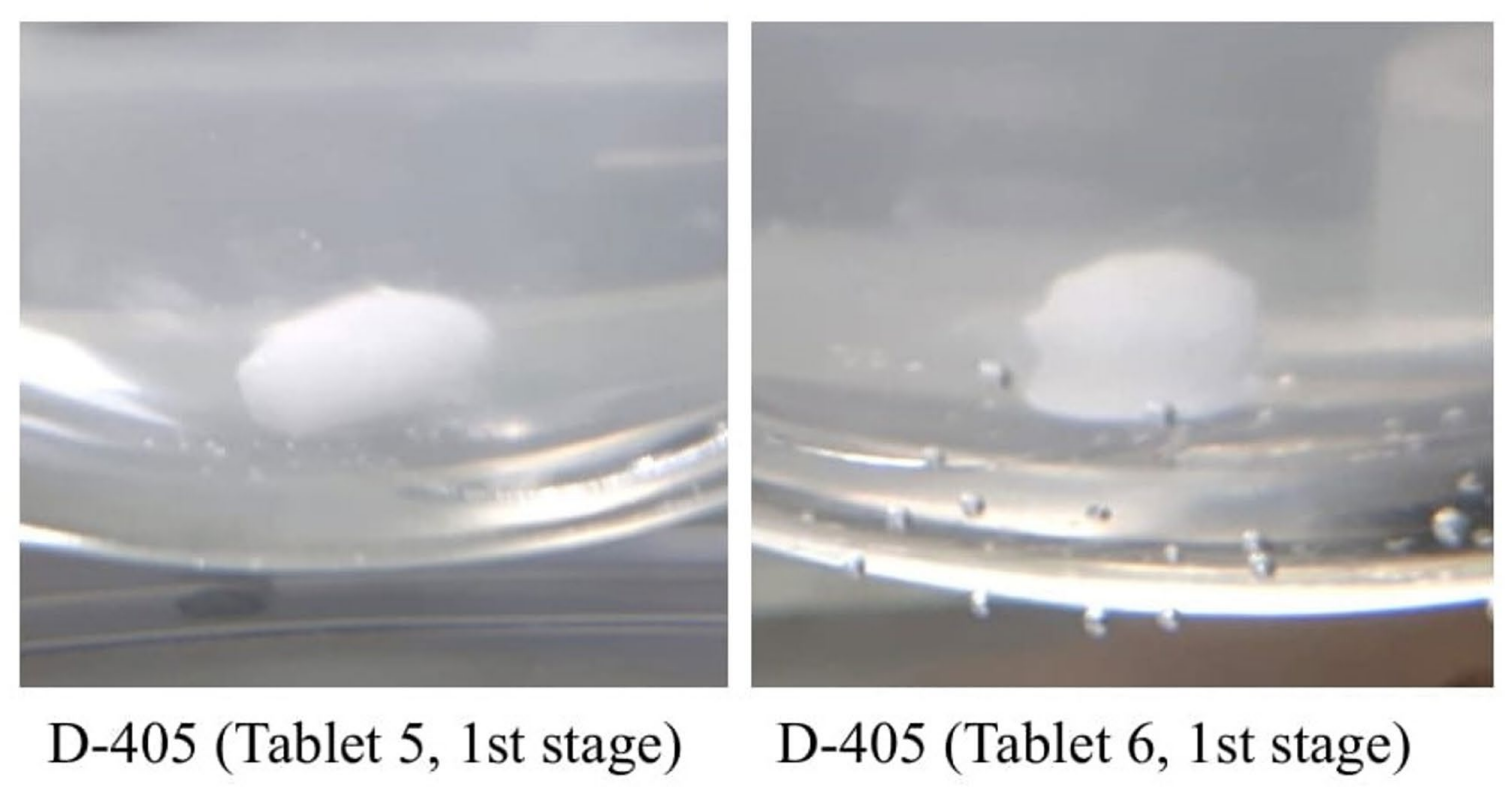
Images of representative units of AZM sample D-405 taken immediately after the dissolution test. Shows images of representative units of AZM sample D-405 (units 5 and 6), which did not pass the USP dissolution test. The tablet core remains which did not sufficiently dissolve are distinctly visible. The images were taken immediately after the sample solution was drawn from the dissolution tester.

**Fig. 5:**
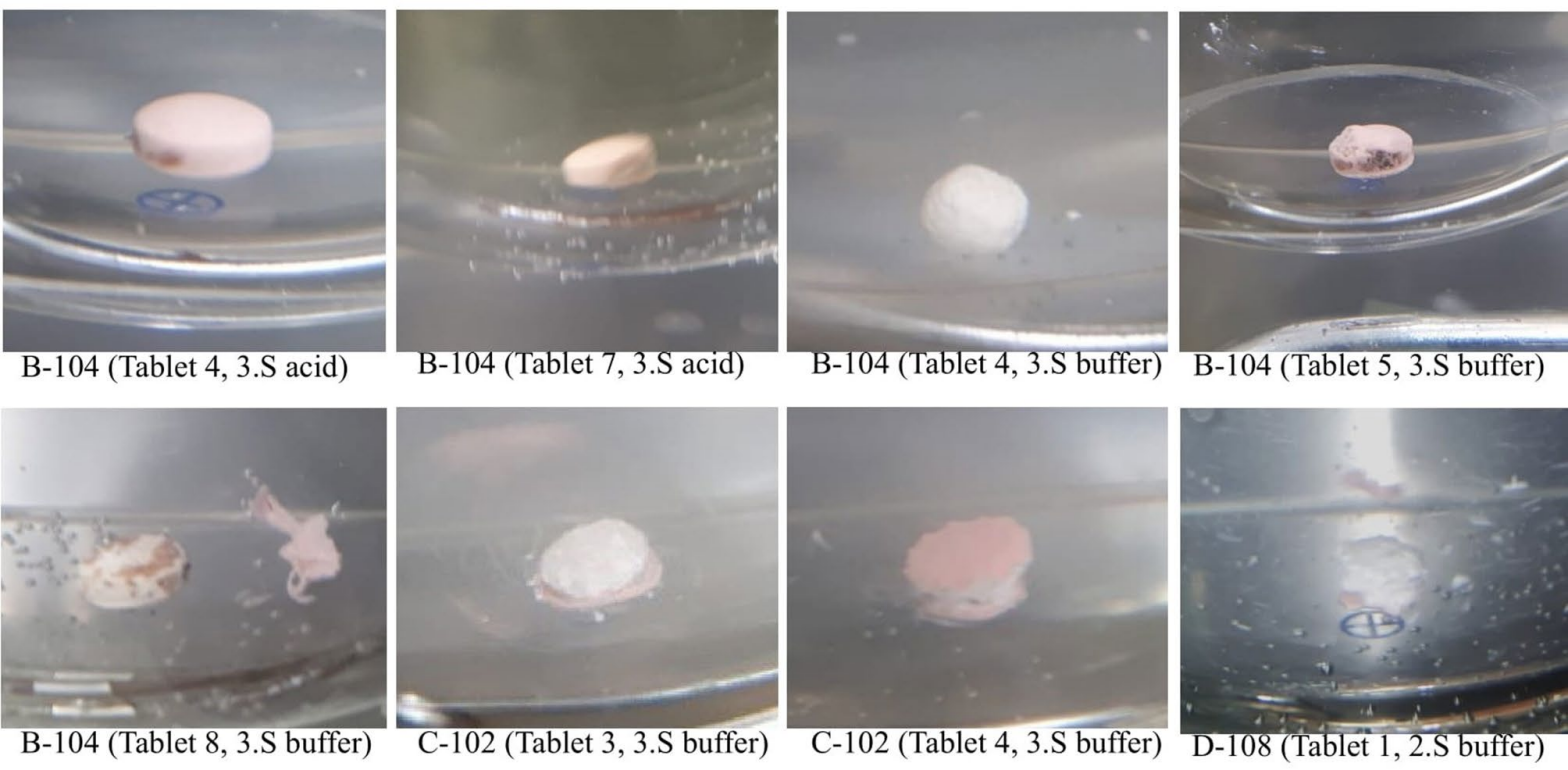
Images of representative ESM units of failing samples B-104, C-102 and D-108 taken immediately after the dissolution test acid or buffer stages. Shows images of representative units of ESM samples B-104, C-102 and D-108, which did not pass the USP dissolution test. The images were taken immediately after the sample solution was drawn from the dissolution tester for both the acid and buffer stages. Image taking for sample A-105 was missed. 3.S acid = third stage of the acid stage. 2.S buffer = second stage of the buffer stage. 3.S buffer = third stage of the buffer stage.

**Fig. 6:**
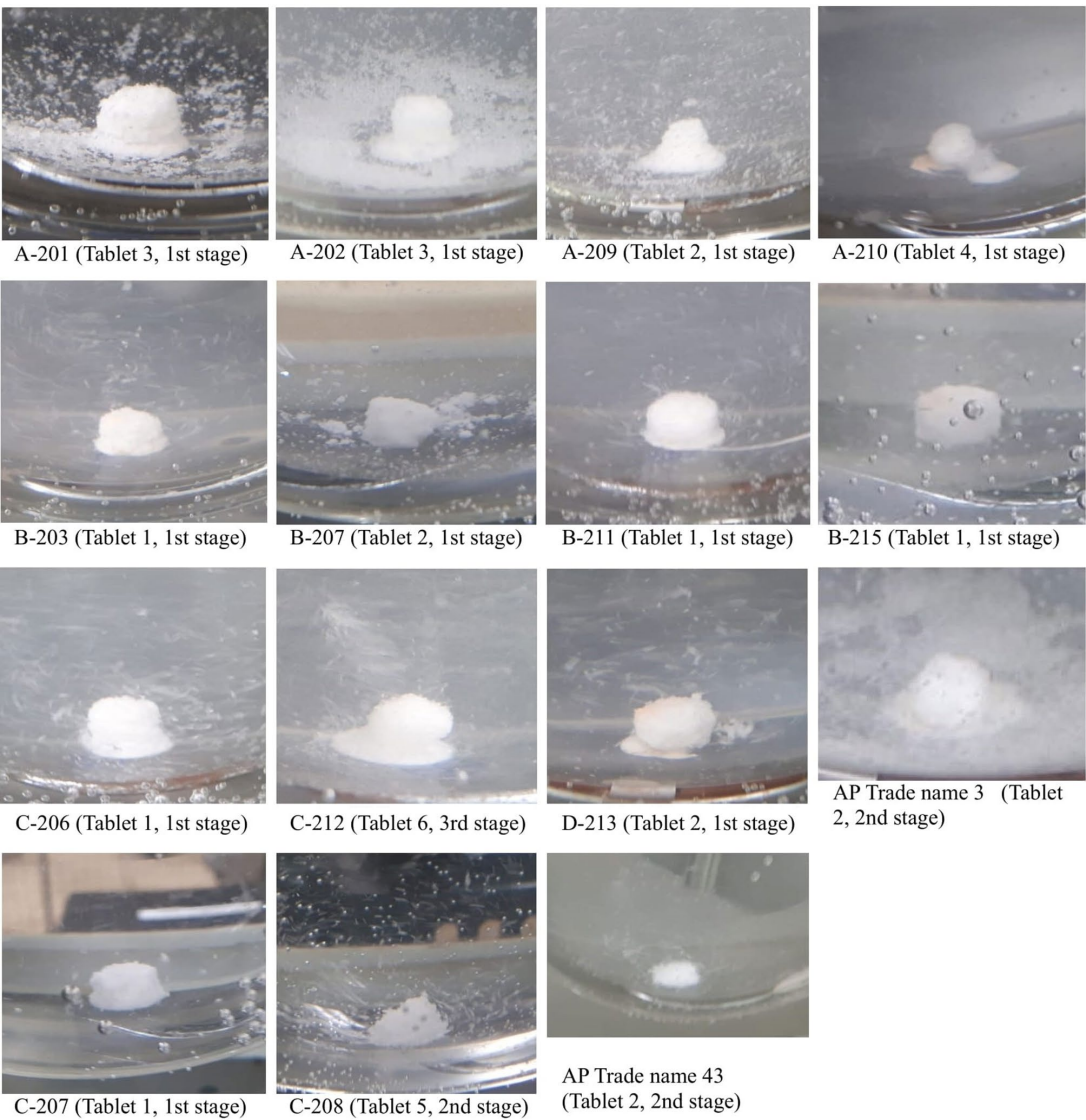
Images of representative LST units of failing samples immediately after the dissolution test. Shows images of representative units of LST samples A-201, A-210, B-203, B-207, B-211, B-215, C-206, D-213, AP Trade name 3, C-207, C-208, and AP Trade name 43, which did not pass the USP dissolution test. The images were taken immediately after the sample solution was drawn from the dissolution tester, but image-taking for sample A-213 was missed. Sample C-208 and AP Trade name 43 passed the USP dissolution test and were compared with C-207, which was labelled the same as Trade name 43 but presented a different batch number.

Dissolution Test:

Eighteen of the 213 tested samples (8.5%; 95% CI 5.1–13.0) failed the dissolution test. Figures 4, 5 and 6 provide images of representative failing units per sample that were taken immediately after drawing the sample solution.

#### Overview of test-failing samples

Twenty-four samples of the four APIs (AZM, CFIX, ESM and LST) which represented nine of the 59 products (15.3%; 95% CI 7.2–27.0) and 13 of the 113 batches (11.5%; 95% CI 6.3–18.9) manufactured by 9 of the 36 manufacturers (25.0%) failed at least one test, as summarised in Supplementary Tables S11a–d. Representative HPLC chromatograms are presented in Supplementary Material 6 (Supplementary Figs. S1–S4). Twenty (83.3% of the total) and four test-failing samples (16.7% of the total) were labelled as having been manufactured in Nepal (out of 173) and India (out of 68), respectively. However, the difference between the origin of manufacture was insignificant: Fisher’s exact test; 2-tail (1, *n* = 241) = 1.76, *p* = 0.185; *p* > 0.05. Among the 118 authenticated samples (see Visual Observation, Authenticity Investigation and Legitimacy Status Investigation section), 16 samples (13.6%) of three products failed quality testing, whereas eight samples (6.5%) of the 123 samples for which no reply was received failed quality testing. However, the observed difference between the groups was insignificant: Pearson’s chi-squared test; (1, *n* = 241) = 3.34, *p* = 0.343; *p* > 0.05.

The average proportion of test-failing antibiotics (3.33%; 95% CI 0.9–8.3; 4 of 120 AZM and CFIX samples) was lower than that of medicines against non-communicable disease (NCDs; 16.5%; 95% CI 10.4–24.4; 20 of 121 ESM and LST samples) and the difference was significant: Fisher’s exact test; 2-tail (1, *n* = 241) = 11.7, *p* = 0.001; *p* < 0.05. Furthermore, the average proportion of test-failing samples collected in the Saptari district (13.22%; 95% CI 7.8–20.6; 16of 121 samples) was higher than that of those collected in the Kathmandu district (6.67%; 95% CI 2.9–12.7; 8 out of 120 samples). However, no significant difference was observed: Pearson’s chi-squared test; (1, *n* = 241) = 2.89, *p* = 0.09; *p* > 0.05.

### Price analysis

Price documentation was missing for one CFIX sample (B-313) and three LST samples (B-201, C-211, C-213), all of which passed the performed quality tests.

**Fig. 7:**
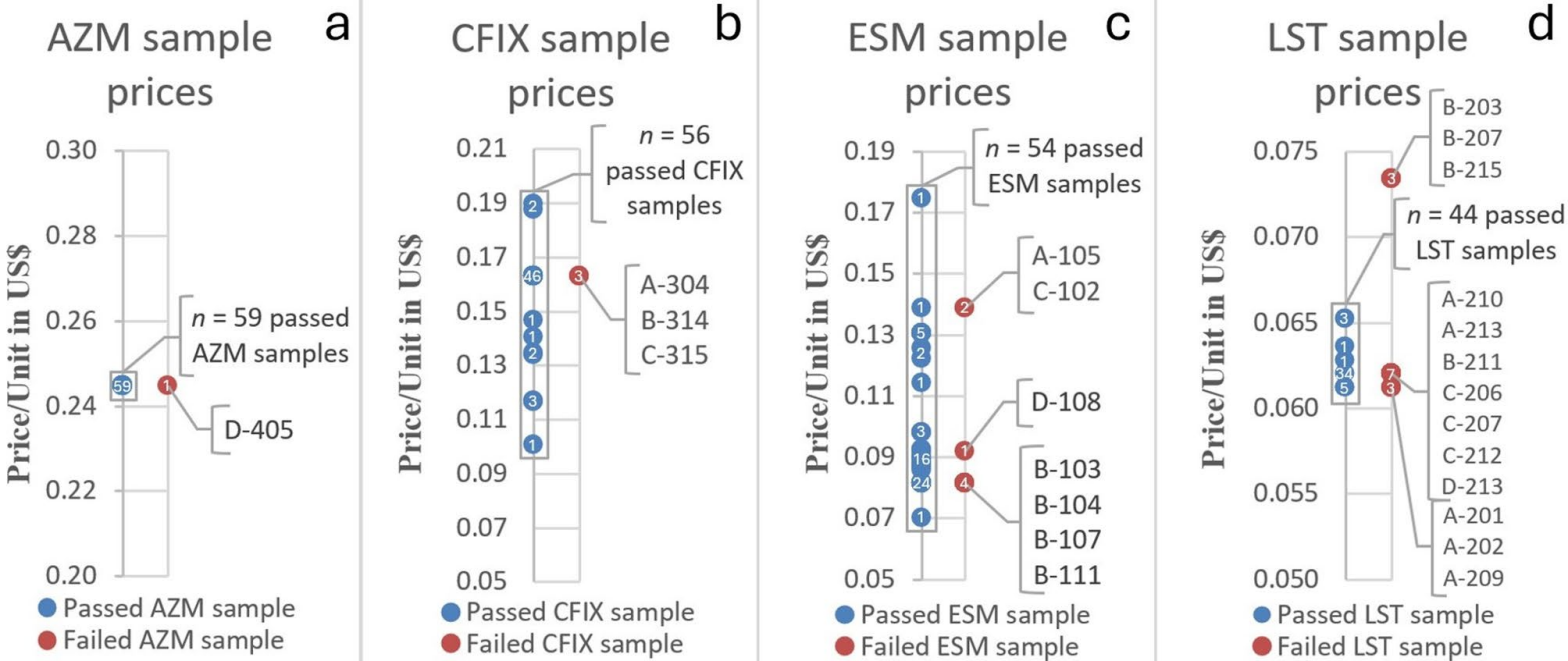
a–d: Unit prices of AZM, CFIX, ESM and LST samples in US dollars, categorised by quality analysis results. (**a**)–(**d**) shows the available unit prices of the AZM (**a**), CFIX (**b**), ESM (**c**) and LST (**d**) samples in US dollars per unit. Blue dots represent samples that passed quality tests, whereas red circles indicate samples that failed at least one pharmacopoeial test. Each subplot includes a white number indicating the count of samples with a similar or identical unit price. Unit prices for four samples (B-313, B-201, C-211 and C-213) were unavailable and are therefore not included in the figure.

**Fig. 8:**
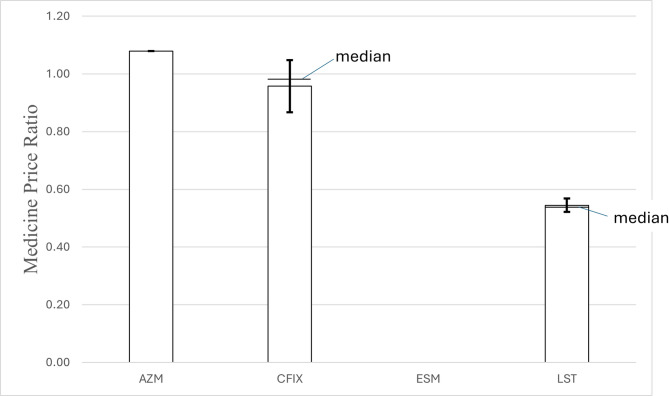
Medicine Price Ratios (MPRs) of AZM, CFIX and LST samples. Illustrates the median MPRs of AZM, CFIX and LST including the standard deviation (SD). All 60 AZM samples were documented to be sold at an MPR of 1.08 (mean: 1.08, SD: 0.00), the median MPR of CFIX was 0.98 (mean: 0.96, SD: 0.09), and the median MPR of LST was 0.54 (mean: 0.54, SD: 0.02). The international reference unit price of ESM was not available, therefore the MPR could not be calculated.

The unit prices of the samples were plotted according to the results of the quality analysis (see Fig. 7a–d). The rounded median prices per unit for each of the four APIs were USD 0.245 (arithmetic mean = USD 0.245; %RSD = 0.0%) for AZM, USD 0.163 (arithmetic mean = USD 0.159; %RSD = 9.4%) for CFIX, USD 0.086 (mean = USD 0.095; %RSD = 22.1%) for ESM and USD 0.062 (mean = USD 0.063; %RSD = 4.3%) for LST. IRPs were only available for omeprazole 20 mg tablets and capsules and 40 mg injection vials but not for ESM. Thus, the MPRs of ESM could not be assessed. According to the documentation, all of the 60 AZM samples were sold at the same MPR of 1.08 (SD = 0.00). The median MPR of CFIX was 0.98 (SD = 0.09), with minimum and maximum MPRs of 0.61 and 1.14, respectively. The median MPR of LST was 0.54 (SD = 0.02), with minimum and maximum MPRs of 0.53 and 0.64, respectively. The MPRs for the individual APIs are shown in Fig. 8. A comprehensive overview of the individual price data is provided in Supplementary Material 7 (Supplementary Figs. S5–S7).

For AZM, the median and average prices per unit were identical between failed and passed samples (USD 0.245). Similarly, for CFIX, the median unit price was the same for both groups (USD 0.163), although the average price was slightly higher among failed samples (USD 0.163 vs. USD 0.159). For ESM, failed samples had a lower median unit price (USD 0.082 vs. USD 0.087) but a slightly higher average unit price (USD 0.099 vs. USD 0.094) compared to passed samples. In the case of LST, the median unit price was equal between both groups (USD 0.062), while the average unit price was marginally higher for failed samples (USD 0.064 vs. 0.062).

### Small-scale dissolution test evaluation

The small-scale dissolution test criteria (Ø dissolution rate of the sample ≥ Q_USP_ + 6%; the three individual dissolution rates ≥ Q_USP_ + 2%) were applied to all of the 20 combinations of *n* = 3 units for each of the* n* = 6 dissolution rates per sample measured in the first stage of the USP dissolution test, resulting in 4,260 results for all possible *n* = 3 combinations.

Of the 213 tested samples, agreement was observed between small-scale and USP dissolution test results in all of the 20 *n* = 3 combinations of 192 samples (90.1%), which were in all four APIs. Overall, 245 *n* = 3 combinations (AZM: 183 CFIX: 0 ESM: 21, LST: 41) of the total 4,260 *n* = 3 combinations (5.75%; 95% CI 4.8–6.5) were false negatives and 39 *n* = 3 combinations (AZM: 1 CFIX: 0 ESM: 38, LST: 0) of the total 4,260 *n* = 3 combinations (0.92%; 95% CI 0.7–1.3) were false positives when compared with the last-tested stage USP dissolution test results (AZM: 0.1% false-positive [0.08%; 95% CI 0.0–0.5; 1 of 60 samples; 1 of 1,200 three-unit combination], 15% false-negative [15.3%; 95% CI 13.3–17.4; 10 of 60 samples, 183 of 1,200 three-unit combinations]; CFIX: 0 of 38 samples false-positive or false-negative; ESM: 3% false-positive [3.5%; 95% CI 2.5–4.7; 4 of 55 samples; 38 of 1,100 three-unit combinations], 2% false-negative [1.9%; 95% CI 1.2–2.9; 3 of 55 samples, 21 of 1,100 three-unit combinations]; LST: 0 false-positive, 3% false-negative [3.4%; 95% CI 2.5–4.6; 3 of 60 samples, 41 of 1,200 three-unit combinations]).

### Raman scattering analysis

Raman spectra of all 241 samples and the reference standards for AZM, CFIX, omeprazole and LST) were obtained using both portable Raman spectrometers. For AZM, CFIX and LST samples, 172 of 180 (95.6%; 95% CI 91.4–98.1) spectra collected with the C13560 and all 180 of the spectra collected with the Inspector500 revealed characteristic peaks that corresponded to the respective reference standard. However, the visible AZM Raman peaks observed with the C13560 were small and thus difficult to identify. No characteristic peak was observed for the ESM samples when these were visually compared with the omeprazole reference standard. The spectra are presented in Supplementary Material 8 (Supplementary Figs. S8–S15).

Notably, the detection limits for API quantities across different formulations were not assessed, and spectral comparisons of passing and failing samples were not successful in predicting sample quality given that their spectra were often too similar. Variations in spectral profiles appeared to be influenced more by surface colour differences than by differences in sample quality, e.g. LST product Trade name 3.

## Discussion

In this study, 241 samples of four APIs–AZM, CFIX, ESM and LST – were collected from 91 licenced vendors in the Saptari and Kathmandu districts of Nepal using randomly conducted convenience sampling (Saptari district) and the randomised selection of sampling sites (Kathmandu district; Figs. 1 and 2; Supplementary Table S1a–b; Supplementary Figs. S1 and S2). A total of 24 samples (10.0%; 95% CI 6.5–14.5) failed at least one of the three pharmacopoeial quality tests conducted (assay, uniformity of dosage units and dissolution; Table 4; Figs. 4, 5 and 6; Supplementary Tables S11a–d, S12a–b, S13a–b, S14a–b and S15a–b). This result was in line with the WHO’s global estimate of 10.5% SF medicines in LMICs^8^ and a recent systematic review by Ozawa et al. which estimated a 10.2% prevalence of SF medicines in Asian LMICs^17^; the 95% confidence interval of the current study (6.5–14.5%) overlaps with both reference estimates.

### Classification of the pharmacopoeial test-failing samples

Of the 24 samples that failed pharmacopoeial testing (= ‘test-failing’ or ‘SF’ samples), 16 authenticated samples were classified as ‘substandard’ based on chemical analysis and confirmation of authenticity by the manufacturers (B-103, B-104, B-107, B-111, A-201, A-202, A-209, A-210, A-213, B-203, B-207, B-211, B-215, C-206, C-212, D-213). The eight samples for which no response was received from the respective manufacturers (A-105, C-102, D-108, C-207, A-304, B-314, C-315, D-405) were classified as ‘probably substandard’ according to the approach proposed by Ozawa et al.^17^, as they had failed pharmacopoeial testing but had average API contents above 80% of the declared amount, and no evidence of falsification and/or criminal intent was identified in this study. For simplicity, ‘probably substandard’ samples are referred to as ‘substandard’ hereinafter. However, although no indications of falsification were found for any sample, the classifications of the eight ‘probably substandard’ samples remain uncertain—especially given the 56% non-response rate—, and this limitation must be acknowledged.

### Implications of the occurrence of SF samples in Nepal

Among the 24 substandard samples, medicines against NCDs, i.e. ESM and LST, included a significantly higher proportion of substandard medicines than antibiotics, i.e. AZM and CFIX, (20 samples [8.3%] versus 4 samples [1.7%]; Fisher’s exact test; 2-Tail; *p* < 0.05). Given that antibiotics have been commonly reported as SF medicines^8,11,17,54^ and NCDs cause a high burden of disease in Nepal and globally^55,56^, this finding is noteworthy and suggests that future medicine quality studies should continue to elucidate the quality of medicines against NCDs. The result further indicates that poor-quality medicines against NCDs may present a potential contributing factor to the burden of disease in Nepal. Nonetheless, substandard antibiotics were also identified in this study. SF antibiotics can impact treatment and potentially contribute to antimicrobial resistance given their poor dissolution (D-405; Fig. 4, Supplementary Table S12a) and slightly lower average content or non-uniform content in dosage units (A-304, B-314 and C-315; Supplementary Table S13b)^33,57–59^.

Overall, the findings of this study are unexpectedly moderate, considering that Nepal’s regulatory system is classified at WHO maturity level 1. This classification reflects limited regulatory capacity, which may include, for example, inadequate oversight of manufacturing standards due to infrequent inspections of industry and laboratories, limited post-market surveillance, and insufficient or delayed mechanisms for identification of SF medicines or preventing their dissemination in practice^26,27^. Despite concerns expressed by Nepalese healthcare professionals who recently described Nepal as vulnerable to an influx of falsified medicines^60^, no evidence of falsification was found for any sample in this study. This finding is consistent with previous reports from Nepal^61–64^ and the DDA’s statement that no official or unofficial reports on falsified medicines have been issued in Nepal. However, the absence of detected falsified medicines in this study may be attributable to methodological and sampling limitations. Accordingly, the possibility of their presence in Nepal cannot be conclusively dismissed.

Previously, four studies reported that nine of 214 samples (4.2%; 95% CI 1.9–7.8)^62^, nine of 172 samples (5.2%; 2.4–9.7)^63^, 13 of 40 samples (32.5%; 95% CI 18.6–49.1)^64^ and 37 of 244 samples (15.2%; 95% CI 10.9–20.3)^61^ were SF medicines. All reported test-failing samples had specifically been classified as ‘substandard’, and no study had reported any falsified medicines. Therefore, the data may suggest that falsified medicines could be less prevalent in Nepal—and potentially in the entire geographical region—than the global average data for LMICs or the OECD data would indicate^17,22^. It is important to note that the study reporting the alarmingly high failure rate of 32.5% was based on a small, non-representative sample size and reported discrepancies in analytical test results between two laboratories. The studies reporting 4.2% and 5.2% ‘substandard medicines’ did not provide information on randomisation or sampling methodologies; in the latter case, the specific medicines sampled were not reported. Therefore, their comparability to Dhakal et al.’s study, which employed randomised multi-district sampling in public health care facilities, and the present study is limited. Although the average test-failing samples were different between Dhakal et al.’s study and the present study (15%; 95% CI 10.9–20.3 vs. 10%; 95% CI 6.5–14.4), this difference was not significant: Pearson’s chi-squared test; (1, *n* = 485) = 2.99, *p* = 0.084; *p* > 0.05. This may imply that the quality of the distributed medicines could be comparable throughout the country, but the underlying data for this, such as analysed APIs and sample sizes, remain limited.

### Assessment of the visual observation test results

Only one of the 16 samples (6.25%) that failed the visual observation test also failed the pharmacopoeial analysis result (C-207), which exhibited noticeable porosity. This underscores the critical importance of full laboratory analysis for assessing medicine quality, while also demonstrating the benefits of visual observation in identifying poorly manufactured or mechanically compromised units; and, by extension, suggesting potential manufacturing flaws. Furthermore, the low number of collected leaflets indicates that many medicines from licenced vendors in Nepal are distributed to consumers without accompanying written information, i.e. instructions for use or health information, thus potentially leading to usage errors such as unintentional misapplication or dosing by the consumer (Supplementary Table S1b). Therefore, the increased distribution of leaflets with medicines (ideally in Nepali and/or English) should be encouraged.

### Assessment of the authenticity investigation

In the authenticity investigation, the use of the postal delivery method in addition to email delivery led to an increase of nearly 28% (from 6 to 16 responses) in manufacturers’ responses. This approach led to valuable data, although no email response had been received for 6 months or longer before the documents were sent by post. This study’s findings may encourage researchers to send authenticity investigation documents by post in addition to email delivery in future studies. The median response times for authenticity responses in this study were 7.5 days for email and 23.5 days for post, with a maximum response time of 47 days. This finding adds to data provided by Hauk et al.^65^ which suggest that comprehensive data collection during authenticity investigation should allow for at least 2 months for manufacturer response.

### Assessment of price–quality relationships

Although the MPRs of some collected CFIX samples and all LST samples were relatively low (Fig. 8), a low unit price was not a key parameter for a simple prediction of quality issues (Fig. 7a–d). On the other hand, three LST samples that failed the dissolution test critically were sold at the highest unit price among all LST samples (Fig. 7d; B-203, B-207, B-215; all produced in Nepal) and exceeded the indicated—and legally binding—maximum retail price by approximately 20%. However, as these samples were visually similar from others of the same labelled product and batch numbers that were sold at standard unit prices, it could be argued that vendors were not aware of the quality issues and had ‘simply’ increased the prices illegally.

No clear association was found between unit price and sample quality based on descriptive statistics due to the absence of a consistent trend, but further statistical analysis would be required to confirm this. It should also be noted that the reported prices were based on documentation by the samplers; original receipts were not available. The collection of payment receipts was deliberately omitted by the samplers, as requesting them is uncommon in Nepalese pharmacies and could have revealed the mystery shoppers.

### Assessment of the small-scale dissolution test

This study marks the first application in a field survey of a novel small-scale dissolution test developed by Rahman and Yoshida et al. Compared with the final USP dissolution test results of the 213 tested samples, the small-scale dissolution test showed overall false-positive and false-negative rates of approximately 1% (0.9%; 95% CI 0.7–1.3) and 6% (5.8%; 95% CI 4.8–6.5), respectively (AZM: 0.1% false-positive [95% CI 0.0–0.5], 15% false-negative [95% CI 13.3–17.4]; CFIX: 0 samples false-positive or false-negative; ESM: 3% false-positive [95% CI 2.5–4.7], 2% false-negative [95% CI 1.2–2.9]; LST: 0 false-positive, 3% false-negative [95% CI 2.5–4.6]).

These findings suggest that the required capacity for dissolution testing may be considerably lower than the USP test capacity, whereas the risk for false positive results remains low, making it a suitable screening technology for identification of poorly dissolving medicines. The technology is particularly useful in contexts in which dissolution testing is not performed because of capacity issues, which are often observed in LMICs^9,10,12–16,33^.

The small-scale dissolution test was applied to the $\left(\genfrac{}{}{0pt}{}{6}{3}\right)=20$ three-unit combinations derived from the first-stage USP dissolution test results for all of the 213 tested ‘real-world’ samples collected in Nepal. These were then compared with the final USP dissolution test judgement of the samples. For computational simplicity and to avoid dramatically overrepresenting the combinations of those that underwent the second and/or third stage. Therefore, only the 20-three unit combinations $\left(\genfrac{}{}{0pt}{}{6}{3}\right)$ from the first stage were used, rather than those from subsequent stages ($\left(\genfrac{}{}{0pt}{}{12}{3}\right)$ or $\left(\genfrac{}{}{0pt}{}{24}{3}\right)$), because the results of the analysis within a sample were expected to be relatively similar given the nature of manufacturing. However, the occurrence of false positives and false negatives can be attributed to the inconsistent dissolution performance throughout the individual units within certain samples (see ‘Supplementary Material_Chemical Analysis Results’ available as Excel table).

By reducing resources such as the amount of dissolution medium and LC mobile phase and run time that are required for analysis and by reducing maintenance and the working time required of the analyst, this small-scale dissolution test offers a cost-effective solution for the large-scale dissolution testing of distributed products and may thus be particularly beneficial in resource-limited contexts. For example, it could serve as a preliminary screening stage prior to full-scale dissolution testing of the poorly dissolving samples identified. When used in this way, false negatives from the small-scale dissolution test would require additional resources for full-scale dissolution testing but would not pose a risk for consumers in terms of pharmacopoeial dissolution quality.

In addition, the test may be useful in medicine quality surveys in which only small unit quantities of a sample can be obtained or to increase the capacity of samples that undergo dissolution testing^35^. However, the test is not appropriate as a regulatory replacement. Given the insufficient reliability of its results, replacing full-scale dissolution testing as a batch release requirement for manufacturers may also not be favourable. The test may be applied to larger data sets in future research to elucidate its broader applicability. In addition, the acceptance criteria may be modified to avoid false-positive judgements. More stringent criteria for the optimisation of the compliance of such a small-scale dissolution test may be worth investigating.

### Assessment of the portable Raman spectrometers

Raman scattering analysis using two portable spectrometers showed promise as rapid, potentially non-destructive screening technologies for detecting the APIs in most AZM, CFIX and LST samples through characteristic peaks observed near specific Raman shift wavenumbers (Supplementary Figs. S8–S13); moreover, CFIX results aligned with previous findings^66^. However, despite consistent analytical conditions, no corresponding peak was observed in the ESM samples compared with the omeprazole reference standard (Supplementary Figs. S14 and S15). This mismatch is not surprising, given the low sensitivity of Raman scattering analysis for APIs in coated formulations. Due to the limited penetration depth of the laser, the technique is particularly susceptible to interference or fluorescence caused by enteric or film coatings – especially when the API content is low relative to the total mass of excipients.

Currently, few evidence supports the application of Raman scattering analysis to identify substandard medicines. Nevertheless, this would be highly desirable, as consumers and the healthcare system must also be protected from substandard products that can potentially cause health hazards, lead to under-treatment, or—in the case of antimicrobials—contribute to the development of antimicrobial resistance^9,10,13,14,33,57–59^. Comparative spectral analysis could benefit from information on excipients, product composition and authentic samples from the same batches as test-failing products. The findings highlight the limitations of relying solely on API peak identification using Raman scattering analysis, particularly when the API content is correct or varies moderately, e.g. exceeds 80%, in both compliant and substandard samples. Future studies should consider formulation-specific characteristics when Raman spectra are interpreted.

### Limitations

A limitation of this study is that sampling in the Saptari district could not be conducted using randomised sampling. Therefore, the SF medicine occurrence estimate of this study may not reflect the true prevalence in both districts and a national prevalence in Nepal. Additionally, twice as many samples per sampling site were taken from the Saptari district than from the Kathmandu district (4 vs. 2 samples) and the collection of individual samples in the Saptari district was not randomised. Both factors potentially biased the prevalence estimates, for instance, the population of failing LST samples such as those labelled with Trade name 3 may have been overrepresented because of unintentional preferential selection. Nepalese licenced vendors usually dispense blisters without secondary packaging material, which lists the amount of outer packaging collected. The study design may have prevented the detection of falsified medicines given only four APIs were collected exclusively from licenced vendors in just two districts. The authenticity investigation was based on the delivery of sample information, e.g. product names, lots, and pictures, but did not include the delivery of sample material to the manufacturers, limiting the information at the manufacturers’ disposal to assess the samples. Additionally, it could not be confirmed that products from non-responding companies were not falsified. Given these limitations, this investigation cannot definitively conclude the absence of falsified medicines. Notably, the SF medicine occurrence estimate of this study was likely biased given that one unit of sample B-110 (Fig. 3), which was visibly damaged and expected to fail the dissolution test, was separated before the dissolution test was conducted for analytical purposes.

### Policy implications and future research

The results of this study should encourage the Nepalese government to continue its focus on combatting poor-quality medicines and ensuring that all medicines distributed in Nepal meet high-quality standards. Additionally, it should continue strengthening its regulatory capacity, aiming to enhance the WHO maturity levels (and therefore regulatory oversight) with a focus on product quality across all approved medicines in Nepal, including imported medicines. Improving pharmaceutical quality of medicines in production, distribution, and storage is crucial to ensure that all medicines distributed in Nepal meet the quality standards. Furthermore, the distribution of correct leaflets with the medicines should be promoted to increase medicine therapy safety.

Up-to-date registration lists of licenced vendors should be readily available from the responsible regional or national authorities for all districts and regions to ensure the integrity of the pharmaceutical supply chain, enabling regulatory compliance of all stakeholders within the chain and mitigating risks with the rapid identification and removal of non-compliant or illicit vendors. This practice would ideally include functional de-registration procedures for expired and unauthorised vendor licences and strategies to ensure the accuracy and update of such lists.

Future research steps could include multi-district sampling, testing a more diverse range of medicines—including additional APIs and dosage strengths—, conducting validation studies of screening technologies under field conditions, and investigating the causes of poor dissolution performance in certain samples, such as issues related to drug integrity or manufacturing problems. Additionally, the small-scale dissolution test (and dissolution testing in general) could be applied in medicine quality studies within and outside Nepal. In some contexts, this test may encourage the adoption of dissolution testing. Portable Raman scattering analysis could serve as a screening technology to detect products with no APIs, one or more APIs that are different from the stated one, or considerably low API content labelled as containing AZM, CFIX and LST, thereby assisting in the removal of these products. Nevertheless, detecting substandard medicines remains a challenge.

## Conclusion

This semi-randomised, cross-sectional study found that nearly 10% of medicines that contained AZM, CFIX, ESM and LST collected from licenced vendors in the Saptari and Kathmandu districts in Nepal were substandard or probably substandard. Notably, no falsified medicines were detected given the limited methods and sampling scope. Medicines against NCDs were significantly more affected than antibiotics, which may contribute to the burden of disease in Nepal.

The small-scale dissolution test showed only roughly 1% false-positive results compared with the USP dissolution test, indicating its suitability as a screening technology to enhance dissolution testing capacity, which is often lacking in resource-limited settings. Portable Raman scattering spectrometers reliably detected AZM, CFIX and LST in nearly all samples in the narrow sense of API presence or absence, which does not necessarily support their broader value for quality testing. Overall, they were unable to distinguish between compliant and substandard samples. The development of more effective screening technologies for substandard medicines remains urgently needed.

Concerted efforts are required to ensure the integrity of medical products in Nepal and worldwide to protect consumers, healthcare systems and economies from harm.

## Supplementary Information

Below is the link to the electronic supplementary material.

Supplementary Material 1

Supplementary Material 2

## Data Availability

The datasets generated during and/or analysed during the current study are available from the corresponding author on reasonable request.
