# Supplementary material for: Medicine quality assessment in Nepal using semi randomised sampling and evaluation of a small scale dissolution test and portable Raman spectrometers
Source: Sci Rep. 2025 Aug 21;15:30746. doi: 10.1038/s41598-025-16340-7 (PMC12370887; doi:10.1038/s41598-025-16340-7)
Supplement: Supplementary file 1 — Supplementary Material 1 [file 41598_2025_16340_MOESM1_ESM.pdf]

Supplementary Material

Table of Contents

Supplementary Material 1: Methods for Chemical Analysis of AZM, CFIX, ESM and LST Samples 2

Supplementary Material 2: USP 41 Compliance Criteria Methods for Chemical Analysis of AZM, CFIX, ESM and LST Samples 15

Supplementary Material 3: Sampling, Sampling Sites and Sample Data 18

- Tabular sample data 18
- Overview of sampling data 29
- Overview of sampling site data 31

Supplementary Material 4: HPLC Validation Data 32

Supplementary Material 5: Chemical Analysis Data 36

- Tabular overview of test results 36
- Overview of test-failing sample data 37
- Detailed description 38
- Individual AZM, CFIX, ESM, and LST HPLC analysis data (corresponding to supplementary excel file) 42

Supplementary Material 6: Representative HPLC Chromatogram Figures 59

Supplementary Material 7: Price Analysis Data 63

Supplementary Material 8: Raman Spectra – Figures and Descriptions 67

Supplementary Material 1: Methods for Chemical Analysis of AZM, CFIX, ESM and LST Samples

Azithromycin (AZM) analysis methods

AZM was analysed using an HPLC system by Hitachi High-Tech Science Corporation (Tokyo, Japan), which was set up as described in the Devices and Instrumentation section.

AZM – Preparation of the mobile phase:

Following the USP 41 instructions for the preparation of the mobile phase buffer for the AZM tablets assay [47], 2.400 g potassium dihydrogen phosphate and 8.546 g dibasic potassium hydrogen phosphate were weighed into a 2,000-mL volumetric flask, which was partially filled with distilled water, and sonicated after which the total volume was made 2,000 mL. The resulting solution was filled into a 2,000-mL or a 3,000-mL conical flask and stirred using a magnetic stirrer. The mixed solution was adjusted to a pH of 7.5 using NaOH and H_3PO_4. This pH 7.5 buffer solution was filtered using a 0.45-µm membrane filter.

MeCN was degassed for 30 min. The mobile phase was used in isocratic flow in a ratio of 35:65 of the filtered pH 7.5 buffer solution and MeCN (v/v; Buffer:MeCN) and mixed by the HPLC system.

AZM – Preparation of the diluent:

A pH 7.5 buffer solution was prepared in the same manner as described for the mobile phase preparation (see the AZM – Preparation for the mobile phase section of this supplementary material), mixed with MeCN in a ratio of 50:50 (v/v; Buffer:MeCN) and the mixture was stirred for at least 30 min using a magnetic stirrer.

AZM – Preparation of the calibration curve:

The standard solution of AZM was prepared by carefully weighing approximately 11.08 mg AZM dihydrate RS and dissolving it in a 10-mL volumetric flask using the diluent, and sonicating the solution for 6 min. After 3 min of sonication, the mixture was shaken and immediately placed back into the sonicator. After the solution had cooled down to room temperature, the 10-mL volumetric flask was carefully filled up to the mark with diluent using a glass pipette and vortexed. The resulting solution was transferred to a 100-mL glass beaker.

The standard solution of the internal standard, for which benzophenone was selected, was prepared by dissolving 5.0 mg benzophenone RS in a 100-mL volumetric flask, which was partially filled with the diluent, and sonicating the solution for 3 min. After the solution had cooled down to room temperature, the 100-mL volumetric flask was carefully filled up to the mark with the diluent using a glass pipette, a magnetic stirrer was placed in the 100-mL volumetric flask, and the solution was stirred for at least 30 min. A 5-mL quantity was taken using a 5-mL volumetric pipette and transferred into another 100-mL volumetric flask, filled up to the mark using diluent, transferred to a 200-mL glass beaker, and stirred for another 30 min using a magnetic stirrer.

Six AZM calibration curve solutions in the concentrations 1,043 µg/mL, 730 µg/mL, 584 µg/mL, 438 µg/mL, 292 µg/mL, and 146 µg/mL and an additional three QC AZM solutions concentrations 678 µg/mL, 563 µg/mL, and 490 µg/mL were prepared in 2-mL quantities each by using 2.000 mL, 1.400 mL, 1.120 mL, 0.840 mL, 0.560 mL and 0.280 mL, and 1.300 mL, 1.080 mL and 0.940 mL of the AZM standard solution, respectively, by diluting the AZM standard and adding diluent to make a total volume of 2 mL by using a micropipette. These solutions were vortexed to achieve sufficient mixing.

For each of the solutions, a 0.700-mL quantity was pipetted into a 2.5 mL luer-lock syringe with an attached 0.22-µm PTFE filter, and 0.700 mL of the internal standard solution was added to prepare six AZM and three QC solutions in the nominal concentrations 522 µg/mL, 365 µg/mL, 292 µg/mL, 219 µg/mL, 146 µg/mL and 73 µg/mL for the calibration curve solutions, and 339 µg/mL, 282 µg/mL and 245 µg/mL for the QC solutions, respectively. Each of the final solutions was filtered into a suitable HPLC vial, all of which were sealed using 10 mm HPLC vial septa.

<u>AZM – USP 41 Uniformity of Dosage Units Test</u>:
Sample units were accurately weighed and transferred to 100-mL volumetric flasks, which were filled with diluent to approximately one-third or one-half of the total volume, sealed with Parafilm® by Bemis Company, Inc. (Neenah, WI, USA), and sonicated for at least 60 min; flasks were shaken every 10 min during sonication. If congregates or tablet core remains were visually detected after this 60 min, the sample solution was additionally sonicated using a stronger and smaller sonicator until no congregates or tablet core remains were visible. The visual detection of congregates and tablet core remains was performed under different lighting conditions and the sample solution was held in front of a light source.

After sonication, the sample solution was left to cool down to room temperature, and the volumetric flask was filled up to the 200 mL mark using the diluent. Subsequently, magnetic stirrers were put into the volumetric flasks, and the sample solutions were stirred for at least 20 min on high stirring levels.

A sufficient quantity was taken out of each stirred solution and filtered into test tubes through syringes with attached 0.45-µm PTFE filters. Three-mL quantities diluent were placed into fresh test tubes using 3-mL volumetric pipettes, and 0.866 mL of the previously filtered sample solution was added to it and vortexed to make a 3.866 mL solution. A 0.700-mL quantity of each of these processed sample solutions was taken into syringes with attached 0.22-µm PTFE filters using a micropipette, and 0.700 mL internal standard solution was added, the solutions were then vortexed and filtered into suitable HPLC vials, which were sealed using 10 mm HPLC vial septa. The final sample solution had a theoretical concentration of 280 µg/mL if the sample contained 500 mg AZM, as labelled on all samples.

AZM – USP 41 Dissolution Test:

A pH 6.0 dissolution medium was prepared by weighing 20.414 g potassium dihydrogen phosphate, which was transferred to a 3,000-mL volumetric flask, filled with distilled water, and sonicated. After the buffer was dissolved, the volumetric flask was filled up to the mark with distilled water, and the buffer solution was transferred to a 3,000-mL conical flask, stirred using a magnetic stirrer, and the pH of the solution was adjusted to pH 6.0 using NaOH and H_3PO_4.

A 900-mL quantity of the pH 6.0 dissolution medium was filled into the dissolution vessels using 1,000-mL ± 2.0 mL graduated cylinders, and the medium was heated to 37.0 °C ± 0.5 °C.

Sample units were carefully weighed and tested for dissolution at 75 rpm over 30 min after which about 5 mL of the sample solution was withdrawn from the by USP specified locations using 5-mL volumetric pipettes. The sample solutions were filtered using 0.45-µm PTFE filters, and 0.700 mL of the sample solution was mixed with 0.700 mL of the internal standard solution in a syringe with a 0.22-µm PTFE filter, and filtered into a suitable HPLC vial, which was sealed using a 10 mm HPLC vial septum. The final sample solution had a theoretical concentration of 277.8 µg/mL if 500 mg AZM of the sample was dissolved in the medium.

AZM – HPLC conditions:

An InertSustain C18 4.6 mm I.D. × 150 mm column (5-µm particle) packing column by GL Sciences Inc. (Tokyo, Japan) (USP L1) was used. The flow rate was maintained at 0.9 mL/min

at a total run time of 18 min. The oven temperature was set to 45 °C, and the injection volume was 20 µL. Photodiode array (PDA) detection was conducted at a wavelength of 210 nm for both AZM and the internal standard benzophenone. Each solution was injected twice into the HPLC system.

Cefixime (CFIX) analysis methods

CFIX was analysed using an HPLC system from Shimadzu Corporation (Kyoto, Japan), which was set up as described in the Devices and Instrumentation section.

CFIX – Preparation of the mobile phase:
Following the USP 41 instruction for the preparation of the mobile phase for the CFIX tablets assay test [47], 20 mL of 0.5 M tetra-butyl ammonium hydroxide solution was pipetted with a 20-mL volumetric pipette into a 1,000-mL volumetric flask, which was then filled up to the mark with distilled water. The solution was transferred to a 1,000-mL or a larger conical flask and stirred using a magnetic stirrer. However, instead of USP's pH 6.5, the solution was adjusted to a pH 6.86 with diluted H_3PO_4, which was prepared by diluting 4 mL concentrated H_3PO_4 to 25 mL using distilled water. This pH 6.86 solution was filtered using a 0.45-µm membrane filter. The mobile phase was used in isocratic flow in a ratio of 72.5:27.5 of the filtered pH 6.86 buffer solution and MeCN (v/v; Buffer:MeCN) and mixed by the HPLC system (the ratio given by USP is 75:25).

CFIX – Preparation of the diluent:
A pH 7.0 buffer solution was prepared by weighing 9.016 g disodium hydrogen phosphate and 4.965 g potassium dihydrogen phosphate and dissolving both in distilled water in the same 1,000-mL volumetric flask using a sonicator, and subsequently filling it up to the mark. The resulting pH 7.0 buffer solution was transferred to a conical flask of sufficient size and stirred using a magnetic stirrer for at least 30 min.

CFIX – Preparation of the calibration curve:
The standard solution of CFIX was prepared by carefully weighing approximately 6.7 mg CFIX trihydrate RS and dissolving it in a 50-mL volumetric flask using the diluent, which was subsequently sonicated for 15 min. After 10 min of sonication, the mixture was shaken and placed back to the sonicator. After the solution had cooled down to room temperature, the 50-mL volumetric flask was filled up to the mark with diluent using a glass pipette and was mixed for at least 20 min using a magnetic stirrer.

The standard solution of the selected internal standard metronidazole was prepared by dissolving 5.0 mg metronidazole RS in diluent in a 50-mL volumetric flask, sonicating the solution for 15 min. After cooling the solution to room temperature, the 50-mL volumetric flask was carefully filled up to the mark using a glass pipette, a magnetic stirrer was placed in the 50-mL volumetric flask, and the solution was stirred for at least 20 min. A 10-mL quantity was taken using a 10-mL volumetric pipette and transferred into another 50-mL volumetric flask, filled up to the mark using diluent, transferred to a 100-mL glass beaker, and stirred for another 30 min using a magnetic stirrer.

Six CFIX calibration curve solutions in the concentrations 40.3 µg/mL (2.000 mL standard solution was added to 4.000 mL diluent), 30.25 µg/mL (1.000 mL standard solution was added to 3.000 mL diluent), 20.17 µg/mL (1.000 mL standard solution was added to 5.000 mL diluent), 15.75 µg/mL (3.000 mL of the 20.17 µg/mL solution was added to 1.000 mL diluent), 10.085 µg/mL (1.000 mL of the 20.17 µg/mL solution was added to 1.000 mL diluent), and 5.0245 µg/mL (1.000 mL of the 10.085 µg/mL solution was added to 1.000 mL diluent) were prepared in 2-mL (5.0245 µg/mL, 10.085 µg/mL), 4-mL (15.75 µg/mL, 30.25 µg/mL) and 6-mL (20.17 µg/mL, 40.3 µg/mL) quantities for the calibration curve solutions and 24.76 µg/mL, 15.92 µg/mL, and 7.08 µg/mL for the QC solutions, respectively, by adding diluent to the CFIX standard- and diluted solutions using a micropipette. The intermediate- and final solutions were vortexed to achieve sufficient mixing.

For each of the solutions, a 0.700-mL quantity was pipetted into a 2.5 mL luer-lock syringe with an attached 0.22-µm PTFE filter, and 0.700 mL of the internal standard solution was added to create six CFIX and three QC solutions in the nominal concentrations 20.15 µg/mL, 15.125 µg/mL, 10.085 µg/mL, 7.875 µg/mL, 5.0425 µg/mL, and 2.51225 µg/mL (calibration curve solutions), and 12.38 µg/mL, 7.96 µg/mL and 3.54 µg/mL (QC solutions), respectively. Each of the final calibration curve solutions were filtered into a suitable HPLC vial, and all of the HPLC vials were sealed using 10 mm HPLC vial septa.

<u>CFIX – USP 41 Uniformity of Dosage Units Test</u>:

Sample units were accurately weighed and transferred to 100-mL volumetric flasks, which were filled with diluent to approximately one-third or one-half of the total volume, sealed with Parafilm® by Bemis Company, Inc. (Neenah, WI, USA), and sonicated for at least 60 min; flasks were shaken every 10 min during sonication. If congregates or tablet core remains were visually detected after this time, the sample solution was additionally sonicated using a stronger and smaller sonicator until no congregates or tablet core remains were visible. The visual

detection of congregates and tablet core remains was performed under different lighting conditions and the sample solution was held in front of a light source.

After sonication, the sample solution was left to cool down to room temperature, and the volumetric flask was filled up to the 200 mL mark using the diluent. Subsequently, magnetic stirrers were placed into the volumetric flasks, and the sample solutions were stirred for at least 20 min on high stirring levels. A 2-mL quantity of the sample solution was placed into a 100-mL volumetric flask using 2-mL volumetric pipettes and the 100-mL volumetric flask was filled up to the mark with diluent. The resulting solution was stirred for another 20 min.

A sufficient quantity of these stirred sample solutions was filtered each into a test tube through a syringe with an attached 0.45-µm PTFE filter. A 0.700-mL quantity of each of these solutions was then placed into a syringe with an attached 0.22-µm PTFE filter using a micropipette and 0.700 mL internal standard solution was added. The final solutions were vortexed and filtered into suitable HPLC vials, all of which were sealed using 10 mm HPLC vial septa. The final sample solution had a theoretical concentration of 10 µg/mL if the sample contained 200 mg CFIX, as labelled on all samples.

<u>CFIX – USP 41 Dissolution Test</u>:

A pH 7.2 dissolution medium was prepared by weighing 20.4 g potassium dihydrogen phosphate, which was transferred to a 3,000-mL volumetric flask, filled with distilled water and sonicated. After the buffer was dissolved, the volumetric flask was filled up to the mark with distilled water, and the buffer solution was transferred to a 3,000-mL conical flask, stirred using a magnetic stirrer, and solution was adjusted to pH 7.2 using NaOH and H_3PO_4.

A 900-mL quantity of the pH 7.2 dissolution medium was filled into the dissolution vessels using 1,000-mL ± 2.0 mL graduated cylinders, and the medium was heated to 37.0 °C ± 0.5 °C.

Sample units were accurately weighed and tested for dissolution at 100 rpm over 45 min. About 5 mL of the sample solution was withdrawn from the by USP specified locations using 5-mL volumetric pipettes. The sample solutions were filtered using 0.45-µm PTFE filters, and 0.700 mL of the sample solution was mixed with 0.700 mL of the internal standard solution in syringes with attached 0.22-µm PTFE filters. The mixed solutions were filtered into suitable HPLC vials, which were sealed using 10 mm HPLC vial septa. The final sample solution had a nominal concentration of 8.88 µg/mL if 200 mg Cefixime of the sample was dissolved in the medium.

CFIX – HPLC conditions:

A Shim-pack CLC-ODS (M) 4.6 mm I.D. × 150 mm (5-µm particle) RP18 column by Shimadzu Corporation (Kyoto, Japan) was used. The flow rate was maintained at 1.0 mL/min at a total run time of 14 min. The oven temperature was set at 40 °C, and the injection volume was 10 µL. PDA detection was conducted at a wavelength of 254 nm for the internal standard metronidazole and 288 nm for CFIX. Each solution was injected twice into the HPLC system.

Esomeprazole (ESM) analysis methods

Omeprazole standard solutions and ESM sample solutions were analysed using an HPLC system by Jasco, Inc. (Tokyo, Japan), which was set up as described in the Devices and Instrumentation section.

ESM – Preparation of the mobile phase:

Following the USP 41 instruction for the preparation of the mobile phase buffer for the ESM magnesium delayed-release capsules assay test [47], 17.8 g sodium dihydrogen phosphate dihydrate (instead of USP's 15.6g) was placed in a 100-mL volumetric flask using distilled water, and 10.65 g disodium hydrogen phosphate was placed in a 500-mL conical flask. A 150-mL quantity of distilled water was added using a 250-mL ± 2 mL graduated cylinder. Both, the 100-mL volumetric flask and the 500-mL conical flask were sealed using Parafilm® by Bemis Company, Inc. (Neenah, WI, USA) and sonicated until the buffer salts had dissolved.

A volume of 10.5 mL of this sodium dihydrogen phosphate solution and 60 mL of the di-sodium hydrogen phosphate solution were taken using 0.5-mL, 10-mL and 30-mL volumetric pipettes and placed in a 1,000-mL volumetric flask, which was filled up to the mark with distilled water. This solution was transferred to a 1,000-mL or larger conical flask, stirred using a magnetic stirrer and adjusted to pH 7.3 using NaOH or H_3PO_4. This pH 7.3 buffer solution was filtered using a 0.45-µm membrane filter.

At the same time, MeCN was degassed for 30 min. The mobile phase was used in isocratic flow in a ratio of 40:60 of MeCN and the filtered pH 7.3 buffer solution (v/v; Buffer:MeCN) and mixed by the HPLC system.

ESM – Preparation of the diluent:

A mass of 5.24 g tri-sodium phosphate 12-Water was placed in a 500-mL volumetric flask and 1.36 g potassium dihydrogen phosphate was placed in a 200-mL volumetric flask, both buffer salts were dissolved in water and filled up to the mark. The tri-sodium phosphate solution was transferred to a 500-mL volumetric flask or larger and stirred using a magnetic stirrer.

The 200 mL potassium dihydrogen phosphate solution was stirred using a magnetic stirrer. From this solution, 60 mL was taken using 30-mL volumetric pipettes and added to the remaining 90 mL of the di-sodium hydrogen phosphate buffer solution, which was used for ESM mobile phase preparation. From this solution, 110 mL was taken using a 250-mL ± 2 mL graduated cylinder and added to the stirring 500 mL tri-sodium phosphate solution to receive a 610 mL buffer solution. After sufficient stirring, this solution was adjusted to a pH of 7.4 with H_3PO_4 and NaOH before 152.5 mL MeCN was added to the 610 mL buffer solution to achieve a ratio of 80:20 of the filtered pH 7.4 buffer and the MeCN (v/v; Buffer:MeCN). This represents the diluent.

<u>ESM – Preparation of the omeprazole calibration curve</u>:

The standard solution of omeprazole was prepared by carefully weighing about 5.0 mg omeprazole RS and dissolving it in a 50-mL volumetric flask using the diluent. The solution was sonicated for 5 min, the mixture was shaken and placed back to the sonicator for another 5 min of sonication. After the solution had cooled down to room temperature, the 50-mL volumetric flask was carefully filled up to the mark using a glass pipette and mixed for at least 20 min using a magnetic stirrer. A 25-mL quantity was taken using a 25-mL volumetric pipette, transferred to another 50-mL volumetric flask, filled up to the mark using diluent, transferred to a 200-mL glass beaker, and stirred for 30 min using a magnetic stirrer.

The standard solution of the selected internal standard lansoprazole was prepared by dissolving 5.0 mg lansoprazole RS in sufficient diluent in a 50-mL volumetric flask and sonicating the solution for 10 min. After the solution had cooled down to room temperature, the 50-mL volumetric flask was filled up to the mark with diluent using a glass pipette, a magnetic stirrer was placed in the 50-mL volumetric flask, and the solution was stirred for at least 20 min. A 9-mL quantity was taken using a 9-mL volumetric pipette and transferred into another 50-mL volumetric flask, filled up to the mark with diluent, transferred to a 200-mL glass beaker, and stirred for 30 min using a magnetic stirrer.

Six omeprazole calibration curve solutions in the concentrations 48 µg/mL (3.840 mL standard solution was added to 0.160 mL diluent), 40 µg/mL (3.200 mL standard solution was added to 0.800 mL diluent), 32 µg/mL (2.560 mL standard solution was added to 1.440 mL diluent), 24 µg/mL (2.000 mL of the 48 µg/mL solution was added to 2.000 mL diluent), 16 µg/mL (2.000 mL of the 32 µg/mL solution was added to 2.000 mL diluent), and 8 µg/mL (2.000 mL of the 8 µg/mL solution was added to 2.000 mL diluent), and an additional three QC solutions concentrations 38 µg/mL (3.040 mL standard solution was added to 0.960 mL diluent), 30

µg/mL (2.400 mL standard solution was added to 1.600 mL diluent), and 20 µg/mL (1.760 mL standard solution was added to 1.760 mL diluent) were prepared in 4-mL quantities each by using a micropipette. The intermediate- and final solutions were vortexed to achieve sufficient mixing.

For each of the solutions, a 0.700-mL quantity was pipetted into a 2.5 mL luer-lock syringe with an attached 0.22-µm PTFE filter, and 0.700 mL of the internal standard solution was added to prepare six omeprazole and three QC solutions in the nominal concentrations 24 µg/mL, 20 µg/mL, 16 µg/mL, 12 µg/mL, 8 µg/mL and 4 µg/mL for the calibration curve solutions and 19 µg/mL, 15 µg/mL and 11 µg/mL for the QC solutions, respectively. Each of the final calibration curve and QC solutions were filtered into a suitable HPLC vial, all of which were sealed using 10 mm HPLC vial septa.

<u>ESM – USP 41 Uniformity of Dosage Units Test</u>:
Sample units were accurately weighed and transferred to 200-mL volumetric flasks, which were filled with diluent to about one-third or one-half of the total volume, sealed with Parafilm® by Bemis Company, Inc. (Neenah, WI, USA), and sonicated for at least 60 min; flasks were shaken every 10 min during sonication. If congregates or tablet core remains were visually detected after this time, the sample solution was additionally sonicated using a stronger and smaller sonicator until no congregates or tablet core remains were visible. The visual detection of congregates and tablet core remains was performed under different lighting conditions and the sample solution was held in front of a light source.

After sonication, the sample solution was left to cool down to room temperature, and the volumetric flask was filled up to the 200 mL mark using the diluent. Subsequently, magnetic stirrers were put into the volumetric flasks, and the sample solutions were stirred for at least 20 min on high stirring levels.

A sufficient quantity of these stirred sample solutions was filtered into test tubes through syringes with attached 0.45-µm PTFE filters. A 5-mL quantity of diluent was placed into fresh test tubes using 5-mL volumetric pipettes, and 1.000 mL of each of the previously filtered sample solutions were added and vortexed, to make 6.000 mL solutions, each. A 0.700-mL quantity of these solutions were placed each into a syringe with an attached 0.22-µm PTFE filter using a micropipette, and 0.700 mL internal standard solution was added. The solutions were vortexed and filtered into suitable HPLC vials, which were sealed using 10 mm HPLC vial septa. The final sample solution had a nominal concentration of 16.67 µg/mL if the sample contained 40 mg ESM, as labelled on all samples.

ESM - USP 41 Dissolution Test:

A volume of 40 mL of 5 M HCl to 2,000 mL was taken into a 2,000-mL volumetric flask by 40-mL volumetric pipettes and diluted to the mark using distilled water. Sufficient 0.1 M HCl solution was prepared following this instruction.

Dissolution buffer was prepared by weighing 36.6 g di-sodium hydrogen phosphate, which was transferred to a 3,000-mL volumetric flask, filled with distilled water, and sonicated. After the buffer was dissolved, the volumetric flask was filled up to the mark with distilled water, and the buffer solution was transferred to a 3,000-mL conical flask and stirred using a magnetic stirrer.

A 0.25 M NaOH solution was prepared by placing 5 mL of 10 M NaOH in a 200-mL volumetric flask, filling it up to the mark with distilled water, and stirring it using a magnetic stirrer.

For the acid stage, a 300-mL quantity of the 0.1 M HCl dissolution medium was filled into the dissolution vessels each by using a 500-mL ± 2.5 mL graduated cylinder, and the medium was heated to 37.0 °C ± 0.5 °C. Sample units were accurately weighed and tested in the acid stage for dissolution at 100 rpm over 120 min after which exactly 5 mL of the sample solution was withdrawn from the locations specified by USP using 5-mL volumetric pipettes, and a 5-mL quantity of fresh 0.1 M HCl solution was added to the vessels. Immediately, a 700-mL quantity of the buffer dissolution medium was filled into the dissolution vessels using 1,000-mL ± 2.0 mL graduated cylinders to receive a pH 6.8 dissolution medium. The buffer stage was tested for another 30 min with the medium remaining heated at 37.0 °C ± 0.5 °C. Following this, exactly 5 mL of the sample solution was withdrawn from the by USP specified locations using 5-mL volumetric pipettes.

The 5 mL acid stage sample solutions were transferred to a 10 mL volumetric containing 3 mL of 0.25 M NaOH, which was filled up with diluent. A 0.700 mL of the sample solution was mixed with 0.700 mL of the internal standard solution in a syringe with an attached 0.22-µm PTFE filter, and the mixed solution was filtered into a suitable HPLC vial, which was then sealed using a 10 mm HPLC vial septum.

The 5 mL buffer stage sample solutions were transferred into a test tube containing 1 mL of 0.25 M NaOH, vortexed, and a 0.700-mL quantity of each sample solution was mixed with 0.700 mL of the internal standard solution in a syringe with an attached 0.22-µm PTFE filter. The mixed solution was filtered into a suitable HPLC vial, which was sealed using a 10 mm

HPLC vial septum. The final sample solution had a nominal concentration of 15.08 µg/mL if 40 mg ESM of the sample was dissolved in the medium.

ESM – HPLC conditions:
A Phenomenex 4.6 mm I.D. × 150 mm (5-µm particle) NX-C18 column by Phenomenex Inc. (Torrance, CA, USA) was used. The flow rate was maintained at 0.8 mL/min at a total run time of 15 min. The oven temperature was set at 40 °C, and the injection volume was 10 µL. PDA detection was conducted at a wavelength of 302 nm for both the ESM and the internal standard lansoprazole. Each solution was injected twice into the HPLC system.

Losartan (LST) analysis methods

LST was analysed using an HPLC system by Hitachi High-Tech Science Corporation (Tokyo, Japan), which was set up as described in the Devices and Instrumentation section.

LST – Preparation of the mobile phase:
Following the USP 41 instruction for the preparation of the mobile phase buffer for the LST tablets potassium assay [47], 2.720 g potassium dihydrogen phosphate and 3.120 g sodium dihydrogen phosphate dihydrate were placed into the same 2,000-mL volumetric flask, which was partially filled up with distilled water. The solution was sonicated and after the buffer was dissolved, the total volume was made up to 2,000 mL, achieving different buffer solution concentrations than the USP (1.36 mg/mL instead of 1.25 mg/mL, and 1.2 mg/mL instead of 1.5 mg/mL). The solution was then filled into a 2,000-mL or larger conical flask and stirred using a magnetic stirrer. However, instead of USP's indicated pH value of 7.0, the mixed solution was adjusted to a pH of 4.0 using diluted H_3PO_4 and filtered using a 0.45-µm membrane filter.

At the same time, MeCN was degassed for 30 min. The mobile phase was used in isocratic flow in a ratio of 50:50 of the filtered pH 4.0 buffer solution and MeCN (v/v; Buffer:MeCN), and it was mixed by the HPLC system.

LST – Preparation of the diluent:
The same pH 4.0 buffer solution as prepared for mobile phase was mixed with MeCN in a ratio of 70:30 (v/v; Buffer:MeCN), and the mixture was stirred for at least 30 min using a magnetic stirrer.

LST – Preparation of the calibration curve:

The standard solution of LST was prepared by carefully weighing about 6.0 mg LST RS and dissolving it with the diluent in a 50-mL volumetric flask, and sonicating the solution for 10 min. The mixture was shaken after 5 min of sonication and placed back to the sonicator. After the solution had cooled down to room temperature, the 50-mL volumetric flask was filled up to the mark using a glass pipette and mixed for at least 20 min. A 20-mL quantity was taken using a 20-mL volumetric pipette and transferred into another 50-mL volumetric flask, filled up to the mark using diluent, transferred to a 100-mL glass beaker, and stirred for 30 min using a magnetic stirrer.

The standard solution of the selected internal standard diclofenac-sodium was prepared by dissolving 5.0 mg diclofenac-sodium RS in diluent in a 50-mL volumetric flask and sonicating the solution for 10 min. After cooling the solution to room temperature, the 50-mL volumetric flask was filled up to the mark with diluent using a glass pipette, a magnetic stirrer was placed in the 50-mL volumetric flask, and the solution was stirred for at least 20 min. A 20-mL quantity was taken using a 20-mL volumetric pipette and transferred into another 50-mL volumetric flask, filled up to the mark with diluent, transferred to a 100-mL glass beaker, and stirred for 30 min using a magnetic stirrer.

Six LST calibration curve solutions in the concentrations 24 µg/mL, 20 µg/mL, 16 µg/mL, 12 µg/mL, 8 µg/mL, and 4 µg/mL and an additional three QC LST solutions concentrations 22 µg/mL, 14 µg/mL, and 6 µg/mL were prepared in 3-mL quantities each by using 1.500 mL, 1.250 mL, 1.000 mL, 0.750 mL, 0.500 mL and 0.250 mL, and 1.375 mL, 0.875 mL and 0.375 mL of the LST standard solution, respectively, and adding diluent using a micropipette to make the total volumes 3 mL each. These solutions were then vortexed to achieve sufficient mixing.

For each of the calibration curve and QC solutions, a 0.700-mL quantity was pipetted into a 2.5 mL luer-lock syringe with an attached 0.22-µm PTFE filter, and 0.700 mL of the internal standard solution was added to create six LST and three QC solutions in the concentrations 12 µg/mL, 10 µg/mL, 8 µg/mL, 6 µg/mL, 4 µg/mL and 2 µg/mL for the calibration curve solutions, and 11 µg/mL, 7 µg/mL and 3 µg/mL for the QC solutions, respectively. Each of the final calibration curve solutions and QC solutions were filtered into a suitable HPLC vial, all of which were sealed using 10 mm HPLC vial septa.

LST – USP 41 Uniformity of Dosage Units Test:

Sample units were accurately weighed and transferred to 200-mL volumetric flasks, which were filled with diluent to about one-third or one-half of the total volume, sealed with Parafilm® by

Bemis Company, Inc. (Neenah, WI, USA), and sonicated for at least 60 min; flasks were shaken every 10 min during sonication. If congregates or tablet core remains were visually detected after this time, the sample solution was additionally sonicated using a stronger and smaller sonicator until no congregates or tablet core remains were visible. The visual detection of congregates and tablet core remains was performed under different lighting conditions and the sample solution was held in front of a light source.

After sonication, the sample solution was left to cool down to room temperature, and the volumetric flask was filled up to the 200 mL mark using the diluent. Subsequently, magnetic stirrers were placed into the volumetric flasks, and the sample solutions were stirred for at least 20 min on high stirring levels.

A sufficient quantity of each stirred sample solution was filtered into a test tube through a syringe with an attached 0.45-µm PTFE filter. A 6-mL quantity of diluent was placed into a fresh test tube using 6-mL volumetric pipettes, and 0.429 mL of the previously filtered sample solution was added to it. The resulting 6.429 mL solution was then vortexed thoroughly to ensure adequate mixing. A 0.700-mL of each of these solutions were taken into a syringe with an attached 0.22-µm PTFE filter using a micropipette, and 0.700 mL internal standard solution was added. The solution was vortexed and filtered into a suitable HPLC vial, which was sealed using a 10 mm HPLC vial septum. The final sample solution had a nominal concentration of 8.33 µg/mL if the sample contained 50 mg LST, as labelled on all samples.

<u>LST – USP 41 Dissolution Test</u>:

As dissolution medium, a 900-mL quantity of distilled, deaerated water was filled into the dissolution vessels using 1,000-mL ± 2.0 mL graduated cylinders, and the medium was heated to 37.0 °C ± 0.5 °C.

Sample units were accurately weighed and tested for dissolution at 50 rpm over 30 min. About 5 mL of the sample solution was withdrawn as per USP’s instruction using 5-mL volumetric pipettes. The sample solutions were filtered into a test tube each, using 0.45-µm PTFE filters. A 1-mL quantity sample solution was added to a 2.333-mL quantity diluent in another test tube, pipetted using a micropipette. The resulting 3.333 mL diluted sample solutions were vortexed, and a 0.700-mL quantity of each diluted sample solution was mixed with 0.700 mL internal standard solution in syringes with attached 0.22-µm PTFE filters. The solutions were filtered into suitable HPLC vials, which were sealed using 10 mm HPLC vial septa. The final sample solution had a nominal concentration of 8.3 µg/mL if 50 mg LST of the sample was dissolved in the medium.

LST – HPLC conditions:

A Mightysil RP-18 GP 4.6 mm I.D. × 150 mm (5-µm particle) Cica-Reagent column by Kanto Chemical Co., Inc. (Tokyo, Japan) was used. The flow rate was maintained at 0.9 mL/min at a total run time of 18 min. The oven temperature was set at 35 °C, and the injection volume was 10 µL. PDA detection was conducted at a wavelength of 250 nm for both the LST and the internal standard diclofenac-sodium. Each solution was injected twice into the HPLC system.

Supplementary Material 2: USP 41 Compliance Criteria Methods for Chemical Analysis of AZM, CFIX, ESM and LST Samples

Azithromycin (AZM) compliance criteria

AZM – USP 41 Uniformity of Dosage Units Test:

First stage – The Acceptance Value (AV) of 10 units is less than or equal to 15, and no individual content is less than 0.85 × M_{ref} or greater than 1.15 × M_{ref}; with M_{ref} being the reference value calculated as defined by USP 41 (corresponding to USP content uniformity test parameter M).

Second stage – The AV of 30 units is less than or equal to 15, and no individual content is less than 0.75 × M_{ref} or greater than 1.25 × M_{ref}.

AZM – USP 41 Assay Test:

The calculated assay value is between 90% and 110% of the labelled API content.

AZM – USP 41 Dissolution Test:

First stage – Out of six units, no individual dissolution rate (Q) is less than 85% (Q_{Test}+5%, Q_{Test} = 80%); with Q_{Test} being indicated as the compliance Q defined by USP 41 (corresponds to USP's dissolution test parameter Q).

Second stage – The average Q of 12 units is equal to or greater than Q_{Test} = 80%, and no individual Q is less than 65% (Q_{Test}-15%).

Third stage – The average Q of 24 units is equal to or greater than Q_{Test} = 80%, not more than two units have an individual Q less than 65% (Q_{Test}-15%), and no individual Q is less than 55% (Q_{Test}-25%).

Cefixime (CFIX) compliance criteria

CFIX – USP 41 Uniformity of Dosage Units Test:
First stage – The AV of 10 units less than or equal to 15, and no individual content is less than 0.85 × M_{ref} or more than 1.15 × M_{ref}.

Second stage – The AV of 30 units is less than or equal to 15, and no individual content is less than 0.75 × M_{ref} or greater than 1.25 × M_{ref}.

CFIX – USP 41 Assay Test:
The calculated assay value is between 90% and 110% of the labelled API content.

CFIX – USP 41 Dissolution Test:
First stage – Out of six units, no individual Q is less than 85% (Q_{Test}+5%, Q_{Test} = 80%).

Second stage – The average Q of 12 units is equal to or greater than Q_{Test} = 80%, and no individual Q is less than 65% (Q_{Test}-15%).

Third stage – The average Q of 24 units is equal to or greater than Q_{Test} = 80%, not more than two units have an individual Q less than 65% (Q_{Test}-15%), and no individual Q is less than 55% (Q_{Test}-25%).

Esomeprazole (ESM) compliance criteria

ESM – USP 41 Uniformity of Dosage Units Test:
First stage – The AV of 10 units is less than or equal to 15, and no individual content is less than 0.85 × M_{ref} or greater than 1.15 × M_{ref}.

Second stage – The AV of 30 units is less than or equal to 15, and no individual content is less than 0.75 × M_{ref} or greater than 1.25 × M_{ref}.

ESM – USP 41 Assay Test:
The calculated assay value is between 90% and 110% of the labelled API content.

ESM – USP 41 Dissolution Test:
First stage: Acid stage – No individual Q exceeds 10%.

First stage: Buffer stage – Out of six units, no individual Q is less than 80% (Q_{Test}+5%, Q_{Test} = 75%).

Second stage: Acid stage – The average Q of 12 units is less than or equal to 10%, and no individual Q is greater than 25%.

Second stage: Buffer stage – The average Q of 12 units is equal to or greater than Q_{Test} = 75%, and no individual Q is less than 60% (Q_{Test}-15%).

Third stage: Acid stage – The average Q of 24 units is less than or equal to 10%, and no individual Q is greater than 25%.

Third stage: Buffer stage – The average Q of 24 units is equal to or greater than Q_{Test} = 75%, not more than two units have an individual Q less than 60% (Q_{Test}-15%), and no individual Q is less than 50% (Q_{Test}-25%).

Losartan (LST) compliance criteria

LST – USP 41 Uniformity of Dosage Units Test:
First stage – The AV of 10 units is less than or equal to 15, and no individual content is less than 0.85 × M_{ref} or greater than 1.15 × M_{ref}.

Second stage – The AV of 30 units is less than or equal to 15, and no individual content is less than 0.75 × M_{ref} or greater than 1.25 × M_{ref}.

LST – USP 41 Assay Test:
The calculated assay value is between 90% and 110% of the labelled API content.

LST – USP 41 Dissolution Test:
First stage – Out six units, no individual Q is less than 80% (Q_{Test}+5%, Q_{Test} = 75%).

Second stage – The average Q of 12 units is equal to or greater than Q_{Test} = 75%, and no individual Q is less than 60% (Q_{Test}-15%).

Third stage – The average Q of 24 units is equal to or greater than Q_{Test} = 75%, not more than two units have an individual Q less than 60% (Q_{Test}-15%), and no individual Q is less than 50% (Q_{Test}-25%).

Supplementary Material 3: Sampling, Sampling Sites and Sample Data

Tabular sample data

Supplementary Table S1a: Anonymized sample product information

Sample ID	Trade name ID	API	Pharmacopoeia labelled	Strength	Dosage form	Coating formulation	Country of manufacture	Manufacturer ID	Batch ID
A-401	Trade name 7	AZM	IP	500 mg	Tablets	Film-coated	Nepal	Manufacturer 6	Batch 10
A-402	Trade name 22	AZM	IP	500 mg	Tablets	Film-coated	Nepal	Manufacturer 20	Batch 21
A-403	Trade name 19	AZM	USP	500 mg	Tablets	Film-coated	Nepal	Manufacturer 16	Batch 16
A-404	Trade name 7	AZM	IP	500 mg	Tablets	Film-coated	Nepal	Manufacturer 6	Batch 10
A-405	Trade name 12	AZM	IP	500 mg	Tablets	Film-coated	India	Manufacturer 7	Batch 3
A-406	Trade name 50	AZM	USP	500 mg	Tablets	Film-coated	Nepal	Manufacturer 18	Batch 98
A-407	Trade name 36	AZM	USP	500 mg	Tablets	Film-coated	Nepal	Manufacturer 1	Batch 46
A-408	Trade name 54	AZM	IP	500 mg	Tablets	Film-coated	India	Manufacturer 29	Batch 105
A-409	Trade name 36	AZM	USP	500 mg	Tablets	Film-coated	Nepal	Manufacturer 1	Batch 46
A-410	Trade name 14	AZM	USP	500 mg	Tablets	Film-coated	Nepal	Manufacturer 8	Batch 9
A-411	Trade name 12	AZM	IP	500 mg	Tablets	Film-coated	India	Manufacturer 7	Batch 3
A-412	Trade name 19	AZM	USP	500 mg	Tablets	Film-coated	Nepal	Manufacturer 16	Batch 16
A-413	Trade name 22	AZM	IP	500 mg	Tablets	Film-coated	Nepal	Manufacturer 20	Batch 21
A-414	Trade name 14	AZM	USP	500 mg	Tablets	Film-coated	Nepal	Manufacturer 8	Batch 9
A-415	Trade name 7	AZM	IP	500 mg	Tablets	Film-coated	Nepal	Manufacturer 6	Batch 10
B-401	Trade name 14	AZM	USP	500 mg	Tablets	Film-coated	Nepal	Manufacturer 8	Batch 9
B-402	Trade name 7	AZM	IP	500 mg	Tablets	Film-coated	Nepal	Manufacturer 6	Batch 10
B-403	Trade name 22	AZM	IP	500 mg	Tablets	Film-coated	Nepal	Manufacturer 20	Batch 21
B-404	Trade name 19	AZM	USP	500 mg	Tablets	Film-coated	Nepal	Manufacturer 16	Batch 16
B-405	Trade name 12	AZM	IP	500 mg	Tablets	Film-coated	India	Manufacturer 7	Batch 3
B-406	Trade name 14	AZM	USP	500 mg	Tablets	Film-coated	Nepal	Manufacturer 8	Batch 9
B-407	Trade name 7	AZM	IP	500 mg	Tablets	Film-coated	Nepal	Manufacturer 6	Batch 10
B-408	Trade name 22	AZM	IP	500 mg	Tablets	Film-coated	Nepal	Manufacturer 20	Batch 21
B-409	Trade name 19	AZM	USP	500 mg	Tablets	Film-coated	Nepal	Manufacturer 16	Batch 16
B-410	Trade name 12	AZM	IP	500 mg	Tablets	Film-coated	India	Manufacturer 7	Batch 3
B-411	Trade name 14	AZM	USP	500 mg	Tablets	Film-coated	Nepal	Manufacturer 8	Batch 9
B-412	Trade name 7	AZM	IP	500 mg	Tablets	Film-coated	Nepal	Manufacturer 6	Batch 10
B-413	Trade name 12	AZM	IP	500 mg	Tablets	Film-coated	India	Manufacturer 7	Batch 3

B-414	Trade name 12	AZM	IP	500 mg	Tablets	Film-coated	India	Manufacturer 7	Batch 3
B-415	Trade name 12	AZM	IP	500 mg	Tablets	Film-coated	India	Manufacturer 7	Batch 3
C-401	Trade name 10	AZM	USP	500 mg	Tablets	Film-coated	Nepal	Manufacturer 14	Batch 53
C-402	Trade name 33	AZM	USP	500 mg	Tablets	Film-coated	India	Manufacturer 10	Batch 25
C-403	Trade name 53	AZM	IP	500 mg	Tablets	Film-coated	Nepal	Manufacturer 34	Batch 64
C-404	Trade name 38	AZM	USP	500 mg	Tablets	Film-coated	Nepal	Manufacturer 25	Batch 83
C-405	Trade name 14	AZM	USP	500 mg	Tablets	Film-coated	Nepal	Manufacturer 8	Batch 9
C-406	Trade name 10	AZM	USP	500 mg	Tablets	Film-coated	Nepal	Manufacturer 14	Batch 27
C-407	Trade name 14	AZM	USP	500 mg	Tablets	Film-coated	Nepal	Manufacturer 8	Batch 63
C-408	Trade name 9	AZM	USP (substance) / IP (Tablets)	500 mg	Tablets	Film-coated	Nepal	Manufacturer 12	Batch 14
C-409	Trade name 7	AZM	IP	500 mg	Tablets	Film-coated	Nepal	Manufacturer 6	Batch 87
C-410	Trade name 9	AZM	USP (substance) / IP (Tablets)	500 mg	Tablets	Film-coated	Nepal	Manufacturer 12	Batch 54
C-411	Trade name 10	AZM	USP	500 mg	Tablets	Film-coated	Nepal	Manufacturer 14	Batch 38
C-412	Trade name 42	AZM	USP	500 mg	Tablets	Film-coated	Nepal	Manufacturer 4	Batch 48
C-413	Trade name 7	AZM	IP	500 mg	Tablets	Film-coated	Nepal	Manufacturer 6	Batch 81
C-414	Trade name 9	AZM	USP (substance) / IP (Tablets)	500 mg	Tablets	Film-coated	Nepal	Manufacturer 12	Batch 14
C-415	Trade name 10	AZM	USP	500 mg	Tablets	Film-coated	Nepal	Manufacturer 14	Batch 65
D-401	Trade name 10	AZM	USP	500 mg	Tablets	Film-coated	Nepal	Manufacturer 14	Batch 27
D-402	Trade name 47	AZM	USP	500 mg	Tablets	Film-coated	Nepal	Manufacturer 19	Batch 61
D-403	Trade name 42	AZM	USP	500 mg	Tablets	Film-coated	Nepal	Manufacturer 4	Batch 48
D-404	Trade name 49	AZM	IP	500 mg	Tablets	Film-coated	Nepal	Manufacturer 33	Batch 103
D-405	Trade name 7	AZM	IP	500 mg	Tablets	Film-coated	Nepal	Manufacturer 6	Batch 59
D-406	Trade name 38	AZM	USP	500 mg	Tablets	Film-coated	Nepal	Manufacturer 25	Batch 96
D-407	Trade name 10	AZM	USP	500 mg	Tablets	Film-coated	Nepal	Manufacturer 14	Batch 38
D-408	Trade name 52	AZM	USP	500 mg	Tablets	Film-coated	Nepal	Manufacturer 23	Batch 99
D-409	Trade name 9	AZM	USP (substance) / IP (Tablets)	500 mg	Tablets	Film-coated	Nepal	Manufacturer 12	Batch 14
D-410	Trade name 9	AZM	USP (substance) / IP (Tablets)	500 mg	Tablets	Film-coated	Nepal	Manufacturer 12	Batch 39
D-411	Trade name 33	AZM	USP	500 mg	Tablets	Film-coated	India	Manufacturer 10	Batch 25
D-412	Trade name 9	AZM	USP (substance) / IP (Tablets)	500 mg	Tablets	Film-coated	Nepal	Manufacturer 12	Batch 14
D-413	Trade name 10	AZM	USP	500 mg	Tablets	Film-coated	Nepal	Manufacturer 14	Batch 27
D-414	Trade name 33	AZM	USP	500 mg	Tablets	Film-coated	India	Manufacturer 10	Batch 25
D-415	Trade name 9	AZM	USP (substance) / IP (Tablets)	500 mg	Tablets	Film-coated	Nepal	Manufacturer 12	Batch 39
A-301	Trade name 25	CFIX	IP	200 mg	Dispersible Tablets	Uncoated	India	Manufacturer 17	Batch 49

A-302	Trade name 11	CFIX	USP	200 mg	Tablets	Film-coated	Nepal	Manufacturer 13	Batch 47
A-303	Trade name 25	CFIX	IP	200 mg	Dispersible Tablets	Uncoated	India	Manufacturer 17	Batch 49
A-304	Trade name 24	CFIX	USP	200 mg	Tablets	Film-coated	Nepal	Manufacturer 21	Batch 12
A-305	Trade name 17	CFIX	IP	200 mg	Dispersible Tablets	Uncoated	India	Manufacturer 7	Batch 19
A-306	Trade name 6	CFIX	BP	200 mg	Dispersible Tablets	Uncoated	Nepal	Manufacturer 4	Batch 4
A-307	Trade name 28	CFIX	USP	200 mg	Dispersible Tablets	Uncoated	Nepal	Manufacturer 18	Batch 28
A-308	Trade name 31	CFIX	IP	200 mg	Tablets	Film-coated	India	Manufacturer 24	Batch 75
A-309	Trade name 6	CFIX	BP	200 mg	Dispersible Tablets	Uncoated	Nepal	Manufacturer 4	Batch 4
A-310	Trade name 15	CFIX	USP (substance) / IP (Tablets)	200 mg	Tablets	Film-coated	Nepal	Manufacturer 2	Batch 30
A-311	Trade name 24	CFIX	USP	200 mg	Tablets	Film-coated	Nepal	Manufacturer 21	Batch 12
A-312	Trade name 17	CFIX	IP	200 mg	Dispersible Tablets	Uncoated	India	Manufacturer 7	Batch 19
A-313	Trade name 15	CFIX	USP (substance) / IP (Tablets)	200 mg	Tablets	Film-coated	Nepal	Manufacturer 2	Batch 74
A-314	Trade name 28	CFIX	USP	200 mg	Dispersible Tablets	Uncoated	Nepal	Manufacturer 18	Batch 28
A-315	Trade name 6	CFIX	BP	200 mg	Dispersible Tablets	Uncoated	Nepal	Manufacturer 4	Batch 4
B-301	Trade name 24	CFIX	USP	200 mg	Tablets	Film-coated	Nepal	Manufacturer 21	Batch 12
B-302	Trade name 17	CFIX	IP	200 mg	Dispersible Tablets	Uncoated	India	Manufacturer 7	Batch 19
B-303	Trade name 39	CFIX	USP	200 mg	Tablets	Film-coated	Nepal	Manufacturer 16	Batch 31
B-304	Trade name 6	CFIX	BP	200 mg	Dispersible Tablets	Uncoated	Nepal	Manufacturer 4	Batch 4
B-305	Trade name 11	CFIX	USP	200 mg	Tablets	Film-coated	Nepal	Manufacturer 13	Batch 37
B-306	Trade name 31	CFIX	IP	200 mg	Tablets	Film-coated	India	Manufacturer 24	Batch 40
B-307	Trade name 37	CFIX	IP	200 mg	Tablets	Film-coated	India	Manufacturer 26	Batch 51
B-308	Trade name 28	CFIX	USP	200 mg	Dispersible Tablets	Uncoated	Nepal	Manufacturer 18	Batch 28
B-309	Trade name 37	CFIX	IP	200 mg	Tablets	Film-coated	India	Manufacturer 26	Batch 51
B-310	Trade name 24	CFIX	USP	200 mg	Tablets	Film-coated	Nepal	Manufacturer 21	Batch 12
B-311	Trade name 17	CFIX	IP	200 mg	Dispersible Tablets	Uncoated	India	Manufacturer 7	Batch 19
B-312	Trade name 39	CFIX	USP	200 mg	Tablets	Film-coated	Nepal	Manufacturer 16	Batch 31
B-313	Trade name 6	CFIX	BP	200 mg	Dispersible Tablets	Uncoated	Nepal	Manufacturer 4	Batch 4
B-314	Trade name 11	CFIX	USP	200 mg	Tablets	Film-coated	Nepal	Manufacturer 13	Batch 37
B-315	Trade name 31	CFIX	IP	200 mg	Tablets	Film-coated	India	Manufacturer 24	Batch 40
C-301	Trade name 11	CFIX	USP	200 mg	Tablets	Film-coated	Nepal	Manufacturer 13	Batch 90
C-302	Trade name 25	CFIX	IP	200 mg	Dispersible Tablets	Uncoated	India	Manufacturer 17	Batch 102
C-303	Trade name 29	CFIX	USP	200 mg	Tablets	Film-coated	Nepal	Manufacturer 5	Batch 45
C-304	Trade name 30	CFIX	USP	200 mg	Tablets	Film-coated	Nepal	Manufacturer 6	Batch 84
C-305	Trade name 15	CFIX	USP (substance) / IP (Tablets)	200 mg	Tablets	Film-coated	Nepal	Manufacturer 2	Batch 36
C-306	Trade name 6	CFIX	BP	200 mg	Dispersible Tablets	Uncoated	Nepal	Manufacturer 4	Batch 109

C-307	Trade name 15	CFIX	USP (substance) / IP (Tablets)	200 mg	Tablets	Film-coated	Nepal	Manufacturer 2	Batch 36
C-308	Trade name 30	CFIX	USP	200 mg	Tablets	Film-coated	Nepal	Manufacturer 6	Batch 33
C-309	Trade name 15	CFIX	USP (substance) / IP (Tablets)	200 mg	Tablets	Film-coated	Nepal	Manufacturer 2	Batch 30
C-310	Trade name 6	CFIX	BP	200 mg	Dispersible Tablets	Uncoated	Nepal	Manufacturer 4	Batch 4
C-311	Trade name 6	CFIX	BP	200 mg	Dispersible Tablets	Uncoated	Nepal	Manufacturer 4	Batch 41
C-312	Trade name 11	CFIX	USP	200 mg	Tablets	Film-coated	Nepal	Manufacturer 13	Batch 60
C-313	Trade name 11	CFIX	USP	200 mg	Tablets	Film-coated	Nepal	Manufacturer 13	Batch 47
C-314	Trade name 15	CFIX	USP (substance) / IP (Tablets)	200 mg	Tablets	Film-coated	Nepal	Manufacturer 2	Batch 30
C-315	Trade name 27	CFIX	USP	200 mg	Tablets	Film-coated	Nepal	Manufacturer 19	Batch 95
D-301	Trade name 27	CFIX	USP	200 mg	Tablets	Film-coated	Nepal	Manufacturer 19	Batch 101
D-302	Trade name 27	CFIX	USP	200 mg	Tablets	Film-coated	Nepal	Manufacturer 19	Batch 52
D-303	Trade name 34	CFIX	IP	200 mg	Tablets	Film-coated	Nepal	Manufacturer 22	Batch 32
D-304	Trade name 34	CFIX	IP	200 mg	Tablets	Film-coated	Nepal	Manufacturer 22	Batch 32
D-305	Trade name 48	CFIX	USP (substance) / IP (Tablets)	200 mg	Tablets	Uncoated	Nepal	Manufacturer 35	Batch 111
D-306	Trade name 45	CFIX	USP	200 mg	Tablets	Film-coated	Nepal	Manufacturer 32	Batch 85
D-307	Trade name 29	CFIX	USP	200 mg	Tablets	Film-coated	Nepal	Manufacturer 5	Batch 45
D-308	Trade name 6	CFIX	BP	200 mg	Dispersible Tablets	Uncoated	Nepal	Manufacturer 4	Batch 41
D-309	Trade name 29	CFIX	USP	200 mg	Tablets	Film-coated	Nepal	Manufacturer 5	Batch 69
D-310	Trade name 55	CFIX	USP	200 mg	Dispersible Tablets	Uncoated	India	Manufacturer 10	Batch 93
D-311	Trade name 30	CFIX	USP	200 mg	Tablets	Film-coated	Nepal	Manufacturer 6	Batch 33
D-312	Trade name 11	CFIX	USP	200 mg	Tablets	Film-coated	Nepal	Manufacturer 13	Batch 92
D-313	Trade name 34	CFIX	IP	200 mg	Tablets	Film-coated	Nepal	Manufacturer 22	Batch 73
D-314	Trade name 25	CFIX	IP	200 mg	Dispersible Tablets	Uncoated	India	Manufacturer 17	Batch 62
D-315	Trade name 17	CFIX	IP	200 mg	Dispersible Tablets	Uncoated	India	Manufacturer 7	Batch 66
A-101	Trade name 5	ESM	IP	40 mg	Tablets	Enteric-coated	India	Manufacturer 3	Batch 7
A-102	Trade name 13	ESM	N/A	40 mg	Capsules	Enteric-coated pellets	Nepal	Manufacturer 4	Batch 1
A-103	Trade name 26	ESM	IP	40 mg	Tablets	Film-coated	Nepal	Manufacturer 1	Batch 23
A-104	Trade name 20	ESM	IP	40 mg	Tablets	Enteric-coated	Nepal	Manufacturer 6	Batch 18
A-105	Trade name 5	ESM	IP	40 mg	Tablets	Enteric-coated	India	Manufacturer 3	Batch 7
A-106	Trade name 56	ESM	IP	40 mg	Tablets	Enteric-coated	India	Manufacturer 7	Batch 88
A-107	Trade name 13	ESM	N/A	40 mg	Capsules	Enteric-coated pellets	Nepal	Manufacturer 4	Batch 1
A-108	Trade name 26	ESM	IP	40 mg	Tablets	Film-coated	Nepal	Manufacturer 1	Batch 23
A-109	Trade name 20	ESM	IP	40 mg	Tablets	Enteric-coated	Nepal	Manufacturer 6	Batch 18
A-110	Trade name 18	ESM	IP	40 mg	Tablets	Enteric-coated	Nepal	Manufacturer 11	Batch 42
A-111	Trade name 20	ESM	IP	40 mg	Tablets	Enteric-coated	Nepal	Manufacturer 6	Batch 18

A-112	Trade name 13	ESM	N/A	40 mg	Capsules	Enteric-coated pellets	Nepal	Manufacturer 4	Batch 1
A-113	Trade name 20	ESM	IP	40 mg	Tablets	Enteric-coated	Nepal	Manufacturer 6	Batch 18
A-114	Trade name 13	ESM	N/A	40 mg	Capsules	Enteric-coated pellets	Nepal	Manufacturer 4	Batch 1
A-115	Trade name 13	ESM	N/A	40 mg	Capsules	Enteric-coated pellets	Nepal	Manufacturer 4	Batch 1
B-101	Trade name 26	ESM	IP	40 mg	Tablets	Film-coated	Nepal	Manufacturer 1	Batch 23
B-102	Trade name 32	ESM	N/A	40 mg	Tablets	Enteric-coated	Nepal	Manufacturer 1	Batch 44
B-103	Trade name 1	ESM	IP	40 mg	Tablets	Enteric-coated	Nepal	Manufacturer 2	Batch 8
B-104	Trade name 21	ESM	BP	40 mg	Tablets	Enteric-coated	India	Manufacturer 10	Batch 15
B-105	Trade name 26	ESM	IP	40 mg	Tablets	Film-coated	Nepal	Manufacturer 1	Batch 23
B-106	Trade name 32	ESM	N/A	40 mg	Tablets	Enteric-coated	Nepal	Manufacturer 1	Batch 44
B-107	Trade name 1	ESM	IP	40 mg	Tablets	Enteric-coated	Nepal	Manufacturer 2	Batch 8
B-108	Trade name 13	ESM	N/A	40 mg	Capsules	Enteric-coated pellets	Nepal	Manufacturer 4	Batch 1
B-109	Trade name 13	ESM	N/A	40 mg	Capsules	Enteric-coated pellets	Nepal	Manufacturer 4	Batch 1
B-110	Trade name 21	ESM	BP	40 mg	Tablets	Enteric-coated	India	Manufacturer 10	Batch 15
B-111	Trade name 1	ESM	IP	40 mg	Tablets	Enteric-coated	Nepal	Manufacturer 2	Batch 8
B-112	Trade name 21	ESM	BP	40 mg	Tablets	Enteric-coated	India	Manufacturer 10	Batch 15
B-113	Trade name 1	ESM	IP	40 mg	Tablets	Enteric-coated	Nepal	Manufacturer 2	Batch 8
B-114	Trade name 21	ESM	BP	40 mg	Tablets	Enteric-coated	India	Manufacturer 10	Batch 15
B-115	Trade name 1	ESM	IP	40 mg	Tablets	Enteric-coated	Nepal	Manufacturer 2	Batch 29
B-115-2	Trade name 1	ESM	IP	40 mg	Tablets	Enteric-coated	Nepal	Manufacturer 2	Batch 8
C-101	Trade name 51	ESM	IP	40 mg	Tablets	Film-coated	India	Manufacturer 31	Batch 80
C-102	Trade name 5	ESM	IP	40 mg	Tablets	Enteric-coated	India	Manufacturer 3	Batch 82
C-103	Trade name 18	ESM	IP	40 mg	Tablets	Enteric-coated	Nepal	Manufacturer 11	Batch 43
C-104	Trade name 41	ESM	IP	40 mg	Tablets	Enteric-coated	India	Manufacturer 28	Batch 34
C-105	Trade name 1	ESM	IP	40 mg	Tablets	Enteric-coated	Nepal	Manufacturer 2	Batch 57
C-106	Trade name 57	ESM	USP	40 mg	Tablets	Enteric-coated	India	Manufacturer 36	Batch 104
C-107	Trade name 1	ESM	IP	40 mg	Tablets	Enteric-coated	Nepal	Manufacturer 2	Batch 8
C-108	Trade name 58	ESM	IP	40 mg	Tablets	Enteric-coated	Nepal	Manufacturer 22	Batch 100
C-109	Trade name 41	ESM	IP	40 mg	Tablets	Enteric-coated	India	Manufacturer 28	Batch 34
C-110	Trade name 18	ESM	IP	40 mg	Tablets	Enteric-coated	Nepal	Manufacturer 11	Batch 42
C-111	Trade name 59	ESM	IP	40 mg	Tablets	Enteric-coated	Nepal	Manufacturer 5	Batch 68
C-112	Trade name 1	ESM	IP	40 mg	Tablets	Enteric-coated	Nepal	Manufacturer 2	Batch 29
C-113	Trade name 1	ESM	IP	40 mg	Tablets	Enteric-coated	Nepal	Manufacturer 2	Batch 112
C-114	Trade name 1	ESM	IP	40 mg	Tablets	Enteric-coated	Nepal	Manufacturer 2	Batch 29
C-115	Trade name 1	ESM	IP	40 mg	Tablets	Enteric-coated	Nepal	Manufacturer 2	Batch 110
D-101	Trade name 1	ESM	IP	40 mg	Tablets	Enteric-coated	Nepal	Manufacturer 2	Batch 113
D-102	Trade name 1	ESM	IP	40 mg	Tablets	Enteric-coated	Nepal	Manufacturer 2	Batch 55
D-103	Trade name 5	ESM	IP	40 mg	Tablets	Enteric-coated	India	Manufacturer 3	Batch 7
D-104	Trade name 5	ESM	IP	40 mg	Tablets	Enteric-coated	India	Manufacturer 3	Batch 7

D-105	Trade name 46	ESM	IP	40 mg	Tablets	Enteric-coated	Nepal	Manufacturer 30	Batch 72
D-106	Trade name 1	ESM	IP	40 mg	Tablets	Enteric-coated	Nepal	Manufacturer 2	Batch 50
D-107	Trade name 1	ESM	IP	40 mg	Tablets	Enteric-coated	Nepal	Manufacturer 2	Batch 50
D-108	Trade name 5	ESM	IP	40 mg	Tablets	Enteric-coated	India	Manufacturer 3	Batch 7
D-109	Trade name 18	ESM	IP	40 mg	Tablets	Enteric-coated	Nepal	Manufacturer 11	Batch 107
D-110	Trade name 18	ESM	IP	40 mg	Tablets	Enteric-coated	Nepal	Manufacturer 11	Batch 43
D-111	Trade name 5	ESM	IP	40 mg	Tablets	Enteric-coated	India	Manufacturer 3	Batch 7
D-112	Trade name 44	ESM	IP	40 mg	Tablets	Film-coated	Nepal	Manufacturer 8	Batch 71
D-113	Trade name 5	ESM	IP	40 mg	Tablets	Enteric-coated	India	Manufacturer 3	Batch 58
D-114	Trade name 5	ESM	IP	40 mg	Tablets	Enteric-coated	India	Manufacturer 3	Batch 70
D-115	Trade name 32	ESM	N/A	40 mg	Tablets	Enteric-coated	Nepal	Manufacturer 1	Batch 94
A-201	Trade name 3	LST	IP	50 mg	Tablets	Film-coated	Nepal	Manufacturer 5	Batch 22
A-202	Trade name 3	LST	IP	50 mg	Tablets	Film-coated	Nepal	Manufacturer 5	Batch 35
A-203	Trade name 23	LST	USP	50 mg	Tablets	Film-coated	Nepal	Manufacturer 8	Batch 13
A-204	Trade name 8	LST	IP	50 mg	Tablets	Film-coated	India	Manufacturer 9	Batch 6
A-205	Trade name 2	LST	N/A (strip) / BP (package)	50 mg	Tablets	Film-coated	Nepal	Manufacturer 1	Batch 2
A-206	Trade name 8	LST	IP	50 mg	Tablets	Film-coated	India	Manufacturer 9	Batch 6
A-207	Trade name 2	LST	N/A (strip) / BP (package)	50 mg	Tablets	Film-coated	Nepal	Manufacturer 1	Batch 2
A-208	Trade name 8	LST	IP	50 mg	Tablets	Film-coated	India	Manufacturer 9	Batch 6
A-209	Trade name 3	LST	IP	50 mg	Tablets	Film-coated	Nepal	Manufacturer 5	Batch 35
A-210	Trade name 3	LST	IP	50 mg	Tablets	Film-coated	Nepal	Manufacturer 5	Batch 22
A-211	Trade name 8	LST	IP	50 mg	Tablets	Film-coated	India	Manufacturer 9	Batch 6
A-212	Trade name 8	LST	IP	50 mg	Tablets	Film-coated	India	Manufacturer 9	Batch 6
A-213	Trade name 3	LST	IP	50 mg	Tablets	Film-coated	Nepal	Manufacturer 5	Batch 22
A-214	Trade name 2	LST	N/A (strip) / BP (package)	50 mg	Tablets	Film-coated	Nepal	Manufacturer 1	Batch 2
A-215	Trade name 8	LST	IP	50 mg	Tablets	Film-coated	India	Manufacturer 9	Batch 56
B-201	Trade name 4	LST	IP	50 mg	Tablets	Film-coated	India	Manufacturer 3	Batch 5
B-202	Trade name 2	LST	N/A (strip) / BP (package)	50 mg	Tablets	Film-coated	Nepal	Manufacturer 1	Batch 2
B-203	Trade name 3	LST	IP	50 mg	Tablets	Film-coated	Nepal	Manufacturer 5	Batch 11
B-204	Trade name 23	LST	USP	50 mg	Tablets	Film-coated	Nepal	Manufacturer 8	Batch 13
B-205	Trade name 4	LST	IP	50 mg	Tablets	Film-coated	India	Manufacturer 3	Batch 5
B-206	Trade name 2	LST	N/A (strip) / BP (package)	50 mg	Tablets	Film-coated	Nepal	Manufacturer 1	Batch 2
B-207	Trade name 3	LST	IP	50 mg	Tablets	Film-coated	Nepal	Manufacturer 5	Batch 11
B-208	Trade name 23	LST	USP	50 mg	Tablets	Film-coated	Nepal	Manufacturer 8	Batch 13
B-209	Trade name 4	LST	IP	50 mg	Tablets	Film-coated	India	Manufacturer 3	Batch 5
B-210	Trade name 2	LST	N/A (strip) / BP (package)	50 mg	Tablets	Film-coated	Nepal	Manufacturer 1	Batch 2
B-211	Trade name 3	LST	IP	50 mg	Tablets	Film-coated	Nepal	Manufacturer 5	Batch 11
B-212	Trade name 4	LST	IP	50 mg	Tablets	Film-coated	India	Manufacturer 3	Batch 5
B-213	Trade name 2	LST	N/A (strip) / BP (package)	50 mg	Tablets	Film-coated	Nepal	Manufacturer 1	Batch 2

B-214	Trade name 4	LST	IP	50 mg	Tablets	Film-coated	India	Manufacturer 3	Batch 5
B-215	Trade name 3	LST	IP	50 mg	Tablets	Film-coated	Nepal	Manufacturer 5	Batch 22
C-201	Trade name 2	LST	N/A (strip) / BP (package)	50 mg	Tablets	Film-coated	Nepal	Manufacturer 1	Batch 24
C-202	Trade name 2	LST	N/A (strip) / BP (package)	50 mg	Tablets	Film-coated	Nepal	Manufacturer 1	Batch 17
C-203	Trade name 4	LST	IP	50 mg	Tablets	Film-coated	India	Manufacturer 3	Batch 86
C-204	Trade name 35	LST	IP	50 mg	Tablets	Film-coated	Nepal	Manufacturer 11	Batch 97
C-205	Trade name 16	LST	IP	50 mg	Tablets	Film-coated	India	Manufacturer 15	Batch 20
C-206	Trade name 3	LST	IP	50 mg	Tablets	Film-coated	Nepal	Manufacturer 5	Batch 11
C-207	Trade name 43	LST	USP	50 mg	Tablets	Film-coated	Nepal	Manufacturer 27	Batch 106
C-208	Trade name 43	LST	USP	50 mg	Tablets	Film-coated	Nepal	Manufacturer 27	Batch 108
C-209	Trade name 40	LST	USP (substance) / BP (Tablets)	50 mg	Tablets	Film-coated	Nepal	Manufacturer 23	Batch 67
C-210	Trade name 4	LST	IP	50 mg	Tablets	Film-coated	India	Manufacturer 3	Batch 26
C-211	Trade name 16	LST	IP	50 mg	Tablets	Film-coated	India	Manufacturer 15	Batch 20
C-212	Trade name 3	LST	IP	50 mg	Tablets	Film-coated	Nepal	Manufacturer 5	Batch 89
C-213	Trade name 16	LST	IP	50 mg	Tablets	Film-coated	India	Manufacturer 15	Batch 20
C-214	Trade name 2	LST	N/A (strip) / BP (package)	50 mg	Tablets	Film-coated	Nepal	Manufacturer 1	Batch 17
C-215	Trade name 2	LST	N/A (strip) / BP (package)	50 mg	Tablets	Film-coated	Nepal	Manufacturer 1	Batch 17
D-201	Trade name 35	LST	IP	50 mg	Tablets	Film-coated	Nepal	Manufacturer 11	Batch 91
D-202	Trade name 16	LST	IP	50 mg	Tablets	Film-coated	India	Manufacturer 15	Batch 77
D-203	Trade name 16	LST	IP	50 mg	Tablets	Film-coated	India	Manufacturer 15	Batch 79
D-204	Trade name 4	LST	IP	50 mg	Tablets	Film-coated	India	Manufacturer 3	Batch 26
D-205	Trade name 2	LST	N/A (strip) / BP (package)	50 mg	Tablets	Film-coated	Nepal	Manufacturer 1	Batch 17
D-206	Trade name 2	LST	N/A (strip) / BP (package)	50 mg	Tablets	Film-coated	Nepal	Manufacturer 1	Batch 24
D-207	Trade name 23	LST	USP	50 mg	Tablets	Film-coated	Nepal	Manufacturer 8	Batch 13
D-208	Trade name 8	LST	IP	50 mg	Tablets	Film-coated	India	Manufacturer 9	Batch 6
D-209	Trade name 8	LST	IP	50 mg	Tablets	Film-coated	India	Manufacturer 9	Batch 78
D-210	Trade name 4	LST	IP	50 mg	Tablets	Film-coated	India	Manufacturer 3	Batch 5
D-211	Trade name 2	LST	N/A (strip) / BP (package)	50 mg	Tablets	Film-coated	Nepal	Manufacturer 1	Batch 24
D-212	Trade name 40	LST	USP (substance) / BP (Tablets)	50 mg	Tablets	Film-coated	Nepal	Manufacturer 23	Batch 76
D-213	Trade name 3	LST	IP	50 mg	Tablets	Film-coated	Nepal	Manufacturer 5	Batch 11
D-214	Trade name 4	LST	IP	50 mg	Tablets	Film-coated	India	Manufacturer 3	Batch 26
D-215	Trade name 16	LST	IP	50 mg	Tablets	Film-coated	India	Manufacturer 15	Batch 20

Supplementary Table S1b: Additional sample data, sampling site data, and authenticity information

Sample ID	Authenticity status	Leaflet enclosed?	Price per unit [US$]	District	Sampling site ID	Priority No.	Shop category
A-401	No response	No	0.2447	SP	Sampling site 18	N/A	Pharmacy
A-402	Authenticated	No	0.2447	SP	Sampling site 12	N/A	Pharmacy
A-403	No response	No	0.2447	SP	Sampling site 18	N/A	Pharmacy
A-404	No response	No	0.2447	SP	Sampling site 16	N/A	Pharmacy
A-405	Authenticated	No	0.2447	SP	Sampling site 17	N/A	N/A
A-406	No response	No	0.2447	SP	Sampling site 8	N/A	Pharmacy
A-407	Authenticated	No	0.2447	SP	Sampling site 28	N/A	Pharmacy
A-408	No response	No	0.2447	SP	Sampling site 6	N/A	Pharmacy
A-409	Authenticated	No	0.2447	SP	Sampling site 14	N/A	Pharmacy
A-410	No response	No	0.2447	SP	Sampling site 19	N/A	Pharmacy
A-411	Authenticated	No	0.2447	SP	Sampling site 25	N/A	Pharmacy
A-412	No response	No	0.2447	SP	Sampling site 10	N/A	Pharmacy
A-413	Authenticated	No	0.2447	SP	Sampling site 15	N/A	Pharmacy
A-414	No response	No	0.2447	SP	Sampling site 4	N/A	Pharmacy
A-415	No response	No	0.2447	SP	Sampling site 2	N/A	Pharmacy
B-401	No response	No	0.2447	SP	Sampling site 22	N/A	Pharmacy
B-402	No response	No	0.2447	SP	Sampling site 13	N/A	Pharmacy
B-403	Authenticated	No	0.2447	SP	Sampling site 7	N/A	Pharmacy
B-404	No response	No	0.2447	SP	Sampling site 21	N/A	Pharmacy
B-405	Authenticated	No	0.2447	SP	Sampling site 9	N/A	Pharmacy
B-406	No response	No	0.2447	SP	Sampling site 29	N/A	Pharmacy
B-407	No response	No	0.2447	SP	Sampling site 26	N/A	Pharmacy
B-408	Authenticated	No	0.2447	SP	Sampling site 27	N/A	Pharmacy
B-409	No response	No	0.2447	SP	Sampling site 24	N/A	Pharmacy
B-410	Authenticated	No	0.2447	SP	Sampling site 20	N/A	Pharmacy
B-411	No response	No	0.2447	SP	Sampling site 3	N/A	Pharmacy
B-412	No response	No	0.2447	SP	Sampling site 5	N/A	Pharmacy
B-413	Authenticated	No	0.2447	SP	Sampling site 11	N/A	Pharmacy
B-414	Authenticated	No	0.2447	SP	Sampling site 23	N/A	Pharmacy
B-415	Authenticated	No	0.2447	SP	Sampling site 1	N/A	Pharmacy
C-401	Authenticated	No	0.2447	KTM	Sampling site 66	199	Pharmacy
C-402	Authenticated	No	0.2447	KTM	Sampling site 40	17	Pharmacy
C-403	No response	No	0.2447	KTM	Sampling site 44	33	Pharmacy
C-404	No response	No	0.2447	KTM	Sampling site 60	129	Pharmacy
C-405	No response	No	0.2447	KTM	Sampling site 45	35	Wholesaler
C-406	Authenticated	No	0.2447	KTM	Sampling site 88	335	Pharmacy
C-407	No response	No	0.2447	KTM	Sampling site 72	257	Pharmacy
C-408	No response	No	0.2447	KTM	Sampling site 71	253	Pharmacy
C-409	No response	No	0.2447	KTM	Sampling site 50	53	Pharmacy
C-410	No response	No	0.2447	KTM	Sampling site 32	3	Pharmacy
C-411	Authenticated	No	0.2447	KTM	Sampling site 63	147	Pharmacy
C-412	Authenticated	No	0.2447	KTM	Sampling site 85	323	Pharmacy
C-413	No response	No	0.2447	KTM	Sampling site 46	39	Pharmacy
C-414	No response	No	0.2447	KTM	Sampling site 65	183	Pharmacy
C-415	Authenticated	No	0.2447	KTM	Sampling site 48	47	Pharmacy
D-401	Authenticated	No	0.2447	KTM	Sampling site 38	12	Pharmacy
D-402	No response	No	0.2447	KTM	Sampling site 43	30	Pharmacy
D-403	Authenticated	No	0.2447	KTM	Sampling site 55	90	Pharmacy
D-404	No response	Yes	0.2447	KTM	Sampling site 56	110	Pharmacy
D-405	No response	No	0.2447	KTM	Sampling site 54	62	Pharmacy
D-406	No response	No	0.2447	KTM	Sampling site 58	120	Pharmacy
D-407	Authenticated	No	0.2447	KTM	Sampling site 59	126	Pharmacy
D-408	Authenticated	No	0.2447	KTM	Sampling site 62	138	Pharmacy
D-409	No response	No	0.2447	KTM	Sampling site 67	212	Pharmacy
D-410	No response	No	0.2447	KTM	Sampling site 74	272	Pharmacy
D-411	Authenticated	No	0.2447	KTM	Sampling site 77	282	Pharmacy
D-412	No response	No	0.2447	KTM	Sampling site 70	252	Pharmacy
D-413	Authenticated	No	0.2447	KTM	Sampling site 83	312	Pharmacy

D-414	Authenticated	No	0.2447	KTM	Sampling site 82	308	Pharmacy
D-415	No response	No	0.2447	KTM	Sampling site 90	346	Pharmacy
A-301	No response	No	0.1166	SP	Sampling site 30	N/A	N/A
A-302	No response	No	0.1631	SP	Sampling site 12	N/A	Pharmacy
A-303	No response	No	0.1166	SP	Sampling site 31	N/A	Pharmacy
A-304	No response	No	0.1631	SP	Sampling site 16	N/A	Pharmacy
A-305	Authenticated	No	0.1631	SP	Sampling site 17	N/A	N/A
A-306	Authenticated	No	0.1631	SP	Sampling site 8	N/A	Pharmacy
A-307	No response	No	0.1631	SP	Sampling site 28	N/A	Pharmacy
A-308	No response	No	0.1876	SP	Sampling site 6	N/A	Pharmacy
A-309	Authenticated	No	0.1631	SP	Sampling site 14	N/A	Pharmacy
A-310	Authenticated	No	0.1631	SP	Sampling site 19	N/A	Pharmacy
A-311	No response	No	0.1631	SP	Sampling site 25	N/A	Pharmacy
A-312	Authenticated	No	0.1631	SP	Sampling site 10	N/A	Pharmacy
A-313	Authenticated	No	0.1631	SP	Sampling site 15	N/A	Pharmacy
A-314	No response	No	0.1631	SP	Sampling site 4	N/A	Pharmacy
A-315	Authenticated	No	0.1631	SP	Sampling site 2	N/A	Pharmacy
B-301	No response	No	0.1631	SP	Sampling site 22	N/A	Pharmacy
B-302	Authenticated	No	0.1631	SP	Sampling site 13	N/A	Pharmacy
B-303	No response	No	0.1631	SP	Sampling site 7	N/A	Pharmacy
B-304	Authenticated	No	0.1631	SP	Sampling site 21	N/A	Pharmacy
B-305	No response	No	0.1631	SP	Sampling site 9	N/A	Pharmacy
B-306	No response	No	0.1896	SP	Sampling site 29	N/A	Pharmacy
B-307	Authenticated	No	0.1340	SP	Sampling site 26	N/A	Pharmacy
B-308	No response	No	0.1631	SP	Sampling site 27	N/A	Pharmacy
B-309	Authenticated	No	0.1347	SP	Sampling site 24	N/A	Pharmacy
B-310	No response	No	0.1631	SP	Sampling site 20	N/A	Pharmacy
B-311	Authenticated	No	0.1631	SP	Sampling site 3	N/A	Pharmacy
B-312	No response	No	0.1631	SP	Sampling site 5	N/A	Pharmacy
B-313	Authenticated	No	N/A	SP	Sampling site 11	N/A	Pharmacy
B-314	No response	No	0.1631	SP	Sampling site 23	N/A	Pharmacy
B-315	No response	No	0.1631	SP	Sampling site 1	N/A	Pharmacy
C-301	No response	No	0.1631	KTM	Sampling site 53	59	Pharmacy
C-302	No response	No	0.1007	KTM	Sampling site 45	35	Wholesaler
C-303	Authenticated	No	0.1631	KTM	Sampling site 88	335	Pharmacy
C-304	No response	No	0.1631	KTM	Sampling site 72	257	Pharmacy
C-305	Authenticated	No	0.1631	KTM	Sampling site 71	253	Pharmacy
C-306	Authenticated	No	0.1631	KTM	Sampling site 50	53	Pharmacy
C-307	Authenticated	No	0.1631	KTM	Sampling site 76	279	Pharmacy
C-308	No response	No	0.1631	KTM	Sampling site 46	39	Pharmacy
C-309	Authenticated	No	0.1631	KTM	Sampling site 85	323	Pharmacy
C-310	Authenticated	No	0.1631	KTM	Sampling site 34	5	Pharmacy
C-311	Authenticated	No	0.1631	KTM	Sampling site 65	183	Pharmacy
C-312	No response	No	0.1631	KTM	Sampling site 42	19	Pharmacy
C-313	No response	No	0.1631	KTM	Sampling site 48	47	Pharmacy
C-314	Authenticated	No	0.1631	KTM	Sampling site 75	273	Pharmacy
C-315	No response	No	0.1631	KTM	Sampling site 36	9	Pharmacy
D-301	No response	No	0.1631	KTM	Sampling site 38	12	Pharmacy
D-302	No response	No	0.1631	KTM	Sampling site 43	30	Pharmacy
D-303	Authenticated	No	0.1631	KTM	Sampling site 52	56	Pharmacy
D-304	Authenticated	No	0.1405	KTM	Sampling site 49	50	Pharmacy
D-305	No response	No	0.1631	KTM	Sampling site 55	90	Pharmacy
D-306	Authenticated	No	0.1631	KTM	Sampling site 56	110	Pharmacy
D-307	Authenticated	No	0.1631	KTM	Sampling site 62	138	Pharmacy
D-308	Authenticated	No	0.1631	KTM	Sampling site 58	120	Pharmacy
D-309	Authenticated	No	0.1631	KTM	Sampling site 67	212	Pharmacy
D-310	Authenticated	No	0.1468	KTM	Sampling site 77	282	Pharmacy
D-311	No response	No	0.1631	KTM	Sampling site 74	272	Pharmacy
D-312	No response	No	0.1631	KTM	Sampling site 80	298	Pharmacy
D-313	Authenticated	No	0.1631	KTM	Sampling site 81	304	Pharmacy
D-314	No response	Yes	0.1168	KTM	Sampling site 82	308	Pharmacy
D-315	Authenticated	No	0.1631	KTM	Sampling site 89	342	Pharmacy

A-101	No response	No	0.1387	SP	Sampling site 18	N/A	Pharmacy
A-102	Authenticated	No	0.0816	SP	Sampling site 12	N/A	Pharmacy
A-103	No response	No	0.1305	SP	Sampling site 18	N/A	Pharmacy
A-104	No response	No	0.0897	SP	Sampling site 16	N/A	Pharmacy
A-105	No response	No	0.1387	SP	Sampling site 17	N/A	N/A
A-106	Authenticated	No	0.1224	SP	Sampling site 8	N/A	Pharmacy
A-107	Authenticated	No	0.0816	SP	Sampling site 28	N/A	Pharmacy
A-108	No response	No	0.1305	SP	Sampling site 6	N/A	Pharmacy
A-109	No response	No	0.1305	SP	Sampling site 14	N/A	Pharmacy
A-110	Authenticated	No	0.0877	SP	Sampling site 19	N/A	Pharmacy
A-111	No response	No	0.0897	SP	Sampling site 25	N/A	Pharmacy
A-112	Authenticated	No	0.0816	SP	Sampling site 10	N/A	Pharmacy
A-113	No response	No	0.0897	SP	Sampling site 15	N/A	Pharmacy
A-114	Authenticated	No	0.0816	SP	Sampling site 4	N/A	Pharmacy
A-115	Authenticated	No	0.0816	SP	Sampling site 2	N/A	Pharmacy
B-101	No response	No	0.1305	SP	Sampling site 22	N/A	Pharmacy
B-102	No response	No	0.0816	SP	Sampling site 13	N/A	Pharmacy
B-103	Authenticated	No	0.0816	SP	Sampling site 7	N/A	Pharmacy
B-104	Authenticated	No	0.0816	SP	Sampling site 21	N/A	Pharmacy
B-105	Authenticated	No	0.1305	SP	Sampling site 9	N/A	Pharmacy
B-106	No response	No	0.0979	SP	Sampling site 29	N/A	Pharmacy
B-107	Authenticated	No	0.0816	SP	Sampling site 26	N/A	Pharmacy
B-108	Authenticated	No	0.0816	SP	Sampling site 27	N/A	Pharmacy
B-109	Authenticated	No	0.0816	SP	Sampling site 24	N/A	Pharmacy
B-110	Authenticated	No	0.0816	SP	Sampling site 20	N/A	Pharmacy
B-111	Authenticated	No	0.0816	SP	Sampling site 3	N/A	Pharmacy
B-112	Authenticated	No	0.0816	SP	Sampling site 5	N/A	Pharmacy
B-113	Authenticated	No	0.0816	SP	Sampling site 11	N/A	Pharmacy
B-114	Authenticated	No	0.0816	SP	Sampling site 23	N/A	Pharmacy
B-115	Authenticated	No	0.0816	SP	Sampling site 1	N/A	Pharmacy
B-115-2	Authenticated	No	0.0816	SP	Sampling site 1	N/A	Pharmacy
C-101	No response	No	0.1746	KTM	Sampling site 66	199	Pharmacy
C-102	No response	No	0.1387	KTM	Sampling site 44	33	Pharmacy
C-103	Authenticated	No	0.0873	KTM	Sampling site 32	3	Pharmacy
C-104	No response	No	0.0856	KTM	Sampling site 39	15	Pharmacy
C-105	Authenticated	No	0.0816	KTM	Sampling site 63	147	Pharmacy
C-106	Authenticated	Yes	0.0979	KTM	Sampling site 47	45	Pharmacy
C-107	Authenticated	No	0.0816	KTM	Sampling site 42	19	Pharmacy
C-108	Authenticated	No	0.0897	KTM	Sampling site 64	171	Pharmacy
C-109	No response	No	0.0862	KTM	Sampling site 69	251	Pharmacy
C-110	Authenticated	No	0.0877	KTM	Sampling site 78	285	Pharmacy
C-111	Authenticated	No	0.1142	KTM	Sampling site 79	293	Pharmacy
C-112	Authenticated	No	0.0816	KTM	Sampling site 76	279	Pharmacy
C-113	Authenticated	No	0.0816	KTM	Sampling site 73	261	N/A
C-114	Authenticated	No	0.0816	KTM	Sampling site 91	359	Pharmacy
C-115	Authenticated	No	0.0816	KTM	Sampling site 61	133	Pharmacy
D-101	Authenticated	No	0.0816	KTM	Sampling site 33	4	Pharmacy
D-102	Authenticated	No	0.0816	KTM	Sampling site 35	8	Pharmacy
D-103	No response	No	0.0924	KTM	Sampling site 37	10	Pharmacy
D-104	No response	No	0.0923	KTM	Sampling site 41	18	Pharmacy
D-105	Authenticated	No	0.0702	KTM	Sampling site 49	50	Pharmacy
D-106	Authenticated	No	0.0816	KTM	Sampling site 51	54	Pharmacy
D-107	Authenticated	No	0.0816	KTM	Sampling site 57	114	Pharmacy
D-108	No response	No	0.0919	KTM	Sampling site 68	236	Pharmacy
D-109	Authenticated	No	0.0877	KTM	Sampling site 70	252	Pharmacy
D-110	Authenticated	No	0.0877	KTM	Sampling site 81	304	Pharmacy
D-111	No response	No	0.0922	KTM	Sampling site 86	332	Pharmacy
D-112	Authenticated	No	0.1256	KTM	Sampling site 84	318	Pharmacy
D-113	No response	No	0.0922	KTM	Sampling site 87	334	Pharmacy
D-114	No response	No	0.0895	KTM	Sampling site 89	342	Pharmacy
D-115	Authenticated	No	0.0979	KTM	Sampling site 90	346	Pharmacy
A-201	Authenticated	No	0.0612	SP	Sampling site 30	N/A	N/A

A-202	Authenticated	No	0.0612	SP	Sampling site 12	N/A	Pharmacy
A-203	Authenticated	No	0.0620	SP	Sampling site 31	N/A	Pharmacy
A-204	No response	No	0.0612	SP	Sampling site 16	N/A	Pharmacy
A-205	No response	No	0.0620	SP	Sampling site 17	N/A	N/A
A-206	No response	No	0.0612	SP	Sampling site 8	N/A	Pharmacy
A-207	No response	No	0.0620	SP	Sampling site 28	N/A	Pharmacy
A-208	No response	No	0.0612	SP	Sampling site 6	N/A	Pharmacy
A-209	Authenticated	No	0.0612	SP	Sampling site 14	N/A	Pharmacy
A-210	Authenticated	No	0.0620	SP	Sampling site 19	N/A	Pharmacy
A-211	No response	No	0.0620	SP	Sampling site 25	N/A	Pharmacy
A-212	No response	No	0.0612	SP	Sampling site 10	N/A	Pharmacy
A-213	Authenticated	No	0.0620	SP	Sampling site 15	N/A	Pharmacy
A-214	No response	No	0.0620	SP	Sampling site 4	N/A	Pharmacy
A-215	No response	No	0.0612	SP	Sampling site 2	N/A	Pharmacy
B-201	No response	No	N/A	SP	Sampling site 22	N/A	Pharmacy
B-202	No response	No	0.0620	SP	Sampling site 13	N/A	Pharmacy
B-203	Authenticated	No	0.0734	SP	Sampling site 7	N/A	Pharmacy
B-204	Authenticated	No	0.0620	SP	Sampling site 21	N/A	Pharmacy
B-205	No response	No	0.0636	SP	Sampling site 9	N/A	Pharmacy
B-206	No response	No	0.0620	SP	Sampling site 29	N/A	Pharmacy
B-207	Authenticated	No	0.0734	SP	Sampling site 26	N/A	Pharmacy
B-208	Authenticated	No	0.0620	SP	Sampling site 27	N/A	Pharmacy
B-209	No response	No	0.0653	SP	Sampling site 24	N/A	Pharmacy
B-210	No response	No	0.0620	SP	Sampling site 20	N/A	Pharmacy
B-211	Authenticated	No	0.0620	SP	Sampling site 3	N/A	Pharmacy
B-212	No response	No	0.0653	SP	Sampling site 5	N/A	Pharmacy
B-213	No response	No	0.0628	SP	Sampling site 11	N/A	Pharmacy
B-214	No response	No	0.0620	SP	Sampling site 23	N/A	Pharmacy
B-215	Authenticated	No	0.0734	SP	Sampling site 1	N/A	Pharmacy
C-201	Authenticated	No	0.0620	KTM	Sampling site 53	59	Pharmacy
C-202	Authenticated	No	0.0620	KTM	Sampling site 40	17	Pharmacy
C-203	No response	No	0.0620	KTM	Sampling site 60	129	Pharmacy
C-204	Authenticated	No	0.0620	KTM	Sampling site 39	15	Pharmacy
C-205	No response	No	0.0620	KTM	Sampling site 47	45	Pharmacy
C-206	Authenticated	No	0.0620	KTM	Sampling site 34	5	Pharmacy
C-207	No response	No	0.0620	KTM	Sampling site 64	171	Pharmacy
C-208	No response	No	0.0620	KTM	Sampling site 61	133	Pharmacy
C-209	Authenticated	No	0.0620	KTM	Sampling site 75	273	Pharmacy
C-210	No response	No	0.0620	KTM	Sampling site 79	293	Pharmacy
C-211	No response	No	N/A	KTM	Sampling site 36	9	Pharmacy
C-212	Authenticated	No	0.0620	KTM	Sampling site 73	261	N/A
C-213	No response	No	N/A	KTM	Sampling site 78	285	Pharmacy
C-214	No response	No	0.0620	KTM	Sampling site 69	251	Pharmacy
C-215	No response	No	0.0620	KTM	Sampling site 91	359	Pharmacy
D-201	Authenticated	No	0.0620	KTM	Sampling site 33	4	Pharmacy
D-202	No response	No	0.0653	KTM	Sampling site 35	8	Pharmacy
D-203	No response	No	0.0620	KTM	Sampling site 37	10	Pharmacy
D-204	No response	No	0.0620	KTM	Sampling site 41	18	Pharmacy
D-205	No response	No	0.0620	KTM	Sampling site 51	54	Pharmacy
D-206	No response	No	0.0620	KTM	Sampling site 52	56	Pharmacy
D-207	Authenticated	No	0.0620	KTM	Sampling site 54	62	Pharmacy
D-208	No response	No	0.0620	KTM	Sampling site 57	114	Pharmacy
D-209	No response	No	0.0620	KTM	Sampling site 59	126	Pharmacy
D-210	No response	No	0.0620	KTM	Sampling site 68	236	Pharmacy
D-211	No response	No	0.0620	KTM	Sampling site 83	312	Pharmacy
D-212	Authenticated	No	0.0620	KTM	Sampling site 80	298	Pharmacy
D-213	Authenticated	No	0.0620	KTM	Sampling site 84	318	Pharmacy
D-214	No response	No	0.0620	KTM	Sampling site 86	332	Pharmacy
D-215	No response	No	0.0620	KTM	Sampling site 87	334	Pharmacy

N/A = Not available / Not documented

SP = Saptari district

KTM = Kathmandu district

Overview of sampling data

Supplementary Table S2a: Sample quantity per active pharmaceutical ingredients in the Saptari and Kathmandu districts

**API**	**Saptari (Team A, B)**	**Sample share**	**Kathmandu (Team C, D)**	**Sample share**	**Total**	**Sample share**
Azithromycin	30	12.4%	30	12.5%	**60**	24.9%
Cefixime	30	12.4%	30	12.5%	**60**	24.9%
Esomeprazole	31	12.8%	30	12.5%	**61**	25.3%
Losartan	30	12.4%	30	12.5%	**60**	24.9%
**Total**	**121**	**50.2%**	**120**	**49.8%**	**241**	
**4 APIs**						

Supplementary Table S2b: Sample quantity per product

**Product**	**Samples**	**Sample share**
Trade name 1	16	6.6%
Trade name 2	14	5.8%
Trade name 3	12	5.0%
Trade name 4	10	4.1%
Trade name 5–7	9	3.7%
Trade name 8	8	3.3%
Trade name 9–14	7	2.9%
Trade name 15–16	6	2.5%
Trade name 17–18	5	2.1%
Trade name 19–26	4	1.7%
Trade name 27–34	3	1.2%
Trade name 35–43	2	0.8%
Trade name 44–59	1	0.4%
**Total**	**241**	
**59 Products**		

Supplementary Table S2c: Sample quantity per batch

**Batch**	**Samples**	**Sample share**
Batch 1–3	7	2.9%
Batch 4–10	6	2.5%
Batch 11	5	2.1%
Batch 12–23	4	1.7%
Batch 24–30	3	1.2%
Batch 31–51	2	0.8%
Batch 52–113	1	0.4%
**Total**	**241**	
**113 Batches**		

Supplementary Table S2d: Sample quantity per manufacturer and per labelled country of origin

Manufacturer	Samples Origin Nepal	Samples Origin India	Samples	Sample share
Manufacturer 1	23		23	9.5%
Manufacturer 2	22		22	9.1%
Manufacturer 3		19	19	7.9%
Manufacturer 4	18		18	7.5%
Manufacturer 5–6	16		16	6.6%
Manufacturer 7		13	13	5.4%
Manufacturer 8	12		12	5.0%
Manufacturer 9–10		8	8	3.3%
Manufacturer 11–14	7		7	2.9%
Manufacturer 15		6	6	2.5%
Manufacturer 16	6		6	2.5%
Manufacturer 17		4	4	1.7%
Manufacturer 18–22	4		4	1.7%
Manufacturer 23	3		3	1.2%
Manufacturer 24		3	3	1.2%
Manufacturer 25,27	2		2	0.8%
Manufacturer 26,28		2	2	0.8%
Manufacturer 29,31,36		1	1	0.4%
Manufacturer 30,32–35	1		1	0.4%
**Total**	**173**	**68**	**241**	
**Sample share**	71.8%	28.2%		
**36 Manufacturers**				

Supplementary Table S3: Sample quantity per dosage formulation and per active pharmaceutical ingredient

Dosage formulation	AZM	CFIX	ESM	LST	Total
Uncoated Dispersible Tablets	0	22	0	0	**22**
Uncoated Tablets	0	1	0	0	**1**
Film-coated Tablets	60	37	6	60	**163**
Enteric-coated Tablets	0	0	48	0	**48**
Enteric-coated Capsules	0	0	7	0	**7**
**Total**	**60**	**60**	**61**	**60**	**241**
**5 Dosage formulations**					

Supplementary Table S4: Sample and product quantity per labelled pharmacopoeia

Pharmacopoeia<br>labelled on packaging or blister	Samples	Sample share	Products	Sample share
IP	124	51.5%	28	47.5%
Mixed:<br>- IP for the formulation<br>- USP for the substance	14	5.8%	3	5.1%
USP	64	26.6%	22	37.3%
BP	13	5.4%	2	3.4%
Mixed:<br>- BP for the formulation<br>- USP for the substance	2	0.8%	1	1.7%
Mixed:<br>- BP indicated on packaging<br>- Not specified on the blister	14	5.8%	1	1.7%
Not specified	10	4.1%	2	3.4%
**Total**	**241**		**59**	
**3 Pharmacopoeiae labelled**				

Overview of sampling site data

Supplementary Table S5a: Sample quantity collected per sampling site

Sampling site No.	Samples	Sample share
Sampling site 1	5	2.1%
Sampling site 2–29	4	1.7%
30–91	2	0.8%
**Total**	**241**	
91 Sampling sites	91	

Supplementary Table 5b: Sample quantity collected per shop category

Shop category	Samples	Sample share
Retail Pharmacy	231	95.9%
Not specified	8	3.3%
Wholesaler	2	0.8%
**Total**	**241**	

Supplementary Table 5c: Shop quantity per shop category sampled from

Shop category	Shops	Shop share
Retail Pharmacy	87	95.6%
Not specified	3	3.3%
Wholesaler	1	1.1%
**Total**	**91**	

Supplementary Material 4: HPLC Validation Data

Supplementary Table S6a: Validation parameters of AZM quantified using HPLC

Analyte	Calibration curve parameters				LOD [µg/mL]	LOQ [µg/mL]	% Recovery				
		Correlation Coefficient (R^2)	Slope	Intercept			QC-Low (245.2 µg/mL)	QC-Medium (281.7 µg/mL)	QC-High (339.1 µg/mL)	Concentration range	Resolution
AZM	**1**	0.99809	0.01128	-0.13845	24.03	72.82	105.7	102.5	101.8	72.8–520.3 µg/mL	8.620
	**2**	0.99976	0.01089	-0.03105	8.51	25.80	98.5	100.2	99.4	73.0–521.2 µg/mL	8.839
	**3**	0.99970	0.01095	-0.07968	9.56	28.98	98.4	97.7	102.9	73.4–524.1 µg/mL	8.793
	**4**	0.99812	0.01124	-0.03616	23.80	72.13	95.8	99.5	101.6	72.8–520.3 µg/mL	8.816
	**5**	0.99891	0.01076	-0.06122	18.13	54.94	99.0	98.7	99.7	72.8–519.8 µg/mL	8.787
	**6**	0.99867	0.01154	-0.04807	19.98	60.56	98.7	96.4	96.6	72.8–519.8 µg/mL	8.793
	**7**	0.99944	0.01083	-0.08556	13.02	39.45	98.3	98.5	99.2	73.2–522.6 µg/mL	8.637
	**8**	0.99811	0.01092	-0.03457	23.86	72.30	101.5	98.6	97.6	72.7–519.4 µg/mL	8.737
	**Mean**	**0.99885**	**0.01105**	**-0.06435**	**17.61**	**53.37**	**99.5**	**99.0**	**99.9**		**8.753**
	**SD**	0.0007	0.0003	+0.0340	6.05	18.34	2.8	1.7	2.0		0.08
	**%RSD**	0.1%	2.3%	-52.8%	34.4%	34.4%	2.8%	1.7%	2.0%		0.9%
	**Precision**	**Intra-day**	**Inter-day (4 days)**		**Repeatability (6 Inj.)**		**Accuracy at QC conc.**	**281.7 µg/mL**	**339.1 µg/mL**		
	**QC low-medium-high**	0.0338% – 2.1283%	2.7475% – 3.1253%		0.78% (%RSD)		**95% CI**	97.74% – 102.57%	97.11% – 102.09%		

The limit of detection (LOD) was determined based on a signal-to-noise ratio of 3.3:1.

The limit of quantification (LOQ) was determined based on a signal-to-noise ratio of 10:1.

Supplementary Table S6b: Validation parameters of CFIX quantified using HPLC

Analyte	Calibration curve parameters				LOD [µg/mL]	LOQ [µg/mL]	% Recovery				
		Correlation Coefficient (R^2)	Slope	Intercept			QC-Low (3.54 µg/mL)	QC-Medium (7.96 µg/mL)	QC-High (12.38 µg/mL)	Concentration range	Resolution
CFIX	**1**	0.99936	0.22454	+0.0234	0.323	0.979	97.8	99.9	100.1	2.6–20.8 µg/mL	>> 2
	**2**	0.99509	0.22385	+0.04115	0.897	2.717	113.3	93.9	99.6	2.7–21.6 µg/mL	>> 2
	**3**	0.99975	0.23101	-0.00683	0.200	0.607	100.7	99.9	100.7	2.7–21.6 µg/mL	>> 2
	**4**	0.99949	0.21616	+0.01767	0.288	0.874	97.0	95.7	98.6	2.7–21.6 µg/mL	>> 2
	**5**	0.99973	0.23131	-0.00624	0.210	0.638	100.5	99.7	100.6	2.7–21.6 µg/mL	>> 2
	**6**	0.99966	0.24753	+0.00941	0.234	0.710	96.3	100.1	101.1	2.6–20.8 µg/mL	>> 2
	**7**	0.99888	0.24985	-0.01044	0.426	1.291	97.7	100.2	100.8	2.6–20.8 µg/mL	>> 2
	**8**	0.99828	0.23989	+0.00454	0.532	1.611	100.0	100.3	98.1	2.7–21.6 µg/mL	>> 2
	**9**	0.99955	0.23422	-0.00961	0.272	0.823	100.8	100.3	100.7	2.7–21.6 µg/mL	>> 2
	**10**	0.99745	0.22794	+0.001657	0.652	1.976	98.4	100.2	99.7	2.7–21.6 µg/mL	>> 2
	**Mean**	**0.99872**	**0.23263**	**+0.006471**	**0.400**	**1.220**	**100.3**	**99.0**	**100.0**		**>> 2**
	**SD**	0.001402	0.01007	+0.0159162	0.216	0.650	4.4	2.1	0.9		
	**%RSD**	0.14041	4.32852	+245.97315	53.501	53.500	4.4	2.1	0.9		

Precision	Intra-day	Inter-day (5 days)	Repeatability (6 Inj.)	Accuracy at QC conc.	7.96 µg/mL	12.38 µg/mL
**QC low-medium-high**	0.3076% – 1.4839%	4.3832% – 5.3329%	0.47% (%RSD)	**95% CI**	99.24% – 102.57%	99.14% – 102.02%

The limit of detection (LOD) was determined based on a signal-to-noise ratio of 3.3:1.

The limit of quantification (LOQ) was determined based on a signal-to-noise ratio of 10:1.

Supplementary Table S6c: Validation parameters of ESM quantified using HPLC

Analyte	Calibration curve parameters				LOD [µg/mL]	LOQ [µg/mL]	% Recovery				
		Correlation Coefficient (R^2)	Slope	Intercept			QC-Low (11.0 µg/mL)	QC-Medium (15.0 µg/mL)	QC-High (19.0 µg/mL)	Concentration range	Resolution
ESM	**1**	0.99848	0.43994	-0.07360	0.9523	2.8857	102.01	101.48	100.64	4.09–24.55 µg/mL	7.622
	**2**	0.99936	0.45513	-0.11498	0.6167	1.8689	103.24	100.27	98.95	4.08–24.46 µg/mL	7.604
	**3**	0.99999	0.48037	-0.04541	0.0849	0.2571	101.43	100.28	100.66	3.96–23.74 µg/mL	7.568
	**4**	0.99965	0.46040	-0.02568	0.4356	1.3200	100.57	100.52	99.85	3.88–23.26 µg/mL	7.589
	**5**	0.99935	0.45902	-0.00022	0.6059	1.8361	100.93	100.08	101.07	4.00–23.98 µg/mL	7.568
	**6**	0.99998	0.45617	-0.02690	0.1019	0.3088	100.59	99.83	101.42	4.06–24.36 µg/mL	7.632
	**Mean**	**0.99947**	**0.45851**	**-0.04780**	**0.4662**	**1.4128**	**101.46**	**100.41**	**100.43**		**7.597**
	**SD**	0.00051	0.01185	+0.03736	0.3048	0.9237	0.87	0.48	0.76		0.023
	**%RSD**	0.05117	2.58543	-78.16382	65.3860	65.3860	0.86	0.48	0.75		0.299
	**Precision**	**Intra-day**	**Inter-day (3 days)**		**Repeatability (6 Inj.)**		**Accuracy at QC conc.**	**15.0 µg/mL**	**19.0 µg/mL**		
	**QC low-medium-high**	0.2181% – 1.0774%	1.9484% – 2.5351%		0.66% (%RSD)		**95% CI**	97.52% – 100.38%	99.49% – 101.37%		

The limit of detection (LOD) was determined based on a signal-to-noise ratio of 3.3:1.

The limit of quantification (LOQ) was determined based on a signal-to-noise ratio of 10:1.

Supplementary Table S6d: Validation parameters of LST quantified using HPLC

Analyte	Calibration curve parameters				LOD [µg/mL]	LOQ [µg/mL]	% Recovery				
		Correlation Coefficient (R^2)	Slope	Intercept			QC-Low (3.0 µg/mL)	QC-Medium (7.0 µg/mL)	QC-High (11.0 µg/mL)	Concentration range	Resolution
	**1**	0.99984	0.09109	+0.00103	0.168	0.509	98.0	100.0	100.0	2.01–12.08 µg/mL	15.951
	**2**	0.99978	0.09704	-0.01599	0.193	0.586	102.7	101.2	98.4	1.98–11.86 µg/mL	15.577
	**3**	0.99992	0.09615	+0.00026	0.202	0.613	97.3	99.0	99.9	2.00–12.02 µg/mL	15.350
	**4**	0.99978	0.09615	+0.00026	0.202	0.613	99.9	101.0	100.2	2.02–12.14 µg/mL	15.071
	**5**	0.99999	0.09575	+0.00080	0.038	0.115	102.9	100.7	99.9	1.99–11.96 µg/mL	14.396
**LST**	**6**	0.99998	0.09431	+0.00236	0.062	0.187	100.5	100.6	99.2	1.97–11.84 µg/mL	14.311
	**7**	0.99994	0.01496	+0.00247	0.118	0.358	97.9	99.2	99.7	2.00–12.00 µg/mL	15.366
	**8**	0.99977	0.09541	-0.00187	0.118	0.358	98.8	98.4	98.5	2.00–11.98 µg/mL	14.878
	**Mean**	**0.99988**	**0.08511**	**-0.00133**	**0.138**	**0.417**	**99.7**	**100.0**	**99.5**		**15.113**
	**SD**	0.00009	0.02657	+0.00568	0.060	0.182	2.0	1.0	0.7		0.530
	**%RSD**	0.00872	31.21543	-426.01252	43.572	43.572	2.0	1.0	0.7		3.510
	**Precision**	**Intra-day**	**Inter-day (4 days)**		**Repeatability (6 Inj.)**		**Accuracy**	**7.0 µg/mL**	**11.0 µg/mL**		
	**QC low-medium-high**	0.00043% – 1.3214%	1.5835% – 1.8079%		0.59% (%RSD)		**95% CI**	97.74% – 100.61%	100.24% – 101.03%		

The limit of detection (LOD) was determined based on a signal-to-noise ratio of 3.3:1.

The limit of quantification (LOQ) was determined based on a signal-to-noise ratio of 10:1

Supplementary Table S7: Analysis results of AZM, CFIX, ESM and LST standard products

API	Test	Unit 1	Unit 2	Unit 3	Unit 4	Unit 5	Unit 6	Unit 7	Unit 8	Unit 9	Unit 10
AZM	Dissolution	95.5	96.7	99.9	97.7	96.1	96.4				
	Uniformity of Dosage Units	99.5	100.3	101.3	101.1	99.9	101.6	97.4	103.6	99.5	103.8
	Assay										**100.8**
CFIX	Dissolution	102.5	104.2	102.5	99.3	100.6	101.1				
	Uniformity of Dosage Units	96.9	102.5	100.6	100.4	96.2	101.2	101.7	101.9	101.2	99.9
	Assay										**100.3**
ESM	Dissolution	95.9	85.5	90.2	97.6	99.2	99.8				
	Uniformity of Dosage Units	97.2	94.4	98.3	95.4	97.1	97.5	99.9	101.0	99.7	99.4
	Assay										**98.0**
LST	Dissolution	97.7	99.5	97.3	94.6	98.1	98.4				
	Uniformity of Dosage Units	104.6	103.4	104.9	103.6	101.0	101.4	101.8	101.7	102.7	99.6
	Assay										**102.5**

Supplementary Material 5: Chemical Analysis Data

Tabular overview of test results

Supplementary Table S8: Overview of the test results of the uniformity of dosage units test (CU)

API	Samples tested in CU	1st stage fails	Interim fail	2nd stage fails	Pass
AZM	60	2	1	0	59
CFIX	60	5	0	1	59
ESM	61	5	2	1	58
LST	60	1	0	1	59
**Total**	**241**	**13**	**3**	**3**	**235**
Sample share		5.4%	1.2%	1.2%	97.5%

Supplementary Table S9: Overview of the test results of the assay test

API	Samples tested in assay test	Fail	Pass
AZM	60	0	60
CFIX	60	3	57
ESM	61	3	58
LST	60	0	60
**Total**	**241**	**6**	**235**
Sample share		2.5%	97.5%

Supplementary Table S10: Overview of the test results of the dissolution test

API	Samples tested in dissolution test	1st stage fails	2nd stage fails	3rd stage fails	Pass
AZM	60	12	1	1	59
CFIX	38	0	0	0	38
ESM	55	8	4	4	51
LST	60	18	14	13	47
**Total**	**213**	**38**	**19**	**18**	**195**
Sample share		17.8%	8.9%	8.5%	91.5%

Overview of test-failing sample data

Supplementary Table S11a: Test-failing (SF) sample quantity per active pharmaceutical ingredient collected in the Saptari and Kathmandu districts

API	SF samples in Saptari (Team A, B)	SF samples in Kathmandu (Team C, D)	Total	SF sample share
AZM	0	1	1	4.2%
CFIX	2	1	3	12.5%
ESM	5	2	7	29.2%
LST	9	4	13	54.2%
**Total**	**16**	**8**	**24**	
**4 of 4 APIs affected**				

Supplementary Table S11b: Test-failing (SF) sample quantity per product

Product	SF samples	SF sample share
Trade name 3	12	50.0%
Trade name 1,5	3	12.5%
Trade name 7,11,21,24,27,43	1	4.2%
**Total**	**24**	
**9 of 59 products affected**		

Supplementary Table S11c: Test-failing (SF) sample quantity per batch

Batch	SF samples of total samples per batch	SF sample share
Batch 11	5 of 5 (100%)	20.8%
Batch 22	4 of 4 (100%)	16.7%
Batch 8	3 of 6 (50%)	12.5%
Batch 35	2 of 2 (100%)	8.3%
Batch 7	2 of 6 (33%)	8.3%
Batch 59,82,89, 95,106	1 of 1 (100%)	4.2%
Batch 37	1 of 2 (50%)	4.2%
Batch 12,15	1 of 4 (25%)	4.2%
**Total**	**24 of 38 (63.16%)**	
**13 of 113 Batches affected**		

Supplementary Table S11d: Test-failing (SF) sample quantity per manufacturer and per labelled country of origin

Manufacturer	SF samples Origin Nepal	SF samples Origin India	SF samples	SF sample share
Manufacturer 5	12	0	12	50.0%
Manufacturer 2	3	0	3	12.5%
Manufacturer 3	0	3	3	12.5%
Manufacturer 6,13,19,21,27	1	0	1	4.2%
Manufacturer 10	0	1	1	4.2%
**Total**	**20**	**4**	**24**	
**SF sample share**	**83.3%**	**16.7%**		
**Samples of 9 out of 36 Manufacturers affected**				

Detailed description

Azithromycin test results

AZM – Uniformity of Dosage Units Test:

Out of the 60 AZM samples, no sample failed the second (= final) stage of the uniformity of dosage units test.

Two samples (3.3%) did not pass the first stage of the test. Out of these, one sample (1.7%; 95% CI 0.0–8.9; A-414; as AV = 18.13 > 15.0) had insufficient sample material to continue the test (= interim fail).

AZM – Assay Test:

None out of the 60 AZM samples failed the assay test.

AZM – Dissolution Test:

One out of the 60 tested AZM samples (1.7%; 95% CI 0.0–8.9; D-405; as individual Q < Q_{Test}-25%) failed the dissolution test. Representative chromatograms for sample D-405 and the AZM standard product are shown in supplementary Fig. S1.

Twelve samples (20.0%) did not pass the first stage of the test, and one sample (1.7%) did not pass the second stage.

Cefixime test results

CFIX – Uniformity of Dosage Units Test:

Out of the 60 CFIX samples, one sample (1.7%; 95% CI 0.0–8.9; C-315; as AV = 18.60 > 15.0) failed the final stage of the uniformity of dosage units test.

Five samples (8.3%) did not pass the first stage of the test.

CFIX – Assay Test:

Three out of the 60 CFIX samples (5.0%; 95% CI 1.0–13.9) failed the assay test (A-304 with 87.7%, B-314 with 89.7%, and C-315 with 88.6%).

CFIX – Dissolution Test:

None out of the 38 tested CFIX samples failed the dissolution test in the first stage.

Esomeprazole test results

ESM – Uniformity of Dosage Units Test:

Out of the 61 ESM samples, one sample (1.6%; 95% CI 0.0–8.8; B-111; as AV = 16.75 > 15.0) failed the final stage of the uniformity of dosage units test.

Five samples (8.2%) did not pass the first stage of the test, of which two samples (3.3%; B-103; AV = 17.88 > 15.0, and B-107; AV = 23.79 > 15.0) had insufficient sample material to continue the test.

ESM – Assay Test:

Three out of the 60 ESM samples (5.0%; 95% CI 1.0–13.7) failed the assay test (B-103 with 87.1%, B-107 with 83.0%, and B-111 with 88.0%).

ESM – Dissolution Test:

Acid stage – None of the tested ESM samples failed the acid stage of the test.

It is noteworthy, that sample B-104 (unit Q7 of the third stage), sample C-103 (unit Q6 of the first stage first stage), and sample D-110 (unit Q3 of the first stage) showed minor drug release (about 1%).

Buffer stage – Four out of the 55 tested ESM samples (7.2%; 95% CI 2.0–17.6; A-105, B-104, C-102 and D-108; as individual $Q < Q_{Test}$-25%) failed the dissolution test. Chromatograms of the ESM standard product and sample A-105, which is representative for the failing units of samples A-105, C-102 and D-108, are shown in supplementary Fig. S2, and of individual units of sample B-104 in supplementary Fig. S3.

Eight samples (14.5%) did not pass the first stage of the test, and four samples (7.2%) did not pass the second stage.

Losartan test results

LST – Uniformity of Dosage Units Test:
Out of the 60 LST samples, one sample (1.7%; 95% CI 0.0–8.9; C-207; as AV = 17.75 > 15.0) failed the final stage of the uniformity of dosage units test. Chromatograms of the LST standard product, and samples B-215 and A-202, which are representative for samples labelled with Batch 22 and Batch 11, and Batch 35, Batch 89 and AP Trade name 3 are shown in supplementary Fig. S4.

One sample (1.7%) did not pass the first stage of the test.

LST – Assay Test:
None out of the 60 LST samples failed the assay test.

LST – Dissolution Test:
Thirteen out of the 60 tested LST samples (21.7%; 95% CI 12.1–34.2; A-201, A-210, A-213, B-203, B-207, B-211, B-215, C-206 and D-213; as individual $Q < Q_{Test}$-25%, A-209; as more than two individual $Qs < Q_{Test}$-15%, and A-202, C-207 and C-212; as average $Q < Q_{Test}$) failed the dissolution test.

Eighteen samples (30%) did not pass the first stage of the test and 14 samples (23.3%) did not pass the second stage.

LST – Authentic products results:

The corresponding authentic product Trade name 3 of samples A-201, A-202, A-209, A-210, A-213, B-203, B-207, B-211, B-215, C-206, C-212 and D-213 passed the uniformity of dosage units test (AV = 5.40) and the assay test (101.74%) but failed the dissolution test because of more than two individual Qs being below Q_{Test}-15%.

The corresponding authentic product AP Trade name 43 (Batch 108) of samples C-207 (Batch 106) and C-208 (Batch 108) passed the uniformity of dosage units test (AV = 14.72), the assay test (106.48%), and the dissolution test (average Q = 80.88%). Notably, AP Trade name 43 had the same labelled batch number as sample C-208, which also passed the dissolution test.

<u>Individual AZM, CFIX, ESM, and LST HPLC analysis data (corresponding to supplementary excel file)</u>

Supplementary Table S12a: AZM dissolution rate of individual units (Q) tested in the dissolution test (in % of the declared content)

	First stage						Second stage						Third stage											
**Sample ID**	**Q1**	**Q2**	**Q3**	**Q4**	**Q5**	**Q6**	**Q1**	**Q2**	**Q3**	**Q4**	**Q5**	**Q6**	**Q1**	**Q2**	**Q3**	**Q4**	**Q5**	**Q6**	**Q7**	**Q8**	**Q9**	**Q10**	**Q11**	**Q12**
**A-401**	83.1	80.6	76.8	79.2	77.9	80.8	100.8	100.5	91.6	73.6	75.8	78.5												
**A-402**	83.6	85.8	83.7	86.4	80.7	82.6	87.8	88.3	86.5	83.5	85.2	83.2												
**A-403**	95.3	98.2	98.8	97.8	97.8	98.4																		
**A-404**	93.9	88.9	83.4	92.6	82.2	80.8	68.7	69.9	72.4	67.8	92.7	76.1												
**A-405**	94.7	95.7	91.3	95.0	95.7	95.1																		
**A-406**	96.0	95.1	95.1	95.5	95.5	95.2																		
**A-407**	91.5	92.2	91.9	93.2	92.0	95.1																		
**A-408**	99.5	102.7	96.5	100.5	99.2	96.4																		
**A-409**	98.4	96.5	95.1	98.8	98.8	91.9																		
**A-410**	96.2	88.4	96.2	96.2	95.5	87.2																		
**A-411**	87.2	87.7	88.1	89.0	88.2	87.7																		
**A-412**	104.3	103.5	100.9	101.7	101.1	104.2																		
**A-413**	88.5	89.9	88.2	87.9	86.9	87.1																		
**A-414**	83.9	99.5	96.2	100.2	95.5	98.8	101.2	96.7	90.7	92.1	89.5	91.8												
**A-415**	91.2	75.9	78.8	79.1	79.9	82.9	82.8	74.6	87.6	74.1	81.2	84.4												
**B-401**	100.3	100.0	96.7	101.4	90.6	93.0																		
**B-402**	71.6	75.8	82.1	87.4	91.1	87.0	86.2	69.1	80.3	86.9	80.7	85.7												
**B-403**	81.1	83.8	84.9	86.0	85.3	84.0	84.0	87.5	84.6	87.9	83.6	88.2												
**B-404**	96.6	95.9	98.8	97.9	96.5	97.9																		
**B-405**	91.1	90.1	90.7	90.5	90.7	92.5																		
**B-406**	94.3	97.6	97.2	97.1	98.3	93.6																		
**B-407**	83.2	90.4	70.5	82.7	84.7	89.6	86.2	81.3	84.1	87.0	77.3	83.6												
**B-408**	84.1	84.1	80.8	83.3	82.1	86.7	86.8	86.0	85.4	82.9	86.9	84.4												
**B-409**	93.3	97.5	96.0	94.8	95.8	94.9																		
**B-410**	92.9	93.3	92.6	88.3	90.6	90.0																		
**B-411**	98.2	98.9	96.8	99.4	94.0	89.9																		
**B-412**	96.8	74.5	76.1	79.9	81.8	76.9	90.5	83.3	83.0	78.9	88.6	81.2												
**B-413**	89.4	88.5	89.7	87.2	89.6	89.1																		
**B-414**	88.4	87.5	87.4	89.6	89.8	90.0																		
**B-415**	88.8	91.5	91.8	94.2	91.9	92.6																		

**C-401**	93.4	91.2	94.3	95.9	93.9	94.5						
**C-402**	94.5	98.8	95.9	98.6	93.9	97.5						
**C-403**	95.7	91.1	100.6	99.0	98.9	100.8						
**C-404**	93.7	97.4	98.7	93.5	93.3	95.2						
**C-405**	100.8	97.3	98.5	86.7	103.8	99.0						
**C-406**	94.3	90.6	93.0	93.3	95.3	93.9						
**C-407**	99.0	92.4	102.5	100.2	101.6	100.0						
**C-408**	101.4	100.0	100.5	94.4	100.4	100.9						
**C-409**	100.5	99.1	96.8	98.2	97.2	99.3						
**C-410**	101.4	103.2	103.7	99.6	101.6	99.5						
**C-411**	96.3	96.6	99.3	100.8	97.6	99.2						
**C-412**	87.7	87.8	86.5	89.7	94.4	89.7						
**C-413**	83.5	80.2	94.3	82.1	76.4	77.2	78.6	77.7	86.8	75.9	83.5	94.5
**C-414**	94.7	94.7	95.5	98.6	95.4	98.2						
**C-415**	98.6	95.8	95.2	97.2	98.1	98.9						
**D-401**	101.0	99.4	98.5	94.3	101.6	98.6						
**D-402**	99.5	97.6	96.2	100.5	98.7	98.7						
**D-403**	92.9	89.5	86.2	88.6	85.7	88.7						
**D-404**	91.1	90.3	93.8	93.7	93.9	93.5						
**D-405**	82.3	90.2	86.3	68.0	45.6	77.7	82.2	72.5	78.2	73.1	77.9	83.8
**D-406**	97.4	97.9	98.5	99.5	98.6	100.1						
**D-407**	102.4	103.1	101.7	96.6	94.9	100.0						
**D-408**	97.7	97.3	97.5	93.7	98.1	95.8						
**D-409**	97.6	102.4	91.8	98.2	101.4	99.4						
**D-410**	100.0	100.0	98.6	99.4	101.0	99.9						
**D-411**	101.2	99.9	97.7	97.5	99.9	98.1						
**D-412**	99.7	100.9	98.4	101.7	100.1	101.2						
**D-413**	96.9	98.0	97.0	98.2	101.0	96.7						
**D-414**	99.3	97.6	99.5	98.5	98.4	99.4						
**D-415**	97.5	98.2	96.0	94.3	97.7	98.2						

Yellow colour: Q is between Q_{Test}+5% and Q_{Test}. Orange colour: Q is between Q_{Test} and Q_{Test}-15%.
Dark red colour: Q is below Q_{Test}-25%.

Supplementary Table S12b: AZM content of individual units (C) tested in the uniformity of dosage units test (in % of the declared content)

	First stage										Second stage																			
**Sample ID**	**C1**	**C2**	**C3**	**C4**	**C5**	**C6**	**C7**	**C8**	**C9**	**C10**	**C1**	**C2**	**C3**	**C4**	**C5**	**C6**	**C7**	**C8**	**C9**	**C10**	**C11**	**C12**	**C13**	**C14**	**C15**	**C16**	**C17**	**C18**	**C19**	**C20**
**A-401**	101	101	105	100	90	95	103	104	102	104																				
**A-402**	103	100	99	101	97	100	104	100	107	106																				
**A-403**	100	102	101	99	101	100	100	101	99	100																				
**A-404**	100	101	99	98	102	100	100	100	102	99																				
**A-405**	103	99	101	98	99	99	103	105	101	103																				
**A-406**	106	96	101	106	106	95	103	106	105	103																				
**A-407**	92	103	91	106	99	105	106	105	99	109																				
**A-408**	95	102	102	96	102	103	105	99	105	108																				
**A-409**	101	99	102	111	104	103	103	103	104	108																				
**A-410**	102	106	105	107	104	105	106	102	105	105																				
**A-411**	100	101	100	99	100	97	97	100	96	97																				
**A-412**	106	111	105	104	105	97	110	99	97	107																				
**A-413**	108	96	105	107	104	107	104	94	108	106																				
**A-414**	106	100	106	113	111	99	114	111	105	108																				
**A-415**	104	101	104	105	108	105	103	97	101	102																				
**B-401**	110	109	106	97	105	109	106	107	101	106																				
**B-402**	108	105	106	93	103	107	105	105	106	100																				
**B-403**	99	101	96	103	99	96	96	101	102	97																				
**B-404**	98	107	95	102	97	104	93	104	104	109																				
**B-405**	93	100	105	105	102	101	103	108	104	93																				
**B-406**	106	107	105	105	104	108	109	106	106	108																				
**B-407**	103	94	106	106	104	104	105	99	104	105																				
**B-408**	99	105	105	106	103	102	105	106	110	98																				
**B-409**	105	97	99	99	105	107	104	102	112	103																				
**B-410**	96	105	106	102	102	102	104	105	104	105																				
**B-411**	107	106	111	110	109	110	107	109	109	112																				
**B-412**	100	93	103	98	103	103	94	104	106	107																				
**B-413**	104	95	99	104	105	95	106	110	99	110																				
**B-414**	110	106	111	107	107	107	106	110	105	110																				

**B-415**	100	107	107	102	106	98	100	108	107	107
**C-401**	89	96	92	90	85	94	87	97	100	95
**C-402**	90	89	102	102	100	98	92	100	92	98
**C-403**	96	97	102	106	103	101	103	95	104	103
**C-404**	97	98	100	95	97	95	98	96	96	100
**C-405**	100	105	99	100	99	99	98	98	103	101
**C-406**	98	100	98	96	96	94	99	99	98	99
**C-407**	98	98	103	101	100	101	102	97	100	101
**C-408**	99	103	102	102	104	104	105	103	102	103
**C-409**	101	100	97	100	101	98	99	99	101	102
**C-410**	104	97	96	103	107	102	103	99	109	110
**C-411**	98	94	97	103	103	106	107	109	110	107
**C-412**	105	90	101	102	98	97	93	107	105	106
**C-413**	100	100	99	98	100	98	98	98	99	100
**C-414**	101	103	101	103	102	101	102	100	101	104
**C-415**	96	99	105	99	104	99	90	99	101	99
**D-401**	101	98	97	100	101	101	100	97	98	100
**D-402**	103	106	98	98	103	108	107	104	101	104
**D-403**	101	94	95	97	100	100	99	102	95	103
**D-404**	96	97	98	98	97	97	99	97	98	99
**D-405**	99	103	100	100	100	99	100	99	101	98
**D-406**	100	100	100	100	100	98	99	100	100	98
**D-407**	99	99	97	98	100	99	98	99	100	100
**D-408**	99	100	97	99	99	98	100	99	100	97
**D-409**	100	99	99	100	99	99	97	97	98	99
**D-410**	98	98	95	94	94	97	96	95	100	96
**D-411**	97	99	99	97	99	98	103	97	96	96
**D-412**	100	98	102	100	98	102	100	100	101	104
**D-413**	101	102	101	98	100	101	104	98	101	102
**D-414**	99	101	103	103	103	101	100	101	103	102
**D-415**	103	100	100	99	101	102	101	100	95	99

**C-401**	93	96	98	94	100	98	91	98	96	99	92	93	97	97	99	96	93	96	98	97

Yellow colour: C is between 0.85 × M_{ref} (= lower value 1) and 90% or between 110% and 1.15 × M_{ref} (= higher value 1).

Supplementary Table S13a: CFIX dissolution rate of individual units (Q) tested in the dissolution test (in % of the declared content)

	First stage					
**Sample ID**	**Q1**	**Q2**	**Q3**	**Q4**	**Q5**	**Q6**
**A-301**						
**A-302**	96.5	99.0	97.5	99.5	97.5	98.1
**A-303**						
**A-304**	93.6	93.8	93.5	95.3	94.6	94.6
**A-305**						
**A-306**						
**A-307**						
**A-308**	96.8	96.1	94.8	94.3	97.4	96.7
**A-309**						
**A-310**	96.4	96.3	96.9	96.6	97.1	96.9
**A-311**	93.9	93.9	93.4	93.5	93.5	92.5
**A-312**						
**A-313**	96.2	93.1	95.3	96.2	91.9	93.4
**A-314**						
**A-315**						
**B-301**	93.2	92.3	93.0	92.9	93.8	94.3
**B-302**						
**B-303**	97.7	102.8	97.2	100.2	98.5	101.0
**B-304**						
**B-305**	93.0	91.4	94.9	90.5	95.2	93.3
**B-306**	93.4	93.7	93.1	92.2	92.9	92.0
**B-307**	96.1	96.9	95.9	95.9	96.1	94.9
**B-308**						
**B-309**	95.7	99.1	97.7	96.6	97.1	96.4
**B-310**	93.7	90.1	91.3	91.9	94.4	94.4
**B-311**						
**B-312**	100.1	100.6	97.1	100.1	101.5	98.1
**B-313**						
**B-314**	89.0	86.4	89.4	89.3	88.9	91.5
**B-315**	87.7	88.4	90.3	89.5	90.2	92.0
**C-301**	88.9	88.7	88.9	89.0	90.2	90.0
**C-302**						
**C-303**	96.0	92.4	95.9	93.2	89.2	92.3
**C-304**	92.3	94.2	92.6	91.0	94.8	90.1
**C-305**	98.2	98.0	96.1	100.2	98.3	94.2
**C-306**						
**C-307**	96.2	100.2	96.7	99.3	96.3	95.9
**C-308**	91.5	92.6	91.8	94.2	93.6	90.0
**C-309**	93.3	94.7	96.4	94.2	97.7	96.7
**C-310**						
**C-311**						
**C-312**	97.1	92.3	96.2	97.2	96.4	90.7
**C-313**	85.5	93.4	92.7	92.4	93.0	93.4
**C-314**	97.7	94.0	94.6	94.0	94.7	98.7
**C-315**	94.6	96.7	93.2	91.5	93.5	91.7
**D-301**	96.4	91.4	96.7	95.9	102.0	99.7
**D-302**	97.3	99.5	97.0	96.9	98.9	95.4
**D-303**	98.7	105.5	108.9	104.9	103.3	108.1
**D-304**	101.8	102.4	98.8	101.6	107.2	98.7
**D-305**	98.1	97.4	99.3	95.4	99.7	87.0
**D-306**	95.6	99.7	97.4	100.0	96.2	98.0
**D-307**	96.3	96.3	93.3	96.5	92.7	95.4
**D-308**						
**D-309**	97.8	100.2	94.0	96.6	95.8	98.7
**D-310**						
**D-311**	92.6	93.9	90.7	93.5	95.3	90.0
**D-312**	92.1	90.0	95.4	92.8	91.6	93.9
**D-313**	102.1	96.6	102.2	99.5	102.8	100.4
**D-314**						
**D-315**						

Supplementary Table S13b: CFIX content of individual units (C) tested in the uniformity of dosage units test (in % of the declared content)

	First stage										Second stage																			
Sample ID	C1	C2	C3	C4	C5	C6	C7	C8	C9	C10	C1	C2	C3	C4	C5	C6	C7	C8	C9	C10	C11	C12	C13	C14	C15	C16	C17	C18	C19	C20
A-301	93	94	95	96	94	97	97	93	94	97																				
A-302	88	92	95	94	92	88	90	89	95	92																				
A-303	94	93	93	94	90	99	94	96	91	94																				
A-304	89	89	90	89	89	89	86	84	87	85																				
A-305	98	94	87	95	96	96	97	87	98	95																				
A-306	98	97	97	94	97	98	98	99	97	101																				
A-307	97	97	96	92	95	97	95	96	97	93																				
A-308	97	93	94	93	95	96	96	96	95	97																				
A-309	95	94	94	94	95	92	96	98	96	89																				
A-310	93	99	91	93	94	91	94	93	95	94																				
A-311	91	91	92	96	92	91	90	91	92	91																				
A-312	98	97	98	97	99	98	99	99	96	100																				
A-313	92	91	91	89	94	93	95	90	94	91																				
A-314	96	93	93	94	90	88	93	96	89	86																				
A-315	94	94	94	91	96	99	98	96	94	93																				
B-301	90	85	86	90	94	90	93	96	94	95																				
B-302	99	96	99	99	98	96	99	99	84	96																				
B-303	104	106	106	104	104	107	106	105	104	103																				
B-304	99	99	93	98	99	98	93	97	101	95																				
B-305	100	95	96	95	96	98	88	96	91	95																				
B-306	93	95	96	96	93	93	94	96	94	98																				
B-307	100	100	100	99	98	101	102	102	100	100																				
B-308	93	93	89	88	93	92	93	97	92	95																				
B-309	96	100	99	101	99	99	100	98	100	100																				
B-310	94	94	93	92	94	90	97	96	95	92																				
B-311	98	99	100	100	100	98	98	96	99	97																				
B-312	93	95	94	96	96	95	100	91	95	92																				
B-313	102	93	99	95	93	95	98	96	98	97																				
B-314	91	87	86	93	90	90	92	85	93	90																				

**B-315**	92	89	88	94	92	91	93	87	94	91																				
**C-301**	90	93	95	92	92	95	92	93	92	91																				
**C-302**	94	95	100	92	95	94	95	95	99	94																				
**C-303**	93	95	91	95	89	94	91	91	90	94																				
**C-304**	91	90	86	95	91	92	90	89	91	89																				
**C-305**	90	98	95	85	92	85	94	94	89	93	91	92	93	96	87	93	98	95	93	94	96	98	80	98	96	99	97	95	94	97
**C-306**	100	102	100	97	98	104	101	104	96	100																				
**C-307**	93	94	88	97	96	97	98	92	99	98																				
**C-308**	92	91	88	89	88	100	94	94	99	95																				
**C-309**	93	94	93	94	95	92	96	96	89	93																				
**C-310**	98	100	95	94	94	95	97	85	95	90																				
**C-311**	96	92	94	98	94	94	95	94	97	93																				
**C-312**	92	92	94	94	92	90	94	90	92	91																				
**C-313**	98	92	91	93	95	95	98	93	98	95																				
**C-314**	94	94	91	96	97	96	98	96	97	97																				
**C-315**	79	93	91	86	79	90	87	92	86	92	89	86	94	89	93	86	96	90	96	84	83	88	89	88	87	84	86	85	93	93
**D-301**	91	91	88	91	88	90	89	91	92	89																				
**D-302**	92	93	81	94	96	95	94	91	93	83	94	92	93	95	96	95	91	97	94	94	94	92	97	96	96	89	96	95	95	93
**D-303**	102	100	101	101	103	99	105	96	95	102																				
**D-304**	92	101	78	95	100	97	100	109	100	104	101	102	100	98	98	99	97	100	96	98	104	102	96	98	101	95	100	99	100	98
**D-305**	98	95	94	97	98	97	99	95	93	93																				
**D-306**	98	90	88	98	94	100	85	90	99	97	94	97	96	96	101	95	95	98	94	96	97	97	97	90	97	97	97	95	95	97
**D-307**	95	94	91	92	95	92	95	94	91	97																				
**D-308**	98	98	90	98	97	96	98	98	100	95																				
**D-309**	99	88	96	94	98	88	94	97	92	92																				
**D-310**	95	96	91	96	94	93	94	93	92	89																				
**D-311**	95	93	87	95	96	90	93	96	92	92																				
**D-312**	91	90	92	91	90	92	92	92	87	89																				
**D-313**	96	98	100	102	100	98	102	101	100	94																				
**D-314**	100	96	90	98	96	98	99	95	100	96																				
**D-315**	95	95	84	96	98	96	94	97	99	92																				

Yellow colour: C is between 0.85 × M_{ref} (= lower value 1) and 90% or between 110% and 1.15 × M_{ref} (= higher value 1).
Red colour: C is between 0.75 × M_{ref} (= lower value 2) and 0.85 × M_{ref} (= lower value 1).

Supplementary Table S14a: ESM dissolution rate of individual units (Q) tested in the dissolution test acid and buffer stages (in % of the declared content)

Acid stage:	First stage						Second stage						Third stage											
**Sample ID**	**Q1**	**Q2**	**Q3**	**Q4**	**Q5**	**Q6**	**Q1**	**Q2**	**Q3**	**Q4**	**Q5**	**Q6**	**Q1**	**Q2**	**Q3**	**Q4**	**Q5**	**Q6**	**Q7**	**Q8**	**Q9**	**Q10**	**Q11**	**Q12**
**A-101**	0	0	0	0	0	0																		
**A-102**	0	0	0	0	0	0																		
**A-103**																								
**A-104**	0	0	0	0	0	0																		
**A-105**	0	0	0	0	0	0	0	0	0	0	0	0	0	0	0	0	0	0	0	0	0	0	0	0
**A-106**	0	0	0	0	0	0																		
**A-107**	0	0	0	0	0	0																		
**A-108**																								
**A-109**	0	0	0	0	0	0																		
**A-110**	0	0	0	0	0	0																		
**A-111**	0	0	0	0	0	0																		
**A-112**	0	0	0	0	0	0																		
**A-113**	0	0	0	0	0	0																		
**A-114**	0	0	0	0	0	0																		
**A-115**	0	0	0	0	0	0																		
**B-101**																								
**B-102**	0	0	0	0	0	0																		
**B-103**	0	0	0	0	0	0	0	0	0	0	0	0												
**B-104**	0	0	0	0	0	0	0	0	0	0	0	0	0	0	0	0	0	0	1	0	0	0	0	0
**B-105**																								
**B-106**	0	0	0	0	0	0																		
**B-107**	0	0	0	0	0	0																		
**B-108**	0	0	0	0	0	0																		
**B-109**	0	0	0	0	0	0																		
**B-110**	0	0	0	0	0	0																		
**B-111**	0	0	0	0	0	0																		
**B-112**	0	0	0	0	0	0																		
**B-113**	0	0	0	0	0	0																		

**B-114**	0	0	0	0	0	0	0	0	0	0	0	0												
**B-115**	0	0	0	0	0	0																		
**B115-2**	0	0	0	0	0	0																		
**C-101**																								
**C-102**	0	0	0	0	0	0	0	0	0	0	0	0	0	0	0	0	0	0	0	0	0	0	0	0
**C-103**	0	0	0	0	0	1	0	0	0	0	0	0												
**C-104**	0	0	0	0	0	0																		
**C-105**	0	0	0	0	0	0																		
**C-106**	0	0	0	0	0	0																		
**C-107**	0	0	0	0	0	0																		
**C-108**	0	0	0	0	0	0																		
**C-109**	0	0	0	0	0	0																		
**C-110**	0	0	0	0	0	0																		
**C-111**	0	0	0	0	0	0	0	0	0	0	0	0												
**C-112**	0	0	0	0	0	0																		
**C-113**	0	0	0	0	0	0																		
**C-114**	0	0	0	0	0	0																		
**C-115**	0	0	0	0	0	0																		
**D-101**	0	0	0	0	0	0																		
**D-102**	0	0	0	0	0	0																		
**D-103**	0	0	0	0	0	0																		
**D-104**	0	0	0	0	0	0																		
**D-105**	0	0	0	0	0	0																		
**D-106**	0	0	0	0	0	0																		
**D-107**	0	0	0	0	0	0																		
**D-108**	0	0	0	0	0	0	0	0	0	0	0	0	0	0	0	0	0	0	0	0	0	0	0	0
**D-109**	0	0	0	0	0	0																		
**D-110**	0	0	1	0	0	0																		
**D-111**	0	0	0	0	0	0																		
**D-112**																								
**D-113**	0	0	0	0	0	0																		
**D-114**	0	0	0	0	0	0																		
**D-115**	0	0	0	0	0	0																		

Yellow colour: Measured ESM release > 0.0% of the label claim.

Buffer stage:	First stage						Second stage						Third stage											
**Sample ID**	**Q1**	**Q2**	**Q3**	**Q4**	**Q5**	**Q6**	**Q1**	**Q2**	**Q3**	**Q4**	**Q5**	**Q6**	**Q1**	**Q2**	**Q3**	**Q4**	**Q5**	**Q6**	**Q7**	**Q8**	**Q9**	**Q10**	**Q11**	**Q12**
**A-101**	101.5	93.8	98.3	100.3	103.1	100.0																		
**A-102**	104.4	102.6	106.9	103.5	107.1	103.5																		
**A-103**																								
**A-104**	101.1	99.5	104.5	102.2	101.2	100.6																		
**A-105**	74.1	91.0	32.7	85.6	93.5	94.7	98.5	87.4	96.6	96.4	89.8	97.1	93.7	97.4	93.1	93.9	97.9	103.0	97.3	74.7	97.1	99.8	98.2	99.6
**A-106**	103.4	103.1	100.2	100.5	102.1	100.8																		
**A-107**	107.0	104.9	106.6	107.8	106.3	107.5																		
**A-108**																								
**A-109**	99.3	97.6	98.0	97.2	100.4	98.5																		
**A-110**	97.0	93.8	91.0	92.6	92.9	95.2																		
**A-111**	93.1	92.6	95.9	92.3	93.7	92.6																		
**A-112**	81.9	80.0	80.8	83.4	91.2	84.4																		
**A-113**	100.2	101.3	102.0	101.8	102.4	104.0																		
**A-114**	105.4	104.8	107.2	104.7	106.7	101.3																		
**A-115**	103.2	106.2	100.0	101.6	110.1	104.3																		
**B-101**																								
**B-102**	92.9	96.5	94.1	94.3	96.2	91.5																		
**B-103**	77.4	87.9	88.2	89.6	88.2	87.5	90.0	84.3	89.4	88.1	89.4	88.7												
**B-104**	6.2	85.8	89.6	96.5	91.9	99.8	94.5	73.2	89.2	102.7	101.1	94.4	91.8	64.3	73.5	59.0	0.6	0.6	59.6	10.2	99.6	95.2	88.6	96.6
**B-105**																								
**B-106**	100.7	91.8	87.9	85.8	82.2	85.9																		
**B-107**	86.6	89.4	88.6	90.2	89.6	83.4																		
**B-108**	104.9	105.2	106.1	106.6	103.6	107.2																		
**B-109**	100.6	104.1	106.1	107.6	107.1	105.5																		
**B-110**	90.7	90.4	104.2	88.4	91.1	81.2																		
**B-111**	85.8	87.2	87.6	89.8	88.3	86.9																		
**B-112**	87.7	89.5	91.6	90.4	88.2	87.1																		
**B-113**	88.7	88.3	81.4	87.7	88.9	87.3																		
**B-114**	88.1	91.4	91.6	90.6	95.1	77.7	91.8	89.6	95.2	94.7	91.2	94.6												
**B-115**	93.3	92.6	93.6	91.5	91.3	93.7																		
**B115-2**	89.6	89.2	90.3	88.5	85.0	91.3																		

**C-101**																								
**C-102**	40.3	99.1	92.6	98.3	65.7	93.8	53.0	94.9	58.9	89.5	70.6	95.4	97.7	70.6	49.9	32.5	96.1	63.1	99.1	95.7	88.8	94.7	93.6	70.0
**C-103**	103.2	107.4	102.7	104.0	102.0	68.6	105.5	105.1	100.2	106.2	101.0	103.5												
**C-104**	95.6	95.6	96.2	96.3	94.6	94.5																		
**C-105**	92.7	91.7	93.1	92.7	92.9	93.0																		
**C-106**	94.1	96.1	97.0	99.2	97.6	95.2																		
**C-107**	90.2	87.7	93.8	91.9	91.6	93.1																		
**C-108**	98.8	98.9	100.8	96.0	100.3	94.9																		
**C-109**	93.4	95.1	94.4	93.9	94.1	95.3																		
**C-110**	103.7	104.3	103.8	102.4	104.2	100.3																		
**C-111**	96.6	64.1	94.1	93.1	82.5	95.8	90.8	90.5	91.7	93.9	89.6	92.3												
**C-112**	95.3	97.7	94.4	95.1	97.5	96.2																		
**C-113**	93.4	98.5	97.1	93.6	96.0	98.3																		
**C-114**	97.6	97.2	96.3	98.7	93.5	95.4																		
**C-115**	95.5	96.0	94.9	92.3	94.2	93.9																		
**D-101**	95.7	95.5	94.2	94.5	95.5	96.0																		
**D-102**	91.7	89.6	92.5	90.6	90.1	91.4																		
**D-103**	106.3	95.9	98.9	99.5	98.0	101.6																		
**D-104**	106.0	99.4	96.0	104.0	80.2	103.0																		
**D-105**	90.2	92.8	90.6	88.3	90.5	91.2																		
**D-106**	92.7	94.1	98.9	94.1	96.1	95.8																		
**D-107**	98.4	101.1	95.4	97.3	91.9	94.2																		
**D-108**	99.5	104.3	77.4	100.7	90.3	89.0	42.8	99.6	95.6	97.2	100.6	100.5	100.7	94.7	97.2	94.1	96.7	100.1	100.3	98.4	100.0	101.9	97.0	82.1
**D-109**	99.8	101.2	100.8	100.3	102.3	102.9																		
**D-110**	98.7	106.8	82.4	102.4	103.7	100.3																		
**D-111**	96.6	92.7	100.2	87.8	101.0	92.6																		
**D-112**																								
**D-113**	90.3	92.0	94.1	95.1	93.2	90.8																		
**D-114**	110.0	99.1	104.3	97.0	99.8	100.0																		
**D-115**	108.3	99.2	99.2	100.6	104.2	104.5																		

Yellow colour: Q is between Q_{Test}+5% and Q_{Test}. Orange colour: Q is between Q_{Test} and Q_{Test}-15%. Red colour: Q is between Q_{Test}-15% and Q_{Test}-25%. Dark red colour: Q is below Q_{Test}-25%.

Supplementary Table S14b: ESM content of individual units (C) tested in the uniformity of dosage units test (in % of the declared content)

	First stage										Second stage																			
**Sample ID**	**C1**	**C2**	**C3**	**C4**	**C5**	**C6**	**C7**	**C8**	**C9**	**C10**	**C1**	**C2**	**C3**	**C4**	**C5**	**C6**	**C7**	**C8**	**C9**	**C10**	**C11**	**C12**	**C13**	**C14**	**C15**	**C16**	**C17**	**C18**	**C19**	**C20**
**A-101**	96	92	93	90	95	93	90	94	97	94																				
**A-102**	98	100	103	103	99	100	100	104	106	100																				
**A-103**	95	94	95	98	94	99	94	96	97	99																				
**A-104**	103	107	108	101	104	103	102	107	105	104																				
**A-105**	100	98	97	103	102	101	104	99	103	98																				
**A-106**	108	104	100	107	106	102	101	103	101	101																				
**A-107**	105	108	107	107	107	100	108	109	107	100																				
**A-108**	91	95	92	88	87	87	88	90	92	93																				
**A-109**	103	107	104	103	103	106	104	105	104	107																				
**A-110**	103	102	103	106	96	104	104	101	104	102																				
**A-111**	108	105	105	103	105	103	107	104	105	94																				
**A-112**	106	108	112	109	112	109	109	109	111	108																				
**A-113**	103	102	98	95	97	101	102	99	99	101																				
**A-114**	106	107	111	103	102	100	106	103	112	107																				
**A-115**	108	106	100	108	108	104	107	105	109	111																				
**B-101**	103	99	100	100	97	98	96	100	101	100																				
**B-102**	97	93	96	93	95	94	90	92	93	94																				
**B-103**	82	86	89	91	89	85	85	89	88	85																				
**B-104**	104	93	92	94	93	88	98	94	101	94																				
**B-105**	93	89	95	93	94	94	96	92	95	94																				
**B-106**	98	93	90	91	95	99	86	99	92	88	96	96	91	92	94	96	94	93	95	95	104	99	99	93	97	95	96	99	98	96
**B-107**	78	85	90	86	83	84	84	79	79	82																				
**B-108**	110	106	105	105	108	104	103	106	99	107																				
**B-109**	108	107	111	101	107	107	103	101	106	105																				
**B-110**	101	94	96	87	97	91	100	88	92	98	103	93	88	96	95	82	88	95	88	92	99	98	94	92	97	88	88	91	96	93
**B-111**	90	94	90	89	84	86	89	86	87	89	90	80	92	87	92	91	88	86	88	92	90	87	87	81	88	86	88	91	89	82
**B-112**	93	94	105	97	99	93	103	97	102	104																				
**B-113**	107	105	98	105	100	98	98	98	100	99																				
**B-114**	93	95	94	97	100	96	95	100	98	97																				
**B-115**	104	99	95	96	97	93	97	98	97	84																				

**B115-2**	92	91	92	93	85	87	90	93	91	89
**C-101**	97	96	94	94	96	94	94	94	92	93
**C-102**	102	96	96	98	96	96	96	94	96	97
**C-103**	99	109	103	103	100	107	108	100	99	101
**C-104**	96	93	96	95	99	97	96	95	98	94
**C-105**	93	91	92	89	92	90	92	91	90	92
**C-106**	99	98	98	97	97	97	98	100	100	98
**C-107**	90	91	92	93	90	89	89	87	89	91
**C-108**	97	97	100	99	101	97	92	99	100	95
**C-109**	97	98	99	101	98	96	95	95	98	97
**C-110**	92	93	93	101	93	92	93	90	94	92
**C-111**	90	89	89	89	91	94	89	95	92	89
**C-112**	91	90	89	90	89	90	93	89	91	93
**C-113**	93	91	91	92	91	91	91	90	93	91
**C-114**	95	97	94	96	92	93	93	94	93	96
**C-115**	90	94	90	87	96	96	92	94	95	96
**D-101**	87	92	95	92	95	93	95	92	95	96
**D-102**	90	89	90	90	91	90	93	92	90	93
**D-103**	95	95	99	97	95	95	95	90	93	99
**D-104**	90	103	99	92	97	93	89	96	97	94
**D-105**	94	91	95	91	92	93	91	90	93	91
**D-106**	91	94	94	95	99	98	96	96	94	95
**D-107**	92	95	90	92	85	89	91	94	93	95
**D-108**	96	100	92	96	96	93	93	97	95	93
**D-109**	104	103	99	110	107	102	104	102	102	102
**D-110**	100	100	102	103	103	99	99	99	99	100
**D-111**	95	102	99	101	98	99	94	103	99	104
**D-112**	99	95	98	101	92	98	95	95	96	101
**D-113**	95	99	99	98	97	100	99	96	99	101
**D-114**	109	105	106	105	101	101	108	105	112	109
**D-115**	100	98	99	103	102	103	96	99	101	106

Yellow colour: C is between 0.85 × M_{ref} (= lower value 1) and 90% or between 110% and 1.15 × M_{ref} (= higher value 1).
Red colour: C is between 0.75 × M_{ref} (= lower value 2) and 0.85 × M_{ref} (= lower value 1).

Supplementary Table S15a: LST dissolution rate of individual units (Q) tested in the dissolution test (in % of the declared content)

	First stage						Second stage																		
**Sample ID**	**Q1**	**Q2**	**Q3**	**Q4**	**Q5**	**Q6**	**Q1**	**Q2**	**Q3**	**Q4**	**Q5**	**Q6**	**Q1**	**Q2**	**Q3**	**Q4**	**Q5**	**Q6**	**Q7**	**Q8**	**Q9**	**Q10**	**Q11**	**Q12**
**A-201**	48.6	50.3	45.0	42.8	59.3	61.4	42.7	42.8	45.6	46.4	48.6	49.2												
**A-202**	73.5	62.2	76.0	72.8	86.5	68.0	68.5	75.2	71.0	76.1	69.3	61.6	72.2	63.0	66.9	64.0	66.3	60.1	70.3	66.1	67.2	53.7	68.9	60.1
**A-203**	98.9	102.1	100.6	102.9	93.2	97.7																		
**A-204**	92.7	85.1	86.0	86.7	78.0	88.7	93.2	77.2	90.0	86.1	82.7	89.7												
**A-205**	94.1	93.1	93.3	83.7	94.9	86.1																		
**A-206**	97.3	100.2	97.5	95.6	97.2	94.5																		
**A-207**	92.1	94.8	91.7	90.6	92.4	89.1																		
**A-208**	89.9	80.8	86.4	80.1	88.3	84.6																		
**A-209**	61.9	61.3	65.4	74.1	60.2	61.1	69.1	71.0	59.1	65.1	58.6	52.3												
**A-210**	49.6	26.7	47.9	40.7	50.1	52.8	40.8	45.8	42.6	52.4	45.1	40.5												
**A-211**	93.2	89.9	77.9	85.5	88.9	77.9	90.1	90.0	92.6	93.4	90.4	81.7												
**A-212**	100.4	96.8	83.0	83.3	85.0	81.5																		
**A-213**	30.3	40.5	38.2	42.8	39.4	42.3	50.8	42.1	52.4	43.8	43.0	44.7												
**A-214**	113.2	111.5	109.4	106.8	105.1	104.9																		
**A-215**	103.6	106.3	102.8	105.1	103.2	109.8																		
**B-201**	112.2	111.1	114.3	112.5	115.3	110.9																		
**B-202**	105.1	103.0	104.0	106.8	104.2	106.5																		
**B-203**	41.4	42.2	36.2	54.5	61.4	55.7	39.9	39.9	41.6	39.9	36.3	36.9												
**B-204**	101.9	105.9	100.5	104.6	95.9	103.8																		
**B-205**	106.5	109.0	108.8	98.0	107.1	108.5																		
**B-206**	100.9	102.0	107.6	96.7	99.4	104.5																		
**B-207**	41.0	41.4	46.0	45.4	42.0	41.5	43.4	40.6	43.3	40.3	44.2	28.4												
**B-208**	86.9	101.6	87.6	94.9	88.3	99.0																		
**B-209**	103.3	99.1	102.7	103.8	96.4	103.1																		
**B-210**	94.9	83.0	78.7	99.0	97.0	91.0	96.9	97.1	85.3	94.1	88.5	65.2												
**B-211**	37.6	38.3	38.7	42.8	40.2	39.0	40.5	34.1	38.9	38.9	45.2	34.8												
**B-212**	101.5	100.8	101.9	99.8	101.9	95.5																		
**B-213**	98.2	81.9	97.3	95.8	92.9	90.8																		
**B-214**	98.5	98.9	99.5	100.9	98.9	99.2																		

**B-215**	41.0	46.1	44.9	40.1	42.8	45.7	40.0	45.6	40.7	40.4	43.1	42.7												
**C-201**	93.9	95.9	96.4	95.7	99.5	95.9																		
**C-202**	104.7	103.9	101.1	100.1	101.1	99.4																		
**C-203**	104.3	105.7	108.7	103.3	101.3	102.1																		
**C-204**	94.4	96.5	95.9	93.6	101.7	96.9																		
**C-205**	97.1	92.4	97.2	98.1	93.6	96.5																		
**C-206**	42.5	37.4	42.0	40.0	38.5	45.7	38.1	38.3	39.6	40.2	35.4	40.1												
**C-207**	51.5	65.0	68.1	68.6	60.9	61.1	62.6	63.6	74.7	61.8	65.5	70.1	78.4	61.3	80.8	65.9	75.5	67.1	60.1	69.3	95.7	70.8	76.6	64.6
**C-208**	86.0	83.7	76.8	74.7	77.0	67.2	75.3	75.2	89.5	68.8	73.5	58.9	89.6	92.0	84.9	87.7	72.3	78.2	71.4	85.9	75.4	71.2	75.1	73.0
**C-209**	94.2	95.3	91.8	96.6	96.1	96.2																		
**C-210**	96.4	95.4	99.4	100.7	94.9	97.5																		
**C-211**	93.3	99.4	93.5	100.7	93.9	96.4																		
**C-212**	67.2	74.2	67.3	67.9	68.8	73.2	69.9	70.3	77.0	71.0	69.2	76.6	78.2	77.0	74.1	73.3	78.9	71.4	81.3	66.7	75.2	73.3	77.7	76.7
**C-213**	96.4	97.9	101.8	97.3	96.2	96.0																		
**C-214**	97.0	99.5	99.7	98.6	101.5	100.2																		
**C-215**	96.0	99.1	100.0	98.7	87.2	95.4																		
**D-201**	67.8	65.9	69.3	95.1	90.9	66.5	62.7	62.4	82.5	70.4	84.1	97.3												
**D-202**	95.4	100.2	96.5	101.3	99.0	95.3																		
**D-203**	100.7	101.3	101.0	104.4	105.3	102.0																		
**D-204**	98.8	98.3	99.7	101.6	98.3	100.5																		
**D-205**	99.8	98.2	98.0	84.5	99.1	97.8																		
**D-206**	99.1	98.2	100.0	99.3	97.8	102.8																		
**D-207**	98.7	87.5	95.4	95.6	98.7	100.4																		
**D-208**	97.4	81.9	91.0	80.7	81.5	95.6																		
**D-209**	102.1	103.5	93.2	105.4	105.1	96.7																		
**D-210**	110.2	111.7	110.3	109.0	111.1	110.6																		
**D-211**	109.2	108.5	106.7	107.5	104.0	106.4																		
**D-212**	105.5	99.8	103.1	99.1	105.3	87.5																		
**D-213**	40.6	38.1	45.0	39.3	34.8	43.4																		
**D-214**	109.8	108.6	109.1	105.3	105.5	105.3																		
**D-215**	102.6	104.6	97.8	98.6	105.3	100.5																		

Yellow colour: Q is between Q_{Test}+5% and Q_{Test}. Orange colour: Q is between Q_{Test} and Q_{Test}-15%.
Red colour: Q is between Q_{Test}-15% and Q_{Test}-25%. Dark red colour: Q is below Q_{Test}-25%.

Supplementary Table S15b: LST content of individual units (C) tested in the uniformity of dosage units test (in % of the declared content)

	**First stage**										**Second stage**																			
**Sample ID**	**C1**	**C2**	**C3**	**C4**	**C5**	**C6**	**C7**	**C8**	**C9**	**C10**	**C1**	**C2**	**C3**	**C4**	**C5**	**C6**	**C7**	**C8**	**C9**	**C10**	**C11**	**C12**	**C13**	**C14**	**C15**	**C16**	**C17**	**C18**	**C19**	**C20**
**A-201**	104	99	101	98	100	105	102	95	102	101																				
**A-202**	98	98	94	94	99	98	103	99	97	100																				
**A-203**	101	93	94	94	95	95	97	95	97	94																				
**A-204**	100	95	98	98	98	97	95	100	103	98																				
**A-205**	105	107	98	107	101	101	102	103	103	100																				
**A-206**	103	101	106	104	109	106	105	99	102	101																				
**A-207**	100	97	102	101	97	100	93	100	95	95																				
**A-208**	97	101	100	100	99	97	101	97	97	93																				
**A-209**	106	100	106	103	104	107	105	104	107	105																				
**A-210**	102	101	102	99	102	100	101	100	96	105																				
**A-211**	99	97	97	99	102	99	97	96	99	100																				
**A-212**	100	98	97	94	99	99	101	96	93	98																				
**A-213**	101	99	103	104	99	105	99	100	101	96																				
**A-214**	100	102	103	101	97	99	97	97	95	100																				
**A-215**	98	101	98	97	99	95	98	99	94	95																				
**B-201**	100	97	101	100	96	97	95	101	98	100																				
**B-202**	97	98	100	101	101	99	101	99	101	103																				
**B-203**	103	100	101	96	104	95	95	101	103	95																				
**B-204**	96	93	94	95	96	95	99	94	95	98																				
**B-205**	100	101	103	99	98	100	100	101	99	99																				
**B-206**	103	101	100	102	103	101	100	100	101	100																				
**B-207**	99	99	98	102	101	100	96	99	97	99																				
**B-208**	98	94	91	90	94	91	92	92	90	97																				
**B-209**	96	102	97	98	94	100	98	97	99	96																				
**B-210**	99	97	98	100	99	100	101	99	99	99																				
**B-211**	97	95	96	100	96	95	101	100	100	98																				
**B-212**	99	100	103	98	99	99	100	100	97	101																				
**B-213**	98	100	99	101	98	97	99	100	99	95																				
**B-214**	99	99	98	99	99	96	98	98	99	100																				

**B-215**	99	94	96	95	97	96	98	102	96	100																				
**C-201**	95	97	94	89	91	95	92	94	91	93																				
**C-202**	103	98	99	98	99	91	100	96	97	100																				
**C-203**	96	101	99	97	96	97	101	97	96	100																				
**C-204**	96	95	95	99	99	95	95	96	95	98																				
**C-205**	96	92	92	96	94	96	98	97	96	95																				
**C-206**	97	91	96	92	94	90	94	93	93	94																				
**C-207**	97	106	92	93	88	102	106	99	88	107	91	92	110	94	97	111	113	108	97	112	107	89	112	110	110	90	109	104	111	111
**C-208**	99	97	96	97	106	100	95	104	97	93																				
**C-209**	89	91	92	89	93	90	94	91	91	90																				
**C-210**	97	97	96	94	102	96	93	95	93	94																				
**C-211**	94	94	94	96	95	99	98	99	102	96																				
**C-212**	100	99	99	99	97	97	95	98	97	96																				
**C-213**	94	88	91	95	94	95	91	88	90	87																				
**C-214**	96	97	94	97	98	94	97	92	92	94																				
**C-215**	101	95	98	100	95	97	93	95	95	97																				
**D-201**	97	96	99	95	94	96	97	93	95	93																				
**D-202**	101	102	95	93	98	95	99	98	96	96																				
**D-203**	96	97	99	98	98	94	102	100	98	97																				
**D-204**	99	105	97	96	97	98	100	99	100	99																				
**D-205**	98	98	96	98	99	96	95	98	96	94																				
**D-206**	97	100	99	103	104	98	97	96	100	98																				
**D-207**	93	93	91	93	92	94	94	90	94	90																				
**D-208**	98	99	98	97	97	99	97	102	96	100																				
**D-209**	97	98	97	95	100	99	97	98	92	98																				
**D-210**	101	97	96	101	98	99	99	100	100	99																				
**D-211**	97	97	101	99	96	97	99	101	98	97																				
**D-212**	96	94	92	92	93	97	89	95	97	95																				
**D-213**	95	95	95	94	94	97	97	97	92	94																				
**D-214**	99	96	94	97	96	96	94	98	98	96																				
**D-215**	94	96	97	93	92	94	92	92	91	95																				

Yellow colour: C is between 0.85 × M_{ref} (= lower value 1) and 90% or between 110% and 1.15 × M_{ref} (= higher value 1).

Supplementary Material 6: Representative HPLC Chromatogram Figures

Supplementary Fig. S1: Dissolution test chromatograms of the AZM standard product and sample D-405 (Tablet 5, 1.S; AZM tablets).

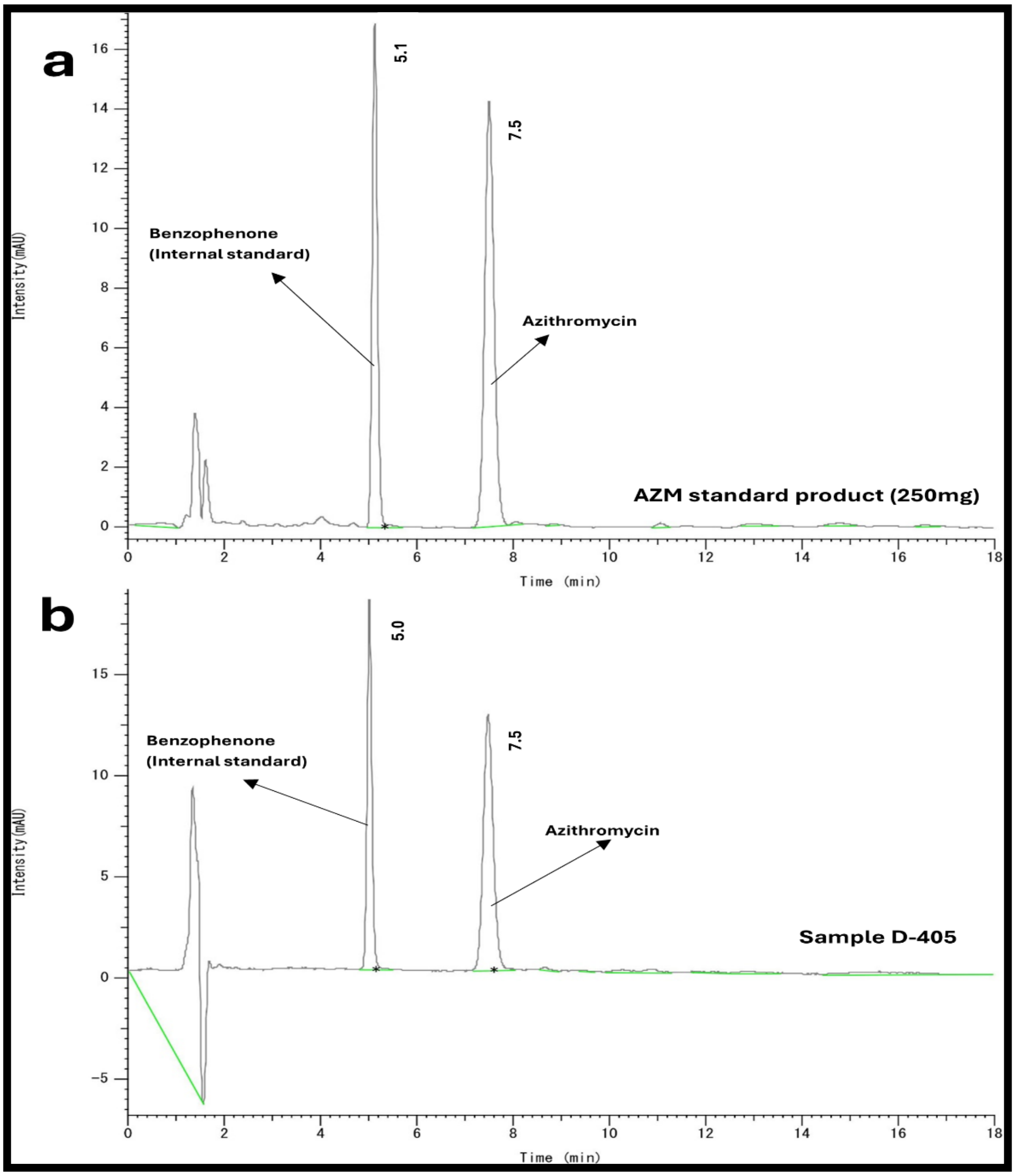

Legend: Supplementary Fig. S1 shows selected chromatograms of dissolution tests of (a) the AZM standard product 250 mg and (b) sample D-405 Tablet 5, 1.S ($Q < Q_{Test}$-25%), both obtained under AZM HPLC conditions. The labelling of D-405 indicated a dosage strength of 500 mg. An image of (b) is shown in Fig. 4. 1.S = First stage of the dissolution test.

Supplementary Fig. S2: Dissolution test chromatograms of the ESM standard product and sample A-105 (ESM tablets), which is representative for the failing units of samples A-105, C-102 and D-108.

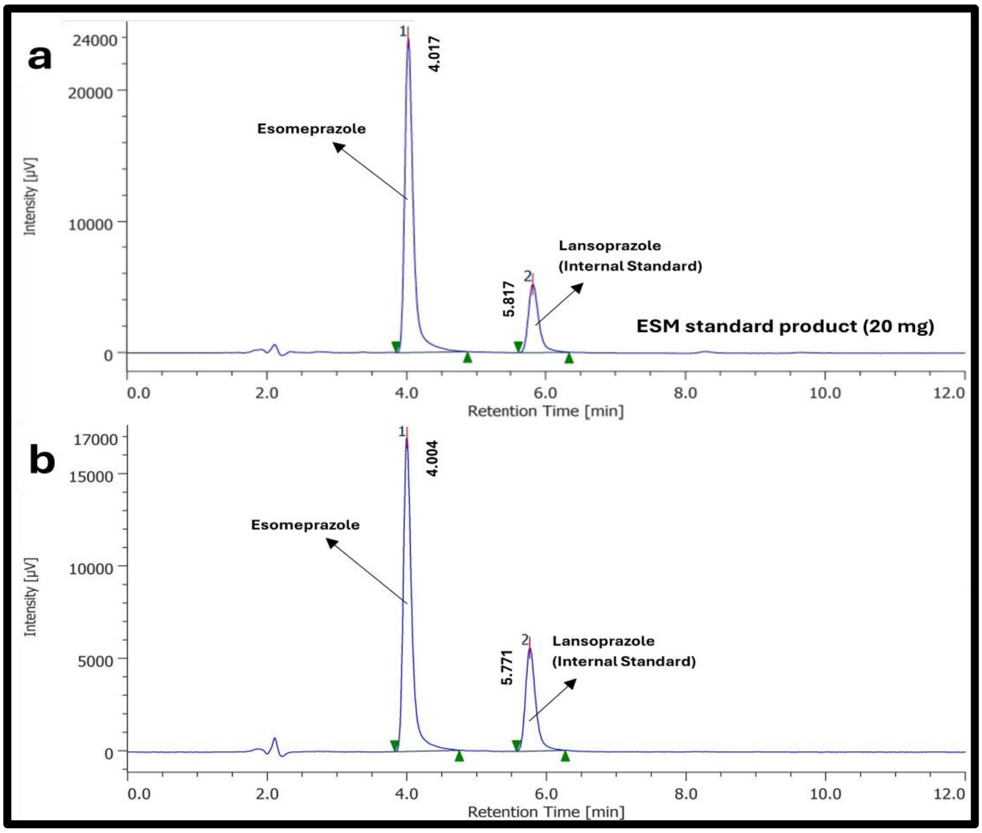

Legend: Supplementary Fig. S2 shows selected chromatograms of dissolution tests of (a) the ESM standard product 20 mg and (b) sample A-105 (ESM tablets), both obtained under ESM HPLC conditions. (b) is representative for the failing individual units of samples A-105, C-102 and D-108 ($Q < Q_{Test}$-25%). The labelling of the ESM samples indicated a dosage strength of 40 mg.

Supplementary Fig. S3: Dissolution test chromatograms of individual units of sample B-104 (ESM tablets).

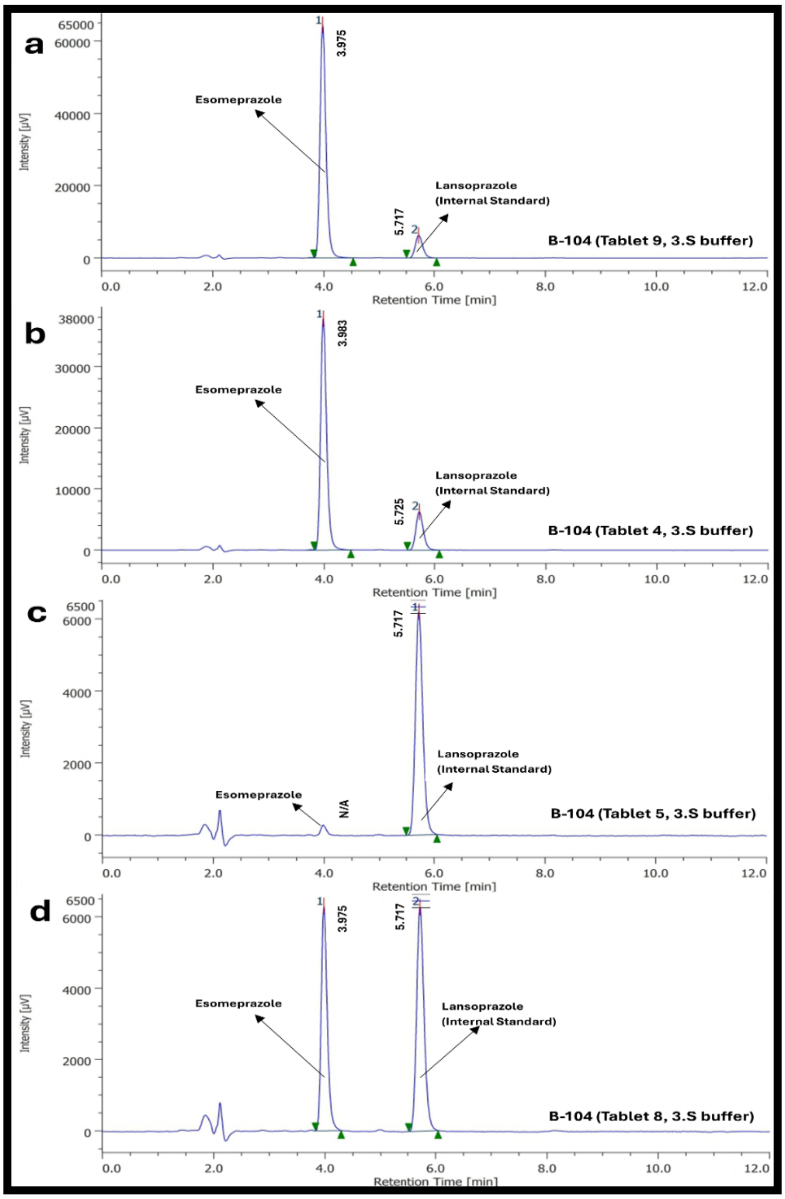

Legend: Supplementary Fig. S3 shows selected chromatograms of dissolution tests of sample B-104 (ESM tablets). (a) representative passing unit for comparison (Tablet 9, 3.S buffer), (b) Tablet 4, 3.S buffer ($Q < Q_{Test}$-15%), (c) Tablet 5, 3.S buffer ($Q < Q_{Test}$-25%) and (d) Tablet 8, 3.S buffer ($Q < Q_{Test}$-25%), all obtained under ESM HPLC conditions. In (c) the peak was not integrated due to the low signal. The labelling of the sample B-104 indicated a dosage strength of 40 mg. Images of (b)-(d) are shown in Fig. 5. 3.S = Third stage of the dissolution test.

Supplementary Fig. S4: Dissolution test chromatograms of the LST standard product, and samples B-215 and A-202 (LST tablets), which are representative for samples labelled with Batch 22 and Batch 11, and Batch 35, Batch 89 and AP Trade name 3, respectively.

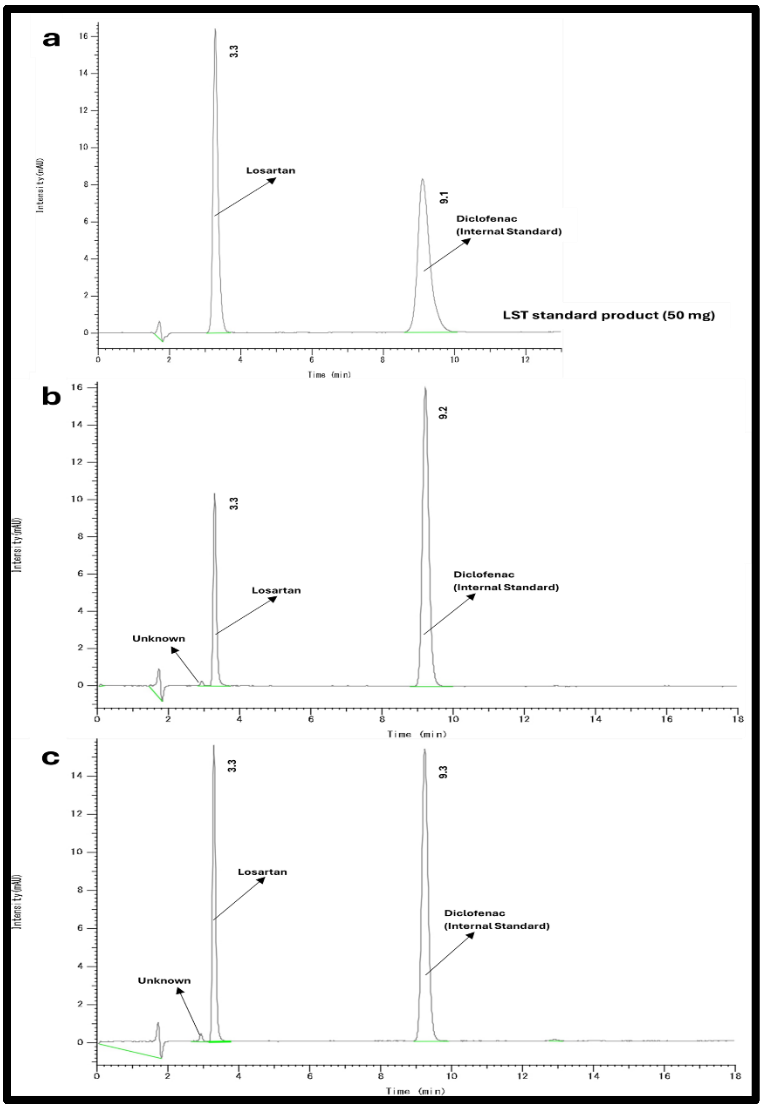

Legend: Supplementary Fig. S4 shows selected chromatograms of dissolution tests of (a) the LST standard product 50 mg, (b) sample B-215 (LST tablets), and (c) sample A-202 (LST tablets), all obtained under LST HPLC conditions. (b) is representative for the failing individual units of samples labelled with Batch 22 (A-201, A-210, A-213, and B-215) and Batch 11 (B-203, B-207, B-211, C-206, and D-213) ($Q < Q_{Test}$-25%). (c) is representative for the failing individual units of samples labelled with Batch 35 (A-202 and A-209), Batch 89 (C-212), and AP Trade name 3 ($Q < Q_{Test}$-15%). The labelling of the LST samples indicated a dosage strength of 50 mg. Images of (b) and (c) are shown in Fig. 6.

Supplementary Material 7: Price Analysis Data

Supplementary Table S16: Overview of the price data of sample prices per unit and Medicine Price Ratios (MPRs)

API	District / Dosage formulation	Median price/unit [US$]	Average price/unit [US$]	SD price/unit [US$]	SD%	Minimum price/unit [US$]	Maximum price/unit [US$]	25th percentile price/unit [US$]	75th percentile price/unit [US$]	Median MPR	Minimum MPR	Maximum MPR
AZM	**Total**	**0.24471**	**0.24471**	**0.00000**	**0.0%**	**0.24471**	**0.24471**	**0.24471**	**0.24471**	**1.079**	**1.079**	**1.079**
	SP	0.24471	0.24471	0.00000	0.0%	0.24471	0.24471	0.24471	0.24471	1.079	1.079	1.079
	KTM	0.24471	0.24471	0.00000	0.0%	0.24471	0.24471	0.24471	0.24471	1.079	1.079	1.079
CFIX	**Total**	**0.16314**	**0.15895**	**0.01488**	**9.4%**	**0.10074**	**0.18957**	**0.16314**	**0.16314**	**0.983**	**0.607**	**1.142**
	SP	0.16314	0.15970	0.01547	9.7%	0.11664	0.18957	0.16314	0.16314	0.983	0.703	1.142
	KTM	0.16314	0.15822	0.01425	9.0%	0.10074	0.16314	0.16314	0.16314	0.983	0.607	0.983
	Dispersible Tablets	0.16314	0.15275	0.01996	13.1%	0.10074	0.16314	N/A	N/A	0.983	0.607	0.983
	Tablets	0.16314	0.16237	0.00952	5.9%	0.13402	0.18957	N/A	N/A	0.983	0.807	1.142
ESM	**Total**	**0.08622**	**0.09463**	**0.02092**	**22.1%**	**0.07023**	**0.17456**	**0.08157**	**0.09515**	**N/A**	**N/A**	**N/A**
	SP	0.08157	0.09597	0.02142	22.3%	0.08157	0.13867	0.08157	0.12235	N/A	N/A	N/A
	KTM	0.08769	0.09324	0.02030	21.8%	0.07023	0.17456	0.08157	0.09229	N/A	N/A	N/A
	Capsules	0.08157	0.08157	0.00000	0.0%	0.08157	0.08157	N/A	N/A	N/A	N/A	N/A
	Tablets	0.08769	0.09632	0.02167	22.5%	0.07023	0.17456	N/A	N/A	N/A	N/A	N/A
LST	**Total**	**0.06199**	**0.06269**	**0.00272**	**4.3%**	**0.06118**	**0.07341**	**0.06199**	**0.06199**	**0.539**	**0.532**	**0.638**
	SP	0.06199	0.06326	0.00359	5.7%	0.06118	0.07341	0.06118	0.06240	0.539	0.532	0.638
	KTM	0.06199	0.06211	0.00061	1.0%	0.06199	0.06525	0.06199	0.06199	0.539	0.539	0.567

N/A = Not available / Not calculated
MPR = Medicine Price Ratio calculated as the ratio of the individual sample unit prices and the international reference unit prices
SP = Saptari district
KTM = Kathmandu district

Supplementary Fig. S5: Prices per unit of all AZM, CFIX, ESM, and LST samples and relevant international reference unit prices (IRPs).

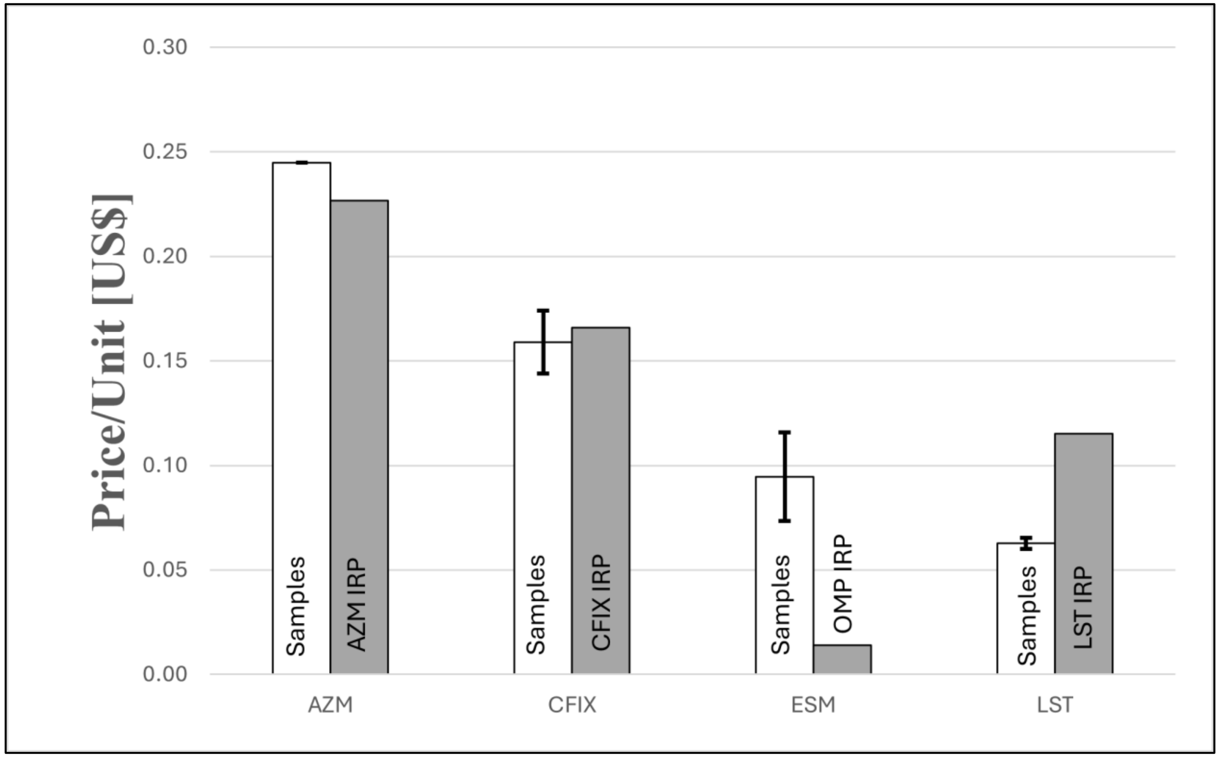

Legend: Supplementary Fig. S5 illustrates the prices per unit of the samples expressed as absolute US$ values, which were calculated as described in the Price analysis section, as well as the international reference unit prices (IRPs) for AZM, CFIX, omeprazole 20 mg tablets (OMP IRP), and LST.

Supplementary Fig. S6: Prices per unit of all samples, separated by the Saptari and Kathmandu districts.

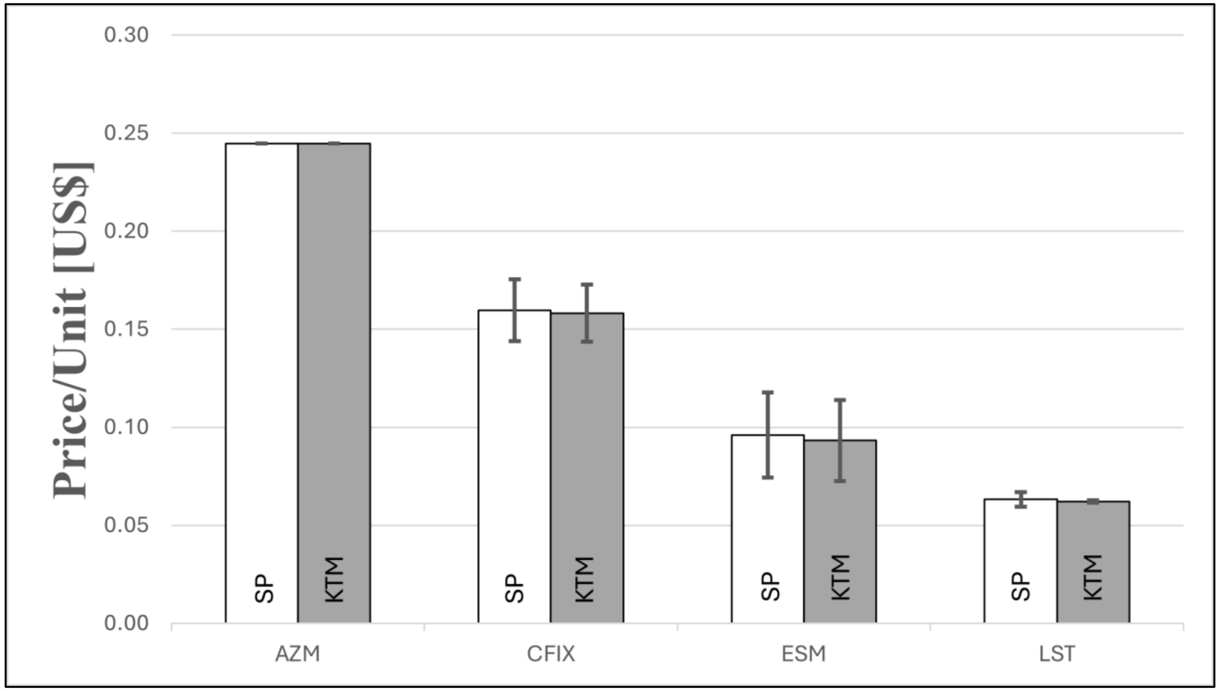

Legend: Supplementary Fig. S6 illustrates the prices per unit of the samples expressed as absolute US$ values, which were calculated as described in the Price analysis section, separated by the sampling regions Saptari district (SP) and Kathmandu district (KTM).

Supplementary Fig. S7: Prices per unit of all samples, separated by their dosage formulation.

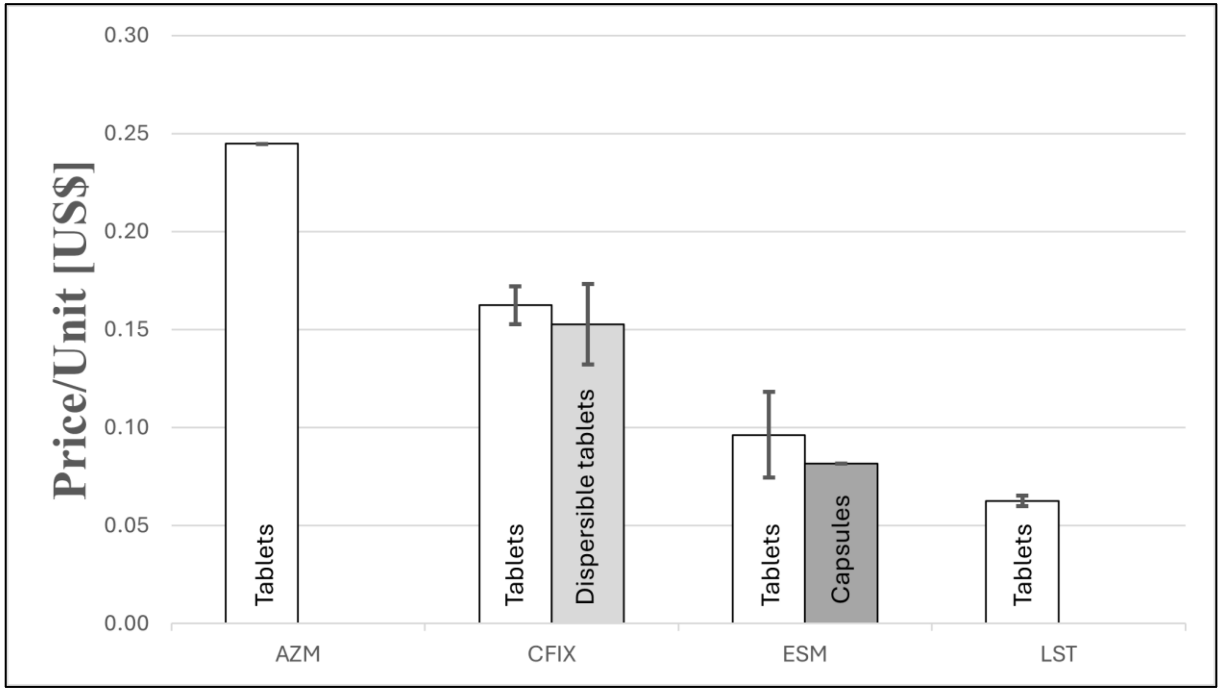

Legend: Supplementary Fig. S7 illustrates the prices per unit of all samples expressed as absolute US$ values, which were calculated as described in the Price analysis section, separated by their dosage formulation (capsules, dispersible tablets, and tablets).

Supplementary Material 8: Raman Spectra – Figures and Descriptions

Azithromycin Raman scattering analysis results

<u>AZM – C13560 analysis</u>:

Fifty-six the 60 AZM samples (93.3%) had a small characteristic peak near Raman shift wavenumber 1,452 cm^{-1} corresponding to the reference standard, whereas no such peak could be identified in the spectra of four samples (A-406, C-412, D-402, and D-403) using the C13560 ultra-compact Raman spectrometer.

Supplementary Fig. S8: Raman spectra of the AZM reference standard and all 60 AZM samples obtained using a C13560 ultra-compact Raman spectrometer.

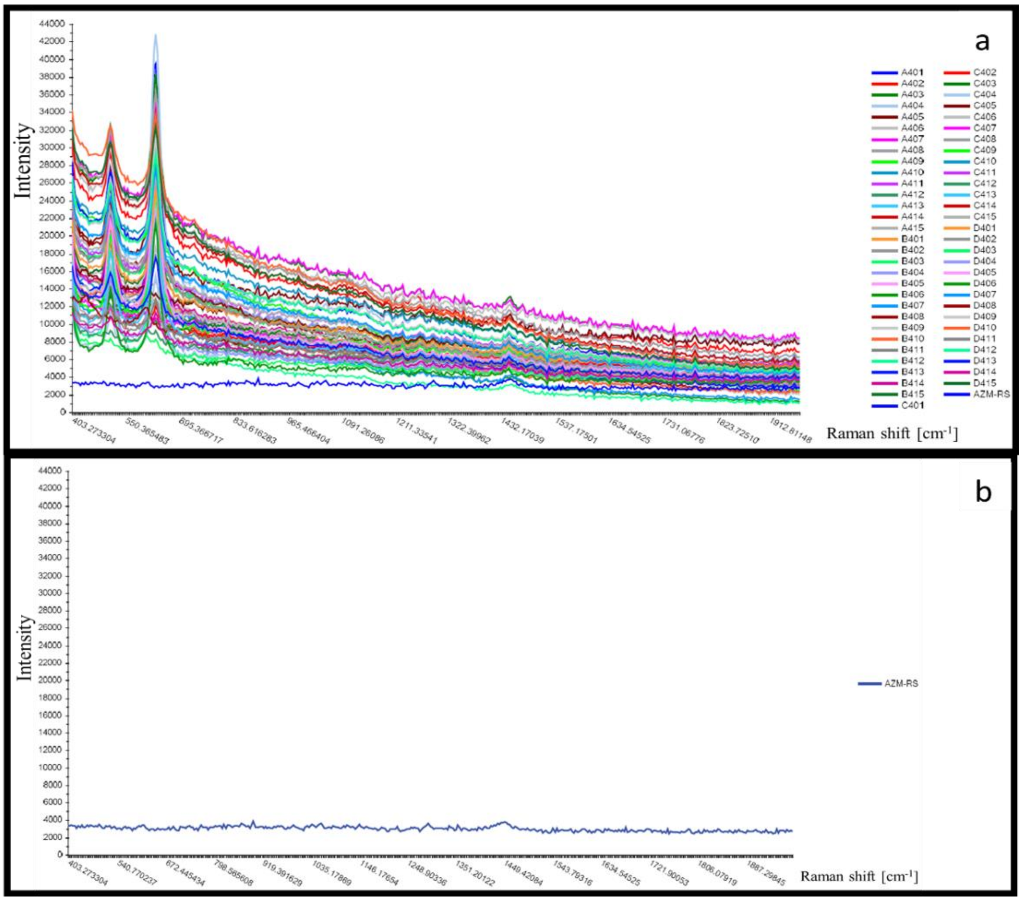

Legend: Supplementary Fig. S8 shows the line plot of the Raman spectra of a: all 60 AZM samples and the AZM reference standard (AZM-RS) and b: the AZM-RS only. Spectral data were obtained using an ultra-compact Raman spectrometer C13560. A total of 50 spectral data, scanned on five randomly selected positions each on the tablet upside and tablet

downside, were averaged each to obtain the spectra. The AZM-RS, available as powder, was placed in a plastic bag and piled up and scanned in the same manner as the tablets. A peak near a Raman shift of 1,452 cm^{-1} is visible in 56 of 60 spectra, whilst it is not visible in samples A-406, C-412, D-402 and D-403. The spectra are presented with colouring according to the sample and the AZM-RS.

<u>AZM – Inspector500 analysis</u>:

All of the 60 AZM samples (100%) showed a characteristic peak near Raman shift wavenumbers 1,460 cm^{-1} corresponding to the reference standard.

Supplementary Fig. S9: Raman spectra of the AZM reference standard and all 60 AZM samples obtained using an Inspector500 portable Raman spectrometer.

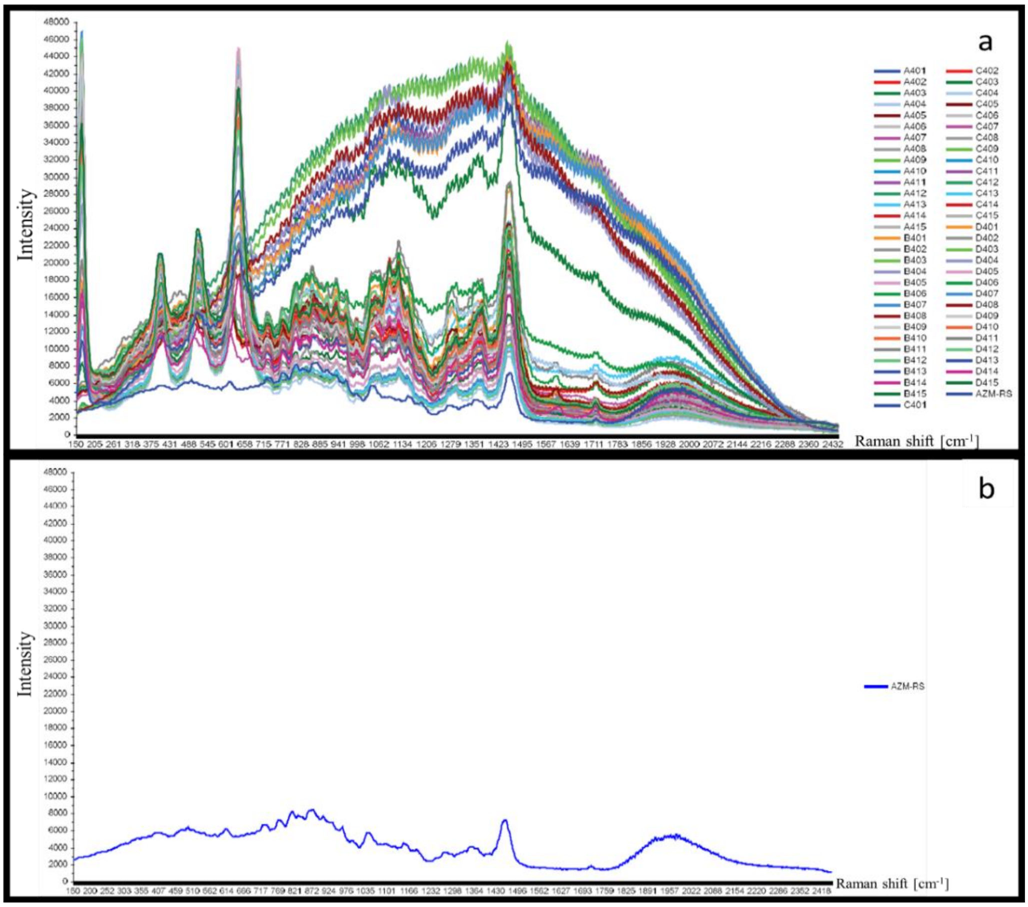

Legend: Supplementary Fig. S9 shows the line plot of the Raman spectra of a: all 60 AZM samples and the AZM reference standard (AZM-RS) and b: the AZM-RS only. Spectral data were obtained using a portable Raman spectrometer Inspector500. A total of 50 spectral data, scanned on five randomly selected positions each on the tablet upside and tablet downside, were averaged each to obtain the spectra. The AZM-RS, available as powder, was placed in a plastic bag and piled up and scanned in the same manner as the tablets. A peak near a Raman

shift of 1,460 cm^{-1} is visible in all 60 spectra. The spectra are presented with colouring according to the sample and the AZM-RS.

Cefixime Raman scattering analysis results

<u>CFIX – C13560 analysis</u>:

Fifty-six of the 60 CFIX samples (93.3%) showed characteristic peaks near Raman shift wavenumbers 1,296 cm^{-1}, 1,333 cm^{-1} and 1,610 cm^{-1} corresponding to the reference standard, whereas no such peak could be identified in the spectra of four samples (B-303, B-312, B-314, and C-301).

Supplementary Fig. S10: Line plot of the Raman spectra of all 60 CFIX samples and the CFIX reference standard obtained using the ultra-compact Raman spectrometer.

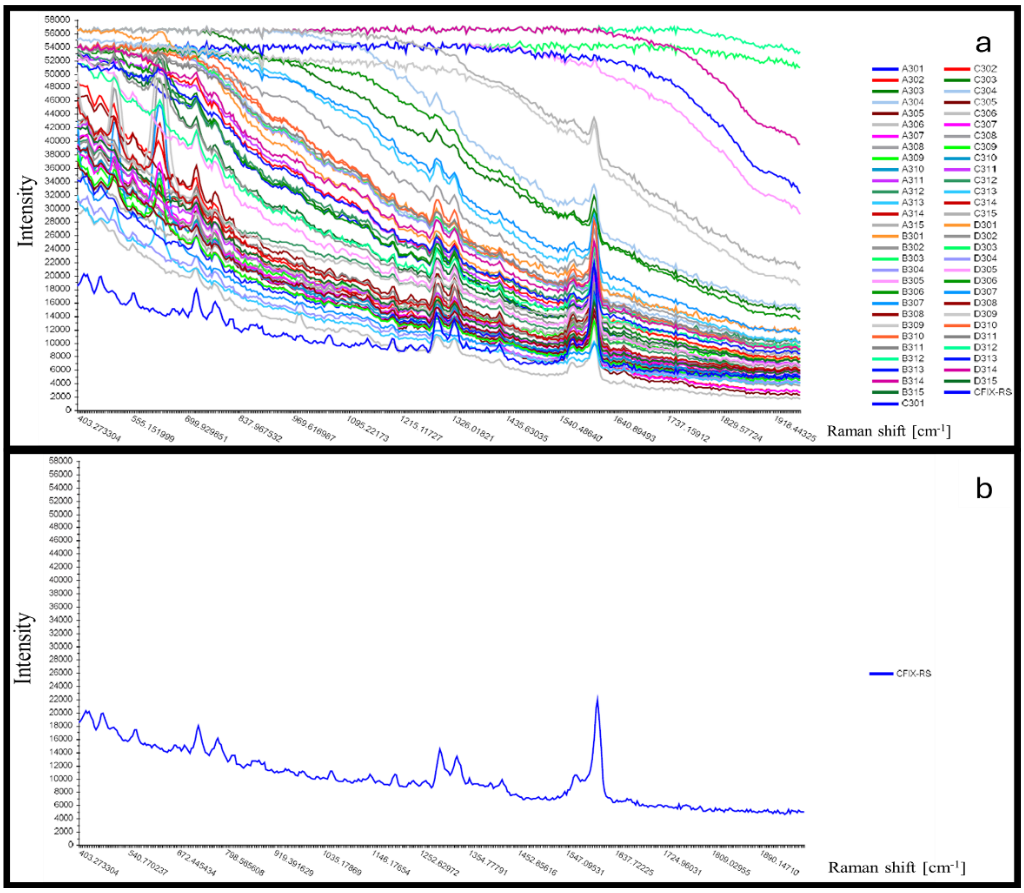

Legend: Supplementary Fig. S10 shows the line plot of the Raman spectra of a: all 60 CFIX samples and the CFIX reference standard (CFIX-RS) and b: the CFIX-RS only. Spectral data were obtained using an ultra-compact Raman spectrometer C13560. A total of 50 spectral data, scanned on five randomly selected positions each on the tablet upside and tablet downside, were averaged each to obtain the spectra. The CFIX-RS, available as powder, was placed in a plastic bag and piled up and scanned in the same manner as the tablets. Peaks near

Raman shifts of 1,296 cm^{-1}, 1,333 cm^{-1} and 1,610 cm^{-1} are visible in 56 of 60 spectra, whilst they are not visible in samples B-303, B-312, B-314, and C-301. The spectra are presented with colouring according to the sample and the CFIX-RS.

<u>CFIX – Inspector500 analysis</u>:

All of the 60 CFIX samples (100%) showed characteristic peaks near Raman shift wavenumbers 735 cm^{-1}, 785 cm^{-1}, 1,305 cm^{-1}, 1,340 cm^{-1}, and a strong peak near 1,615 cm^{-1}, corresponding to the reference standard.

Supplementary Fig. S11: Raman spectra of the CFIX reference standard and all 60 CFIX samples obtained using an Inspector500 portable Raman spectrometer.

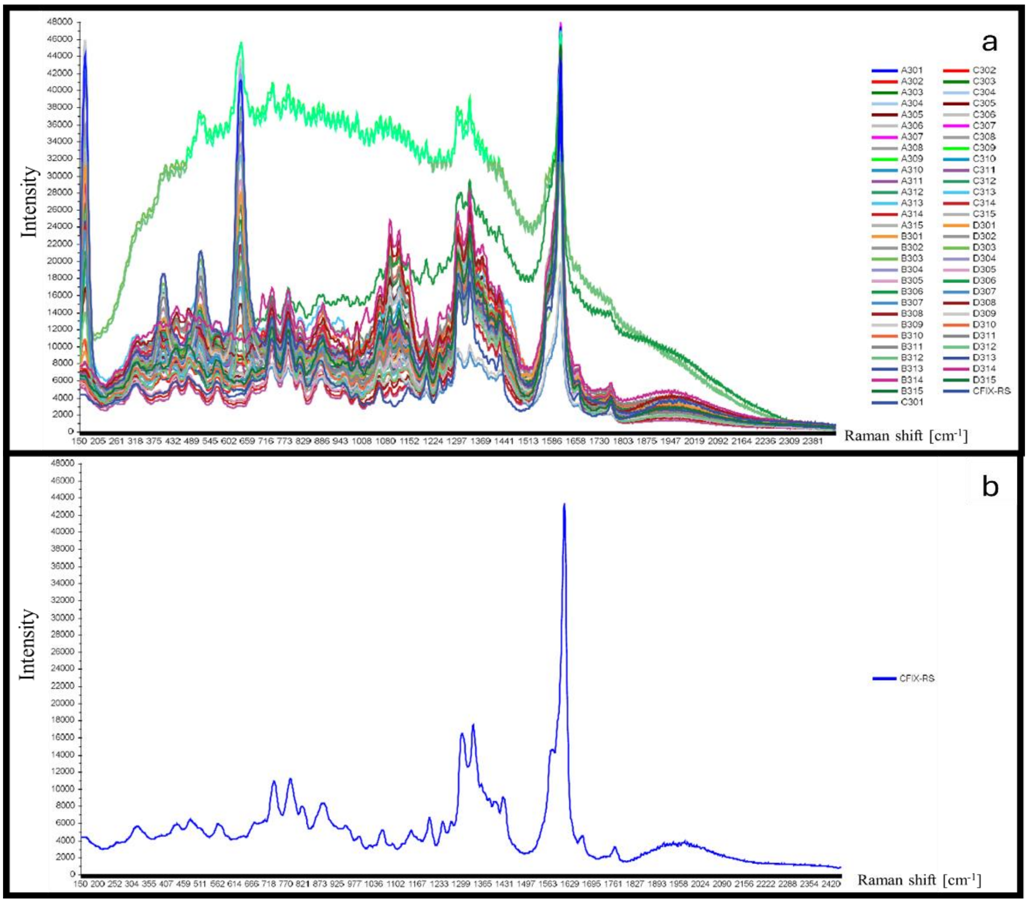

Legend: Supplementary Fig. S11 shows the line plot of the Raman spectra of a: all 60 CFIX samples and the CFIX reference standard (CFIX-RS) and b: the CFIX-RS only. Spectral data were obtained using a portable Raman spectrometer Inspector500. A total of 50 spectral data, scanned on five randomly selected positions each on the tablet upside and tablet downside, were averaged each to obtain the spectra. The CFIX-RS, available as powder, was placed in a plastic bag and piled up and scanned in the same manner as the tablets. Peaks near Raman shifts 735 cm^{-1}, 785 cm^{-1}, 1,305 cm^{-1}, 1,340 cm^{-1}, and a strong peak near 1,615 cm^{-1} are

visible in all 60 spectra. The spectra are presented with colouring according to the sample and the CFIX-RS.

Losartan Raman scattering analysis results

LST – C13560 analysis:
All of the 60 LST samples (100%) showed characteristic peaks near Raman shift wavenumbers 1,290 cm^{-1} and 1,605 cm^{-1} corresponding to the reference standard.

Supplementary Fig. S12: Raman spectra of the LST reference standard and all 60 LST samples obtained using a C13560 ultra-compact Raman spectrometer.

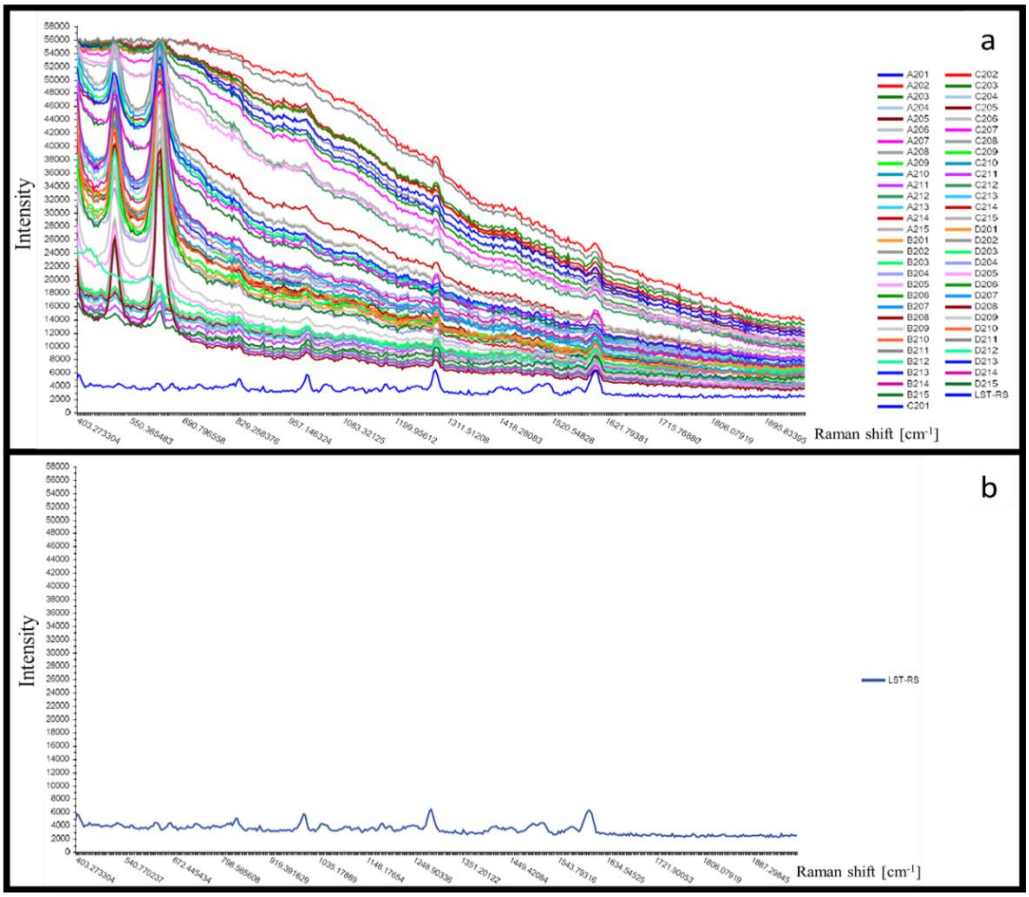

Legend: Supplementary Fig. S12 shows the line plot of the Raman spectra of a: all 60 LST samples and the LST reference standard (LST-RS) and b: the LST-RS only. Spectral data were obtained using an ultra-compact Raman spectrometer C13560. A total of 50 spectral data, scanned on five randomly selected positions each on the tablet upside and tablet downside, were averaged each to obtain the spectra. The LST-RS, available as powder, was placed in a plastic bag and piled up and scanned in the same manner as the tablets. Peaks near Raman shifts of 1,290 cm^{-1} and 1,605 cm^{-1} are visible in all 60 spectra. The spectra are presented with colouring according to the sample and the LST-RS.

LST – Inspector500 analysis:

All of the 60 LST samples (100%) showed characteristic peaks near Raman shift wavenumbers 840 cm^{-1}, 1,010 cm^{-1}, 1,296 cm^{-1} and a strong peak near 1,610 cm^{-1} corresponding to the reference standard.

Supplementary Fig. S13: Raman spectra of the LST reference standard and all 60 LST samples obtained using an Inspector500 portable Raman spectrometer.

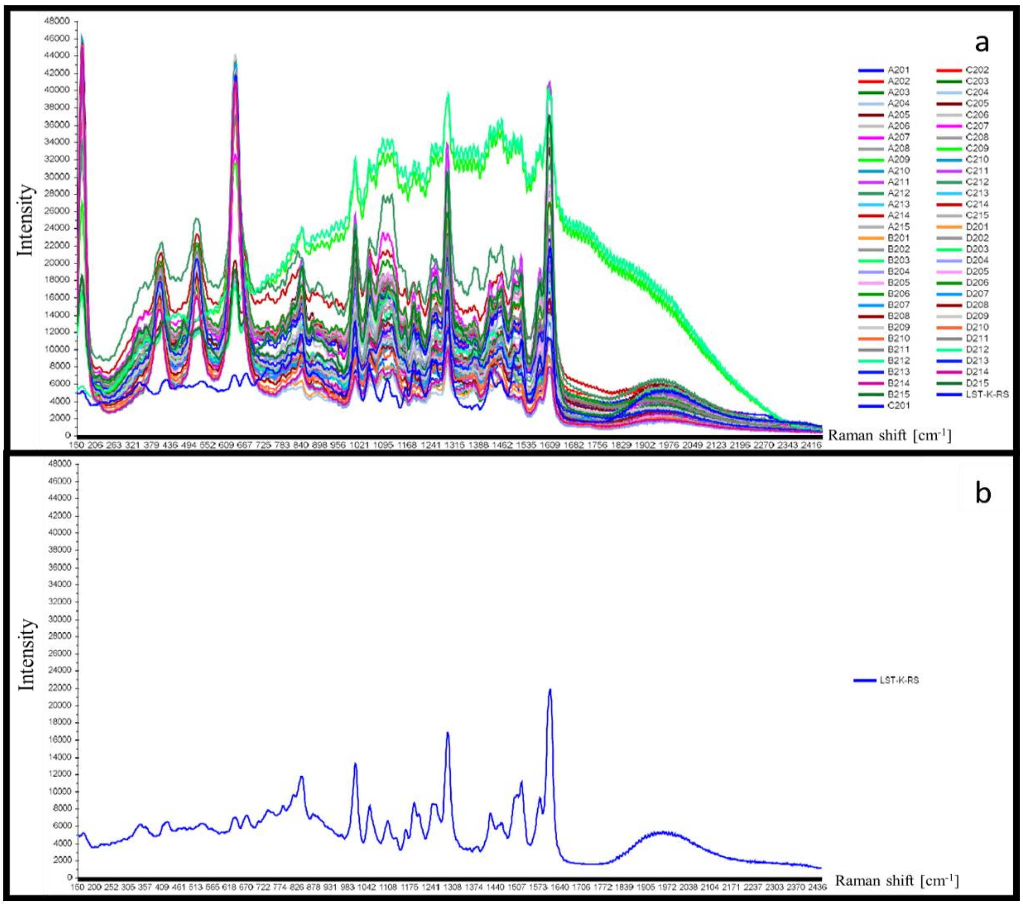

Legend: Supplementary Fig. S13 shows the line plot of the Raman spectra of a: all 60 LST samples and the LST reference standard (LST-RS) and b: the LST-RS only. Spectral data were obtained using a portable Raman spectrometer Inspector500. A total of 50 spectral data, scanned on five randomly selected positions each on the tablet upside and tablet downside, were averaged each to obtain the spectra. The LST-RS, available as powder, was placed in a plastic bag and piled up and scanned in the same manner as the tablets. Peaks near Raman shifts of 840 cm^{-1}, 1,010 cm^{-1}, 1,296 cm^{-1}, and a strong peak near 1,610 cm^{-1} are visible in all 60 spectra. The spectra are presented with colouring according to the sample and the LST-RS.

Esomeprazole Raman scattering analysis results

In comparison to the Raman peaks of the Omeprazole standard, none of the ESM samples showed corresponding peaks were visually identified using both spectrometers. Therefore, identification of ESM was not possible using the methods used in this study.

<u>ESM – C13560 analysis</u>:

Supplementary Fig. S14: Raman spectra of the Omeprazole reference standard and all 60 ESM samples obtained using a C13560 ultra-compact Raman spectrometer.

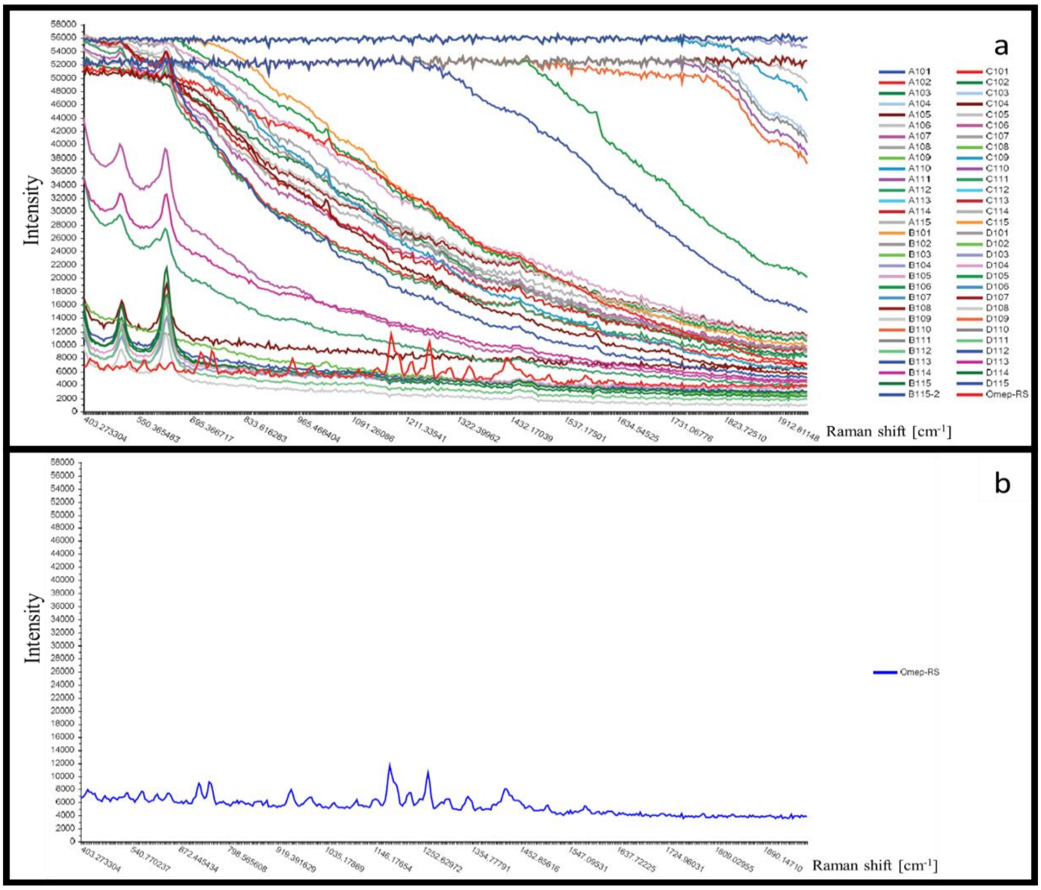

Legend: Supplementary Fig. S14 shows the line plot of the Raman spectra of a: all 60 ESM samples and the omeprazole reference standard (Omep-RS) and b: the Omep-RS only. Spectral data were obtained using an ultra-compact Raman spectrometer C13560. A total of 50 spectral data, scanned on five randomly selected positions each on the tablet upside and tablet downside, were averaged each to obtain the spectra. The Omep-RS, available as powder, was placed in a plastic bag and piled up and scanned in the same manner as the tablets. No corresponding peaks are visible in all 60 spectra. The spectra are presented with colouring according to the sample and the Omep-RS.

ESM – Inspector500 analysis:

Supplementary Fig. S15: Raman spectra of all 60 ESM samples and the Omeprazole reference standard obtained using an Inspector500 portable Raman spectrometer.

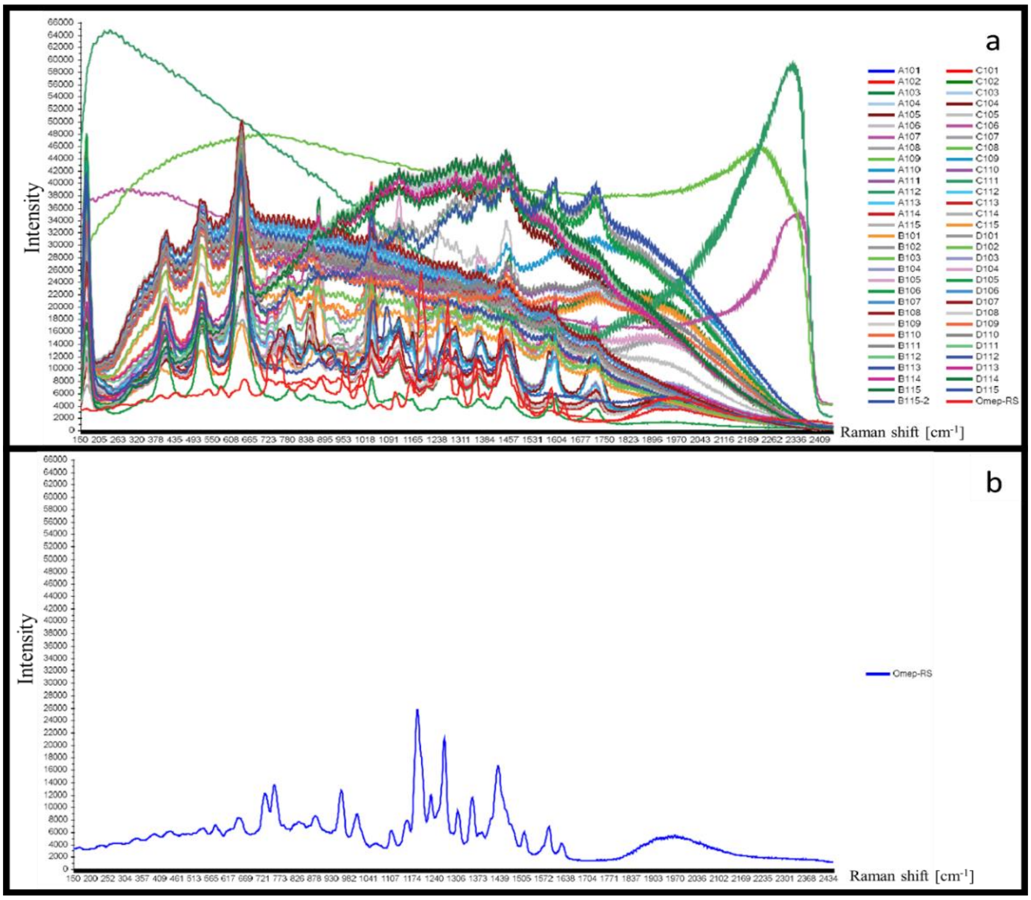

Legend: Supplementary Fig. S15 shows the line plot of the Raman spectra of a: all 60 ESM samples and the omeprazole reference standard (Omep-RS) and b: the Omep-RS only. Spectral data were obtained using an Inspector500 portable Raman spectrometer. A total of 50 spectral data, scanned on five randomly selected positions each on the tablet upside and tablet downside, were averaged each to obtain the spectra. The Omep-RS, available as powder, was placed in a plastic bag and piled up and scanned in the same manner as the tablets. No corresponding peaks are visible in all 60 spectra. The spectra are presented with colouring according to the sample and the Omep-RS.
